# Electrophotochemical Decarboxylative C−H Fluoroalkylation via a Fe_2_O_3_−FTO Active Photoanode

**DOI:** 10.1021/acscatal.5c07973

**Published:** 2026-03-12

**Authors:** Vladimir Motornov, Zhi Liu, Kentaro Imaoka, Sven Trienes, Hyotaik Kang, R. Thomas Weitz, Lutz Ackermann

**Affiliations:** † Wöhler Research Institute for Sustainable Chemistry (WISCh), 9375 Georg-August-Universität Göttingen, Tammannstraße 2, 37077 Göttingen, Germany; ‡ First Institute of Physics and ICASEC, 9375 Georg-August-Universität Göttingen, Friedrich-Hund-Platz 1, 37077 Göttingen, Germany; § German Center for Cardiovascular Research (DZHK),Potsdamer Strasse 58, 10785 Berlin, Germany

**Keywords:** iron, electrophotochemistry, photoanode, fluoroalkylation, C−H functionalization

## Abstract

C−H fluoroalkylation of heteroarenes and biomolecules employing inexpensive carboxylic acids based on electrophotochemical oxidation on a Fe_2_O_3_ photoanode was developed. Notably, the devised approach proved amenable to a wide range of versatile late-stage C−H fluoroalkylations of biorelevant heterocycles. Mechanistic studies supported a direct electron transfer to form a fluoroalkyl radical with a cost-efficient heterogeneous iron catalyst.

## Graphical abstract

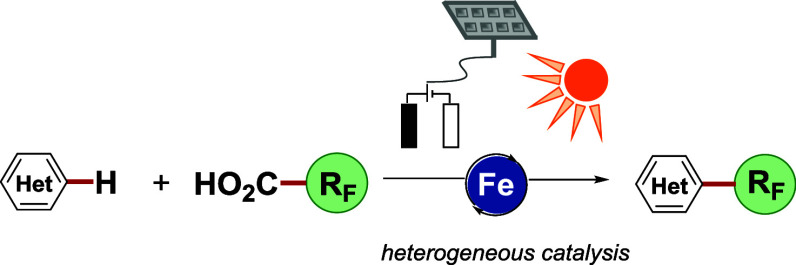

## Introduction

Visible light photocatalysis has surfaced as a transformative platform for molecular synthesis to achieve single-electron-transfer-enabled reactions. Despite major advances, photocatalyzed functionalization reactions often suffer from expensive catalysts or stoichiometric amounts of chemical oxidants.^1–7^ In contrast, electrochemistry is a versatile tool for oxidative processes to achieve the transformation while bypassing the use of undesired chemical oxidants.^8–10^ Therefore, electrochemical processes often feature the cathodic hydrogen evolution reaction (HER) as a valuable counter-reaction. In particular, direct electron transfer using heterogeneous catalysis on an active photoanode is especially attractive due to its operational simplicity and applicability to larger-scale processes.^11^

Fluoroalkylation plays a crucial role in drug development to adjust and enhance the desired pharmacological properties, reflected by the successful implementation of numerous pharmaceuticals bearing fluoroalkyl moieties (Figure 1A).^11–19^ Hence, fluoroalkyl and, in particular, trifluoromethyl moieties can significantly alter lipophilicity, acidity, bioavailability, metabolic stability, and biological activity.^11,18,20^ The most attractive strategy for C−H functionalization would arguably be based on inexpensive carboxylic acids as readily available fluoroalkyl feedstocks,^21^ thereby combining a cost-effective building block with an easily available catalyst and an efficient oxidation. Very recently, we have designed a homogeneous decarboxylative fluoroalkylation catalyzed by an easily available iron­(III) salt,^22^ while Nocera reported electrophotocatalytic trifluoromethylation via elegant silver­(II) catalysis.^23^ Despite these major advances, radical formation from fluoroalkylated acids continues to be a major challenge due to their relatively high oxidation potentials,^24^ which often lead to poor functional group tolerance, along with undesired carboxylate-derived byproducts.^25^ In this context, a complex photoanode based on WO_3_ grown on FTO (fluorine-doped tin oxide) glass was recently designed for efficient photoelectrochemical trifluoromethylation with trifluoroacetic acid (TFA).^26^ However, the relatively high price and low abundance of tungsten and molybdenum may limit their practical and industrial use. Alternatively, the heterogeneous Rh-TiO_2_ photocatalyst in combination with a Na_2_S_2_O_8_ oxidant under 365 nm light was used for TFA activation.^27^ In contrast to 4d and 5d-metal catalysts, the use of 3d metals in homogeneous catalysis has experienced a significant growth in the last two decades.^28–30^ Iron is the most abundant transition metal in the Earth’s crust (Figure 1B), and hence, the metal of choice for catalysis applications.^31–33^ Additionally, its inherent exceptionally low toxicity, as reflected by missing regulations for the permitted daily exposure (PDE) by the International Council for Harmonisation (ICH) Q3D guideline of elemental impurities in common drugs, renders iron specifically interesting for pharmaceutical applications.^34^ In particular, it is clearly superior to tungsten, which belongs to the toxic metals group with a PDE of 5.0 μg/day. Thus, we were fascinated by the opportunity to exploit iron catalysis in a heterogeneous process. As a result, we herein report a heterogeneous C−H fluoroalkylation enabled by iron­(III) oxide grown on an FTO glass photoanode. The approach proved amenable to a broad range of fluoroalkyl radicals featuring fluorinated carboxylic acids as well as arenes, heteroarenes, and pharmaceutically relevant molecules.

**Figure 1:**
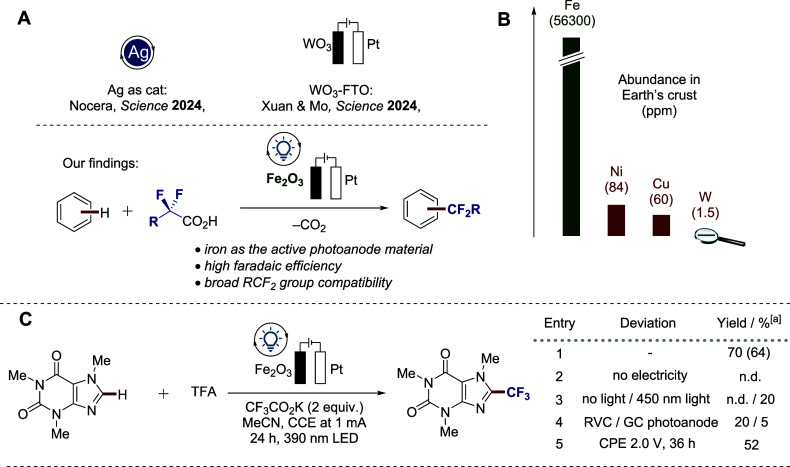
Motivation and strategy for the late-stage C−H fluoroalkylation**.** (A) Recent approaches. (B) Natural abundance of iron and other common transition metals in comparison. (C) Optimization of reaction conditions with the Fe_2_O_3_ photoanode and control experiments. ^a^Yields were determined by ^19^F-NMR using trifluoromethylbenzene as the internal standard. Yield of isolated product is given in parentheses.

## Results and Discussion

### Fe_2_O_3_−FTO Active Photoanode Synthesis and Characterization

Inspired by the hypothesis of using iron­(III) oxide as a heterogeneous catalyst material, we wondered whether a Fe_2_O_3_ deposition of a semiconductor on the FTO glass would be suitable. While the use of an iron oxide photoanode has been reported recently for oxygen-transfer reactions such as alkene epoxidation,^35,36^ its application for decarboxylation processes has never been reported. Preparation of the photoanode was accomplished by the decomposition of iron­(III) chloride in the presence of sodium nitrate, followed by annealing at an elevated temperature. However, all known literature procedures failed to afford a photoanode of reasonable quality to be used for TFA activation. Therefore, optimization of the photoanode preparation, including reaction time, temperature, pH, and additives, was conducted. Preheating of the iron­(III) salt at 120 °C for 6 h, followed by annealing, afforded the best results. After an extensive optimization of the electrophotochemistry setup and parameters (see SI), the desired trifluoromethylation of caffeine was achieved in 70% yield (Figure 1C, entry 1). Control experiments showed that both electricity and light are essential for the heterogeneous photoelectrocatalysis to proceed (entries 2−3), while a 450 nm LED irradiation afforded inferior performance. Importantly, carbon-based photoanodes were found to be significantly less efficient for TFA activation (entry 4). Apart from the most user-friendly galvanostatic conditions, potentiostatic conditions (2.0 V) could be successfully used, which is especially important considering the functionalization of multifunctional substrates (entry 5).

Optical characterization of the synthesized Fe_2_O_3_ (Figure 2A) via UV/Vis spectroscopy (Figure 2B) and the Tauc plot analysis (Figure 2C) revealed a band gap of 2.05 eV, consistent with the known value of hematite.^37^ The energy dispersive X-ray (EDX) spectrum of the Fe_2_O_3_−FTO anode confirmed the presence of iron and oxygen; however, it exhibited a predominant Sn signal (see SI for full details). This signal is likely attributed to a very thin layer of iron oxide, resulting in electron beam penetration into the FTO substrate. Beyond morphological effects, this Sn signal could signify the diffusion of Sn^4+^ ions into the Fe_2_O_3_ lattice during thermal annealing, a process which can enhance the charge transfer kinetics in hematite without compromising the structural integrity of the FTO.^38^ These results are further supported by survey X-ray photoelectron spectroscopy (XPS) and high-resolution XPS of the Fe 2p region (see SI for full details).

**Figure 2:**
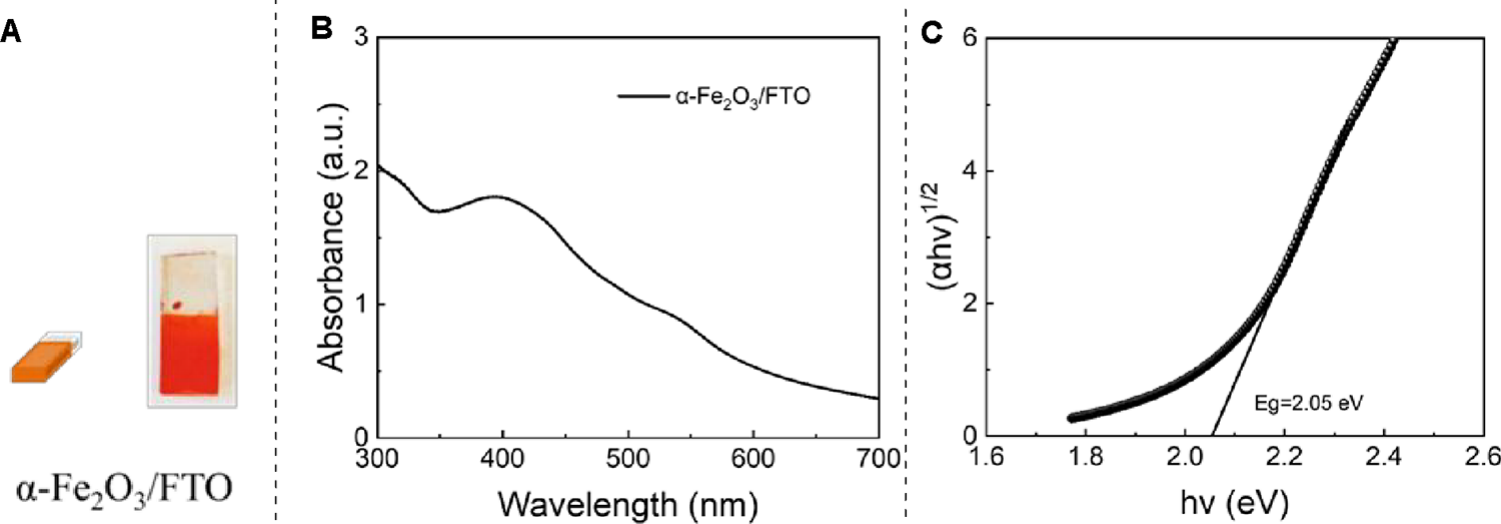
Characterization of Fe_2_O_3_-FTO photoanode**.** (A) Appearance of the photoanode. (B) UV/Vis spectroscopy analysis of the photoanode. (C) Determination of a band gap via Tauc plot analysis.

### Electrophotochemical C−H Fluoroalkylation via Fe_2_O_3_-FTO Photoanode

Intrigued by the resource-economic nature of the uncovered ferra-photoelectrocatalysis manifold, we examined the compatibility of diverse heteroarenes and fluoroalkyl carboxylic acids for C−H fluoroalkylation using a photoanode (Figure 3). To our delight, the reaction proved efficient for the functionalization of caffeine using TFA or related perfluoropropionic and perfluorobutanoic acids to afford desired products **3**−**5** in up to 75% yield. Moreover, 3*H*-tetrafluoropropionic acid (known as the widely used herbicide Flupropanat) gave the fluoroalkylation product **6** as well. A slightly lower efficiency and a moderate yield of 44% were obtained for less electron-deficient 2,2-difluoropropionic acid (**7**). Our strategy proved applicable even to simple arenes, such as unsubstituted benzene (**8**) and electron-rich mesitylene (**9**). Next, we turned our attention to fluoroalkylations of nucleobase derivatives and analogues. 1,3-Dimethyluracil was trifluoromethylated in moderate yield (**10**), whereas a better result was achieved for more electron-deficient 6-azauracil, which is less prone to overoxidation (**11**). Similarly to trifluoromethylation, related azauracil derivatives **12−13** were successfully obtained by simply varying the carboxylic acid source. The methylated orotic acid derivative was efficiently trifluoromethylated in a high 70% yield (**14**). Additionally, the ferra-photoelectrocatalysis exhibited good tolerance of sensitive functional groups, such as dioxolane and free ketone, as shown by the late-stage modification of doxofylline (**15**) and pentoxifylline (**16**), respectively. A sulfur-containing thiouracil derivative, which could be prone to overoxidation issues, was also successfully trifluoromethylated under potentiostatic conditions (**17**). An electron-deficient arene bearing ester groups was well-tolerated under otherwise identical reaction conditions, thus furnishing product **18**. An attempt to apply the strategy to donor−acceptor acetophenone led to C­(sp^3^)−H functionalization (**19**) next to the carbonyl group, rather than the expected ring trifluoromethylation, which can be attributed to the stability of the enol form of acetophenone.^39^

**Figure 3:**
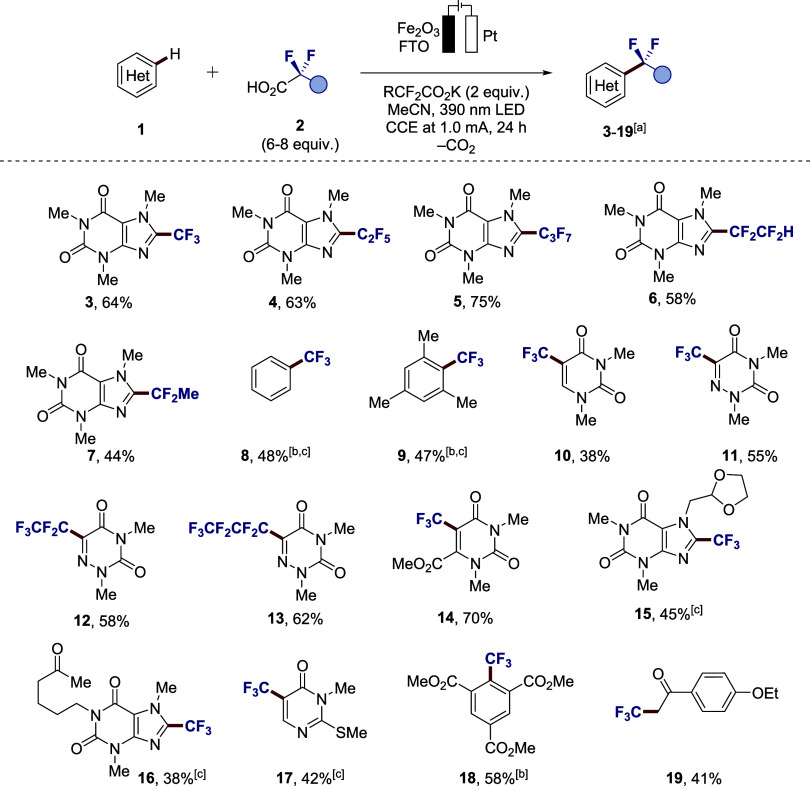
Substrate scope of heterogeneous photoanode trifluoromethylation of heteroarenes**.** Reaction conditions: Undivided cell; heteroarene **1** (0.30 mmol), fluorinated carboxylic acid **2** (1.8−2.4 mmol), potassium salt (0.60 mmol), dry MeCN (total volume 3 mL), Fe_2_O_3_−FTO photoanode, and platinum wire (Pt) cathode. CCE at 1.0 mA, 390 nm light irradiation, 1000 rpm stirring rate. ^a^Isolated yields are indicated unless indicated otherwise. ^b^Yield was determined by ^19^F-NMR. ^c^Potentiostatic conditions (CPE at 2.0 V and 36 h) were used instead of galvanostatic.

### Mechanistic Studies and Photoanode Recycling Tests

With the scope of fluoroalkylated arenes and biomolecules in hand, we turned our attention to probing the mechanism and mode of operation of our heterogeneous ferra-electrocatalysis system. Notably, the Faradaic efficiency of the heterogeneous procedure turned out to be between 45 and 60%, which is higher than previously observed for the homogeneous iron­(III) catalysis.^21^

A trapping experiment revealed that the model reaction was completely suppressed upon the addition of 2,2,6,6-tetramethylpiperidine-1-oxyl (TEMPO) as the radical scavenger (Figure 4A). Notably, the analysis of the reaction mixture by high-resolution mass spectrometry (HRMS) confirmed the presence of the TEMPO−CF_3_ adduct, which supports direct electron transfer during oxidation of the carboxylic acid at the active photoanode, followed by decarboxylation. With these results, we gained insight into the operating mode of the system via linear sweep voltammetry (LSV) analysis (Figure 4B). At negative potentials, an increase in the current was suggestive of the cathodic HER, visually present by significant gas evolution at the platinum cathode. Additionally, the formation of molecular hydrogen was confirmed qualitatively via gas chromatography (GC) analysis of the headspace, where the peak at 1.55 min corresponded to hydrogen (Figure 4C). Moreover, the photoelectrochemical activity of the photoanode was confirmed at positive potentials and under chopped light illumination (Figure 4B).

With these key results in hand, we propose the following mechanism (Figure 4D). Negatively charged trifluoroacetate anions concentrated around the active anode undergo oxidation via direct electron transfer through interaction with the photogenerated holes to form carboxylate radical **A**. Notably, the homogeneous iron­(III) catalyst afforded low performance under standard conditions (see SI for details), which supports the heterogeneous process. Leaching of iron into the solution was also found to be negligibly low, as confirmed by the inductive coupled plasma mass spectrometry (ICP-MS) analysis. As a counterreaction, the HER occurs via proton oxidation. Next, radical **A** undergoes decarboxylation to form CF_3_ radical **B**, which reacts with a substrate. Further electrooxidation of the forming radical, followed by the loss of a proton, furnishes the target product **3**.

**Figure 4:**
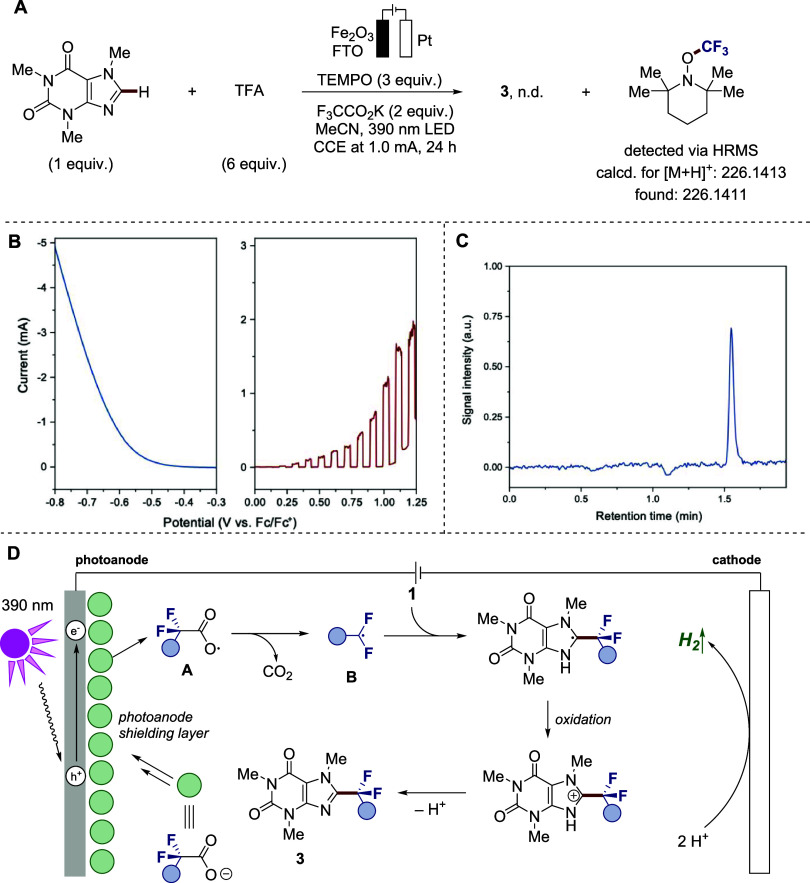
Mechanistic investigations. (A) Radical detection experiments. (B) LSV analysis for the cathodic HER (left) and LSV analysis for the Fe_2_O_3_−FTO photoanode under light-chopped irradiation (right). (C) Detection of molecular hydrogen via GC headspace analysis. (D) Mechanistic proposal.

Next, we tested the reusability of the model photoanode. To our delight, we observed reasonable efficacy upon recycling when potentiostatic conditions were applied (Figure 5A). However, degradation was observed under galvanostatic conditions, especially at potentials above 3.0 V (see SI for details). Finally, we have analyzed the structure of the fresh and spent Fe_2_O_3_−FTO photoanode with scanning electron microscopy (SEM) (Figure 5B), EDX spectroscopy (Figures S6 and S7), and XPS (Figures S8 and S9). Here, we observed the Fe_2_O_3_ nanorods of the original photoanode, which gradually transform their well-dispersed structure after etching in the acidic media,^35,36^ reflecting the formation of the active photoanode material. In addition to the SEM analysis, XPS measurements further support our findings. While no significant changes were detected in the EDX data, the XPS results revealed notable variations in elemental composition. Specifically, the atomic percentage of fluorine increased, whereas that of iron decreased. The fluorine-to-iron ratio (F/Fe) rose markedly from 0.0598 to 0.628 following the reaction. This increase is likely due to the formation of a metal trifluoroacetate complex. Although changes were also observed in the carbon and oxygen contents, it is difficult to accurately determine the resulting chemical formula due to the background signal arising from measurement artifacts. The morphological change observed in the SEM images from rod-like to a more rounded structure is considered to result from the compositional alternation induced by the reaction.

**Figure 5:**
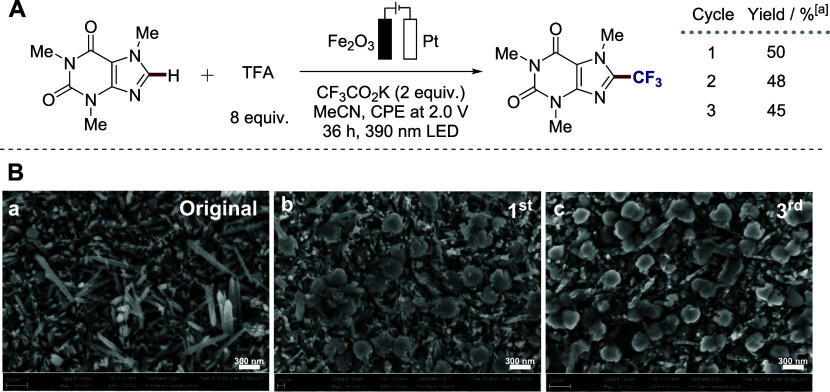
Photoanode stability studies. (A) Recyclability tests. (B) SEM images of the Fe_2_O_3_−FTO photoanode: original (left), after 1st use (middle), and after 3rd use (right). ^a^Yields were determined by ^19^F NMR using trifluoromethylbenzene as the internal standard.

## Experimental Section

### Preparation of the Photoanodes

The FTO glass was cut into a size of 1 cm × 2.5 cm and sequentially ultrasonically cleaned with acetone, ethanol, and isopropanol for 15 min each. A 100 mL aqueous solution containing ferric chloride hexahydrate (4 g) and sodium nitrate (8.5 g) was prepared, and its pH was adjusted to 1∼2 with hydrochloric acid, followed by stirring for 30 min. The cleaned FTO glass was placed in a pressure tube, and the prepared solution was added to submerge two-thirds of the FTO surface. A hydrothermal reaction was conducted at 120 °C for 6 h. Upon completion, the FeOOH/FTO was thoroughly rinsed with deionized water, dried under nitrogen flow, and subsequently placed in an oven at 70 °C for 48 h. Finally, the sample was annealed in a tube furnace at 550 °C for 2 h.

### General Procedure for Fluoroalkylation with Fe_2_O_3_−FTO Photoanode

The mixture of substrate (if solid) (0.3 mmol, 1.0 equiv) and freshly dried potassium salt of the corresponding carboxylic acid (0.6 mmol, 2.0 equiv) charged in an undivided cell equipped with a Pt wire cathode, an Fe_2_O_3_−FTO photoanode, and a small stirring bar (ca. 9 × 2 × 2 mm) were evacuated and backfilled with nitrogen three times. A solution of carboxylic acid (1.8−2.4 mmol, 6.0−8.0 equiv) and a substrate (if liquid) in MeCN (3.0 mL total volume) was added under the positive pressure of nitrogen. The resulting mixture was subjected to constant current electrolysis conditions at 1 mA under irradiation of two 390 nm Kessil lamps, where each lamp was located 3 cm away from the vessel for 24 h unless indicated otherwise. The mixture was then exposed to air and diluted with 20 mL of EtOAc, and 5 mL of 10% aqueous Na_2_CO_3_ solution was added. The aqueous layer was extracted with EtOAc (2 × 30 mL), and the combined organic layers were dried over anhydrous Na_2_SO_4_ and evaporated under reduced pressure (unless noted otherwise for volatile products). The crude product was purified by column chromatography on silica gel.

## Conclusion

In conclusion, we showed that an iron-based heterogeneous photoelectrocatalyst is viable for the direct fluoroalkylation of (hetero)­arenes with inexpensive fluorinated carboxylic acids. Our heterogeneous ferra-photoelectrocatalyst allowed for the late-stage functionalization of medicinally relevant scaffolds with a broad scope. Overall, our findings demonstrate that iron-oxide-doped active photoanodes can enable the C−H functionalization in a cost-efficient manner, which should inspire applications by practitioners in applied industrial settings.

## Supplementary Material



## Notes

Additional experimental details and procedures, characterization data, and spectra (PDF)
