# Supplementary material for: Electrophotochemical Decarboxylative C−H Fluoroalkylation via a Fe2O3−FTO Active Photoanode
Source: ACS Catal. 2026 Mar 12;16(7):6380–6. doi: 10.1021/acscatal.5c07973 (PMC13054784; doi:10.1021/acscatal.5c07973)

Supporting Information

Electrophotochemical Decarboxylative C–H Fluoroalkylation via a Fe_2O_3-FTO Active Photoanode

Vladimir Motornov,[a‡] Zhi Liu,[a‡] Kentaro Imaoka,[b] Sven Trienes,[a,c] Hyotaik Kang,[a] R. Thomas Weitz,[b] and Lutz Ackermann[a,c*]

[a] Wöhler Research Institute for Sustainable Chemistry (WISCh), Georg-August-Universität Göttingen; Tammannstraße 2, 37077 Göttingen, Germany

[b] 1st Institute of Physics and ICASEC, Georg-August-Universität Göttingen, Friedrich-Hund-Platz 1, 37077 Göttingen, Germany

[c] German Center for Cardiovascular Research (DZHK); Potsdamer Straße 58, 10785 Berlin, Germany

‡These authors contributed equally

*Corresponding author: Lutz.Ackermann@chemie.uni-goettingen.de

Table of Contents

General Information 2
Optimization of fluoroalkylation conditions 3
Mechanistic control experiments 4
Preparation and optimization studies of α-Fe_2O_3/FTO photoanode 4
  Optimized procedure for the preparation of photoanode 4
  Optimization of α-Fe_2O_3/FTO photoanode preparation conditions 8
Stability tests of the photoanode 9
  Reusability tests 9
  Summary of Fe_2O_3-FTO photoanode tests (different procedures) under CCE 1.0 mA, 24 h 10
  Analysis of the photoanode samples by energy-dispersive X-ray spectroscopy (EDX) coupled with scanning electron microscopy (SEM) 10
  X-ray photoelectron spectroscopy (XPS) measurements of the photoanode before and after the reaction 12
General procedure 1 for fluoroalkylation using Fe_2O_3-FTO photoanode 13
Radical scavenger experiment 13
Qualitative detection of molecular hydrogen via headspace analysis 13
Linear-sweep voltammetry (LSV) and light-response of photoanode 14
Characterization data 16
List of unsuccessful substrates 21
References 22
NMR spectra 23

General Information

All solvents used for the reactions were HPLC grade. All commercially available chemicals were purchased from commercial suppliers and were used as received with the exception of trifluoroacetic acid. Dry trifluoroacetic acid (TFA) from Fischer Chemical was stored in the presence of 5% of trifluoroacetic anhydride, which was added after opening the bottle to ensure its complete dryness. Electrocatalysis was conducted using a Metrohm MULTI AUTOLAB M204 potentiostat in constant current and constant potential modes. Platinum electrodes (10 mm × 15 mm × 0.25 mm, 99.9%; obtained from ChemPur® Karlsruhe, Germany) and platinum wire (99.9%, 1 mm diameter) were connected using stainless steel adapters. Yields refer to isolated compounds, estimated to be >95% pure as determined by ^{1}H NMR. Column chromatography was performed using silica gel 60 (40–63 µm) from Merck. NMR spectra were recorded on a Bruker Avance III 300 in the solvent indicated; chemical shifts (δ) are given in ppm relative to the residual solvent peak. ^{1}H, ^{13}C and ^{19}F NMR spectra were measured at ambient temperature using 5 mm diameter NMR tubes. ^{13}C NMR spectra were proton decoupled. Coupling constants (J) are reported in Hertz. For ^{19}F NMR yields, $PhCF_3$ was used as an internal standard. All IR spectra were recorded on a Bruker FT-IR Alpha device. High resolution mass spectrometry (HRMS) was measured with APEX IV 7T FTICR. Melting points were measured using Stuart melting point apparatus SMP3, Barloworld Scientific; values are uncorrected. Electrochemical analysis was performed using using the Metrohm Autolab PGSTAT204 potentiostat with the software Nova 2.1. A setup (left) used for the electrophotochemical reactions consisting of two 75 W violet 390 nm LED Kessil lamps located each 3 cm away from the vessel, a potentiostat, two fans and a stirring plate and a cell (right) are shown on Fig. S1 below.

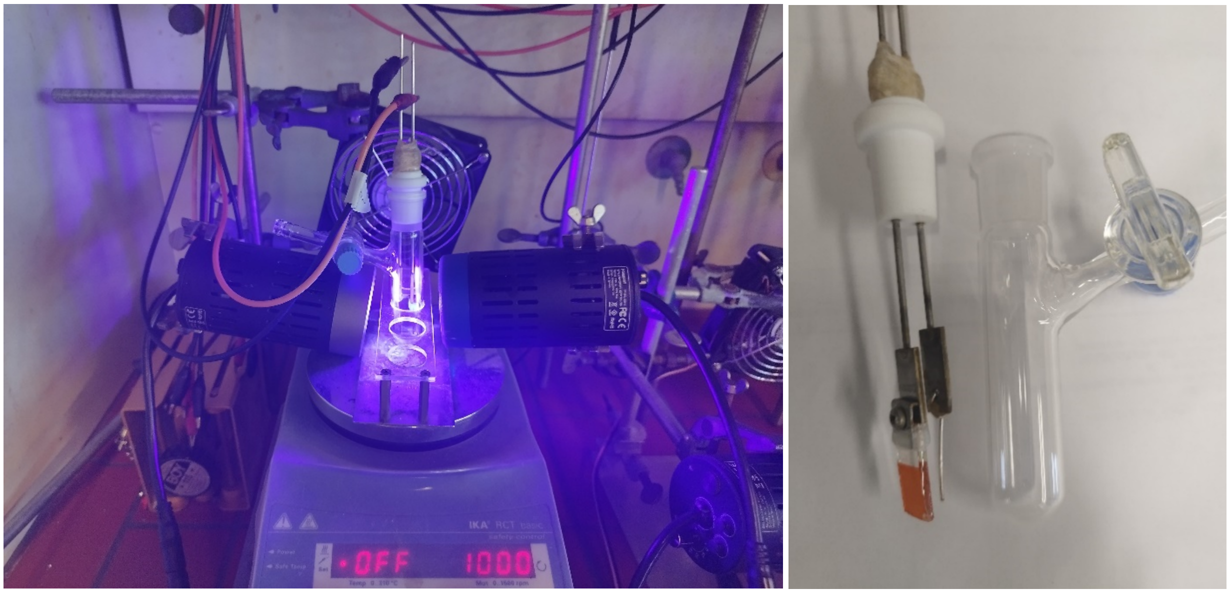

Fig. S1. Setup (left) and undivided cell equipped with Pt wire and Fe_2O_3-FTO photoanode (right) used for the electrophotochemical C–H fluoroalkylation.

Optimization of fluoroalkylation conditions

Optimization studies were conducted as following: caffeine (58.5 mg, 0.3 mmol, 1.0 equiv.) and dried potassium trifluoroacetate (2.0 equiv.) were charged in an undivided cell equipped with Pt wire cathode, Fe_2O_3-FTO photoanode and a small stirring bar (ca. 9 × 2 × 2 mm). Then the tube was evacuated and backfilled with nitrogen 3 times. A solution of TFA (2.4 mmol, 8.0 equiv.) in MeCN (3.5 mL total volume) was added under positive pressure of nitrogen. The resulting mixture was subjected to constant current or constant potential electrolysis under irradiation of two 390 nm Kessil lamps each of them located 3 cm away from the vessel for 24 h. Exact conditions for each entry are indicated below (Table 1). Then the mixture was exposed to air, diluted with 20 mL EtOAc, and 5 mL of 10% aqueous Na_2CO_3 solution was added. Aqueous layer was extracted with EtOAc (4 × 30 mL), combined organic layers were dried over anhydrous Na_2SO_4, and evaporated under reduced pressure. The internal standard ($PhCF_3$) was added, and the mixture was analyzed by ^{19}F NMR.

Table S1. Optimization and control experiments of fluoroalkylation reaction.

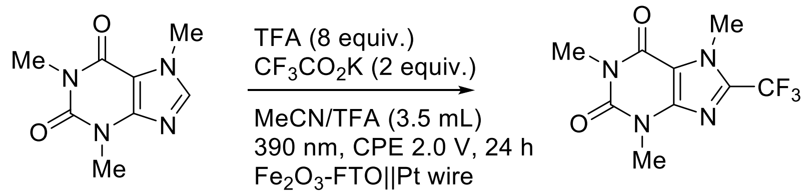

Entry	Deviation from standard	Yield (%)[a]
1	none	37
2	36 h	50
3	CPE 2.2 V	36
4	CPE 2.4 V, 9 h	45
5	CCE 1 mA	58
6	CF_3CO_2Na as electrolyte	21
7	$TBABF_4$ as electrolyte	13
8	$LiClO_4$ as electrolyte	25
**9**	**CCE 1 mA, 0.1 M concentration**	**70 (64)**
10	CCE 1 mA, **2nd cycle**	43
11	CCE 1 mA, **3rd cycle**	27
12	Pt plate instead of wire	29
13	CCE 1 mA, 0.1 M, steel wire instead of Pt wire	45
14	CCE 1 mA, 0.1 M, Ag wire instead of Pt wire	51

[a]Yields were determined by ^{19}F NMR using trifluoromethylbenzene as the internal standard.

Mechanistic control experiments

Control experiments were done using a bare FTO as an anode material without a Fe_2O_3 layer, to test the hypothesis of iron leaching.

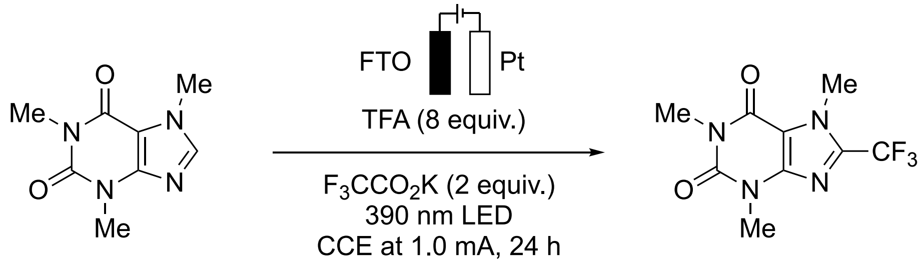

Entry	Deviation from above	Yield (%)[a]
1	20 mol% $Fe(ClO_4)_3 \cdot 9.5H_2O$	20
2	92 ppm Fe using Fe_2O_3 as iron source (with respect to mass of caffeine and F_3CCO_2K)	n.d.

[a]Yields were determined by ^{19}F NMR using trifluoromethylbenzene as the internal standard.

Annotation: Quantitative analysis of the residue after performing the reaction under standard conditions (Tabe S1, entry 9) and the removal of all volatiles via inductively coupled plasma mass spectrometry (ICP-MS) revealed an iron content of 98 ppm. Analysis of the residue after mixing all reagents and the solvent after removal of volatiles but without performing the photoelectrochemical reaction revealed an inherent iron content of 6 ppm. Thus, during the standard reaction an increase of the iron content by 92 ppm due to leaching was observed.

The results indicate low performance of a homogeneous catalyst and no activity of free Fe_2O_3 under standard conditions, thus only very minor influence of leaching on the heterogeneous process is possible.

Preparation and optimization studies of α-Fe_2O_3/FTO photoanode

Optimized procedure for the preparation of photoanode

The FTO glass was cut into the size of 1 cm × 2.5 cm and sequentially ultrasonically cleaned with acetone, ethanol, and isopropanol for 15 minutes each. A 100 mL aqueous solution containing ferric chloride hexahydrate (4 g) and sodium nitrate (8.5 g) was prepared, and its pH was adjusted to 1~2 with hydrochloric acid, followed by stirring for 30 minutes. The cleaned FTO glass was placed in a pressure tube, and the prepared solution was added to submerge two-thirds of the FTO surface. A hydrothermal reaction was conducted at 120 °C for 6 h. Upon completion, the FeOOH/FTO was thoroughly rinsed with deionized water, dried under nitrogen flow, and subsequently placed in an oven at 70 °C for 48 h. Finally, the sample was annealed in a tube furnace at 550 °C for 2 h (Fig. S2).

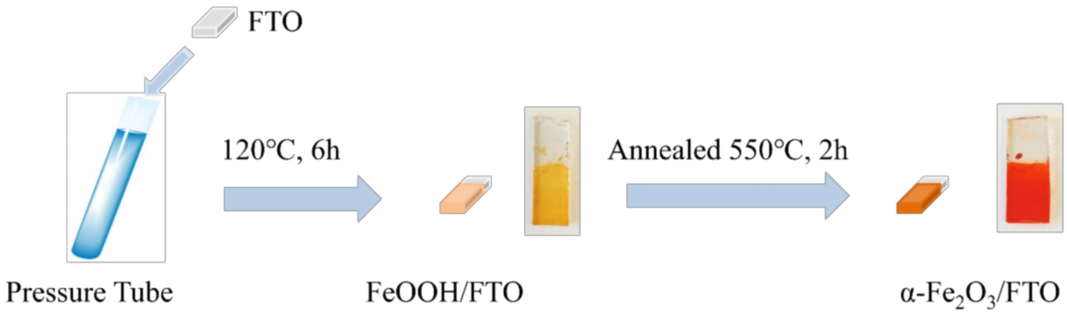

Fig. S2. Schematic diagram of α-Fe_2O_3/FTO preparation process.

The absorption edge of a semiconductor indicates the minimum energy needed to promote an electron from the valence band to the conduction band, corresponding to the bandgap energy (E_g). This bandgap can be estimated by analyzing the onset of absorption in the UV-Vis spectrum (Fig. S3) using the Tauc plot method (Fig. S4). The E_g can be calculated using the following equation:

$$(\alpha h\nu)^n = A(h\nu - E_g)$$

Where *α* is the absorption coefficient (can be approximated from absorbance), *hv* is the photon energy (eV), A is a constant. *n* depends on the electronic transition type: *n* = 1/2 for direct allowed transitions, *n* = 2 for indirect allowed transitions.

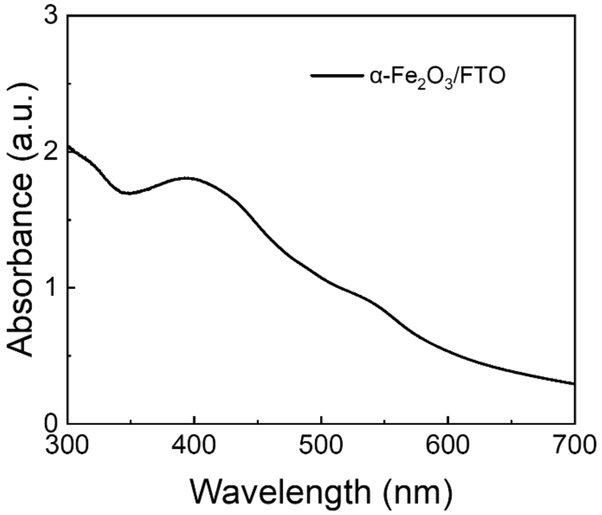

Fig. S3. Absorption spectrum of α-Fe_2O_3.

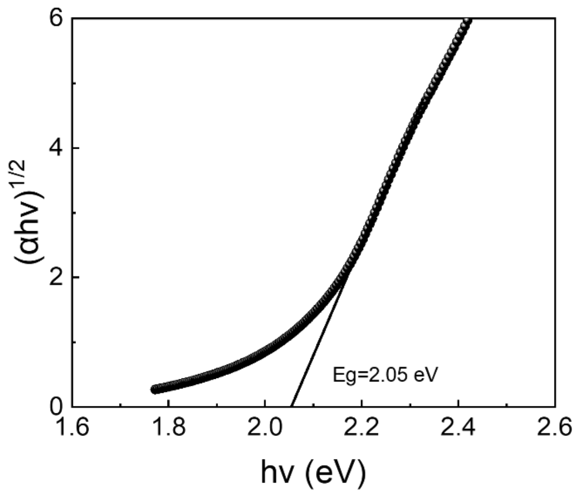

Fig. S4. Tauc plot of a the photoanode obtained from a UV/Vis spectrum.

Through SEM images, it was found that the α-Fe_2O_3 surface has uniform nanorods (Fig. S5), which indicates that the semiconductor material we prepared has a quantum confinement effect. In this regime, the motion of charge carriers (electrons and holes) is spatially restricted, leading to discrete energy levels and an increase in the band gap.[1] Although the band gap of the semiconductor is only 2.05 eV (Fig. S4), it can still produce electron-hole separation under 390 nm light.

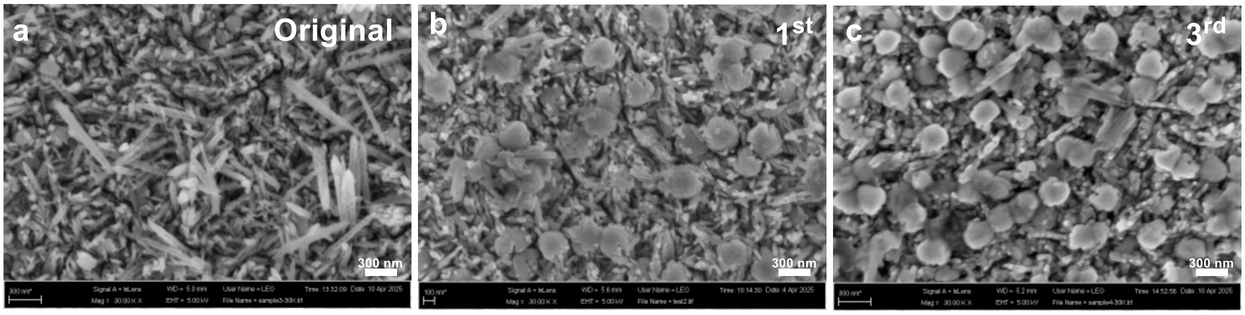

Fig. S5. SEM images of α-Fe_2O_3/FTO photoanode. (a) Original. (b) After first use under CPE at 2.0 V conditions. (c) After third use under CPE at 2.0 V conditions.

The presence of Fe_2O_3 was further corroborated by high-resolution X-ray photoelectron spectroscopy (XPS) analysis of the Fe 2p region. The spectrum exhibits the characteristic doublet with main peaks located at 711 eV and 724 eV corresponding to the Fe 2 $p_{3/2}$ and Fe 2 $p_{1/2}$, respectively. A distinct satellite peak observed at 719 eV serves to further confirm the Fe(III) oxidation state, which is characteristic of Fe_2O_3.[2,3]

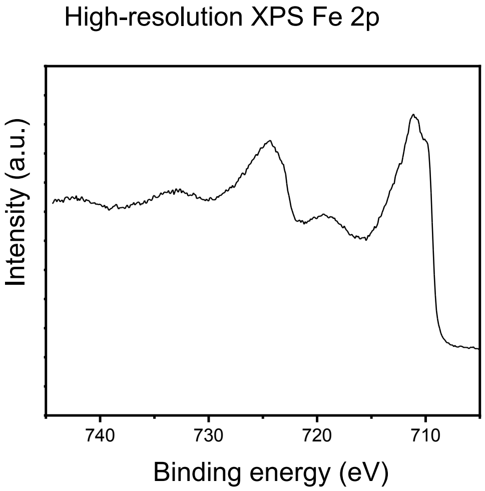

Fig. S6. High-resolution XPS of the Fe 2p region of the photoanode.

The high-resolution Fe 2p XPS spectrum of the Fe_2O_3-FTO sample was deconvoluted to investigate the oxidation states of iron at the surface (Figure S7). The fitting was performed considering both Fe^{3+} and Fe^{2+} species. The binding energies for the main Fe^{3+} doublet were observed at 710.72 eV (Fe $2p_{3/2}$) and 724.08 eV (Fe $2p_{1/2}$), which are in good agreement with literature values for Fe_2O_3.[4,5] Furthermore, the characteristic Fe^{3+} satellite peaks were identified at 718.78 eV and 732.43 eV. The peak fitting also reveals a minor contribution from Fe^{2+} species. The fitting parameters, including peak centers, area integrations, and full width at half maximum (FWHM) values, are summarized in Table S2.

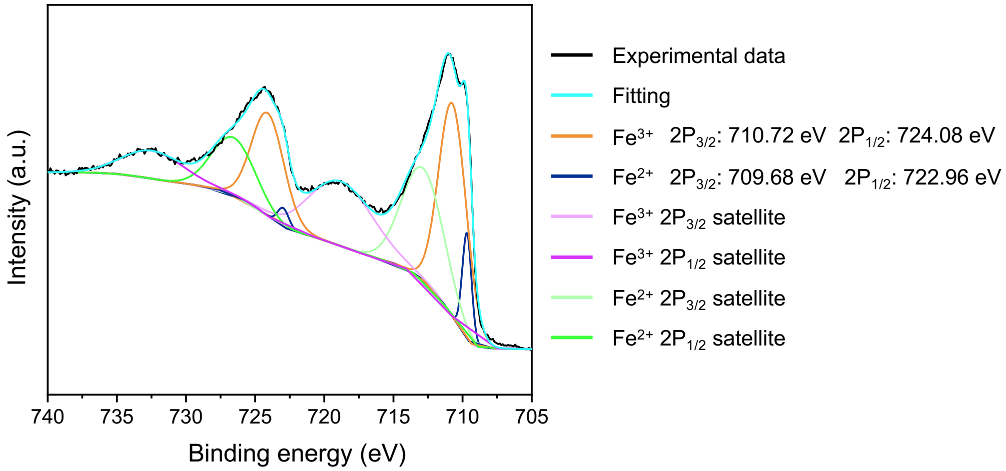

Fig. S7. XPS spectrum of Fe 2p with fitting.

Table S2. Fitting parameters of XPS Fe 2p.

Peak Center	Area Integration	FWHM
709.68365	12379.53037	0.78232
710.72081	75194.31302	2.34592
712.86455	60065.88538	3.57163
718.78024	52617.36793	5.42518
722.96768	2692.08904	0.96633
724.08292	426202.0766	2.76199
726.58141	31484.56178	3.66667
732.4305	21651.78181	5.15956

Optimization of α-Fe_2O_3/FTO photoanode preparation conditions

Effect of temperature and time: During the preparation process, variation of the hydrothermal reaction temperature (95 °C, 120 °C, 140 °C) and time (6 h, 12 h) to obtain different α-Fe_2O_3/FTO photoanodes has been done.

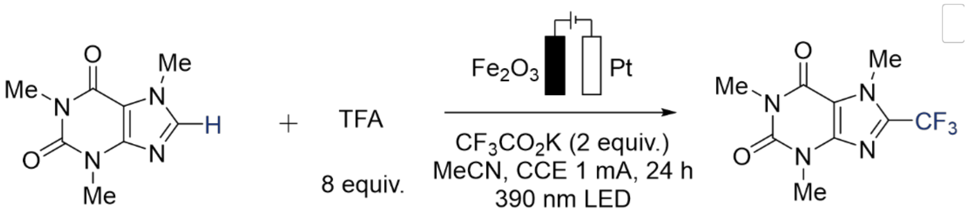

Temperature (°C)	Time (h)	Yield (%)[a]
95	6	58
120	6	70
120	12	40
140	6	38

Reaction conditions: caffeine (0.3 mmol), CF_3COOK (0.6 mmol), TFA (8 equiv.), and MeCN (3 mL), CCE at 1 mA, 24 h. [a] Yields were determined by ^{19}F NMR using trifluoromethylbenzene as the internal standard.

Effect of precursor: The pH of the precursor solution during the preparation process was adjusted to a value of 1-2. It was regulated using various acidic additives, including hydrochloric acid (HCl) or trifluoroacetic acid (TFA) (pH = 1-2). Alternatively, we attempted to replace sodium nitrate ($NaNO_3$) with tetrabutylammonium hydroxide (TBAOH).

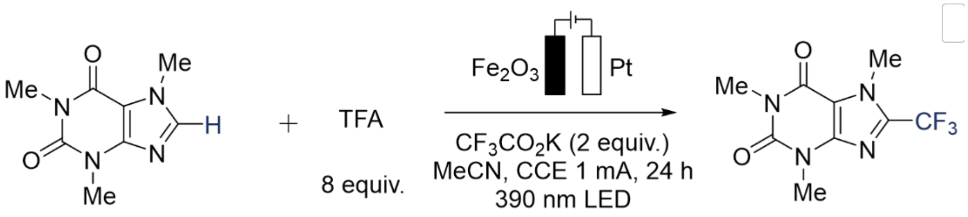

**Precursor**	FeCl$_3$-NaNO$_3$-**HCl**	FeCl$_3$-**TBAOH**-HCl	FeCl$_3$-NaNO$_3$-**TFA**
**Yield (%)[a]**	70	15	47

Reaction conditions: caffeine (0.3 mmol), CF_3COOK (0.6 mmol), TFA (8 equiv.), and MeCN (2.7 mL) with a constant current of 1 mA. The reaction time was 24 h. [a] Yields were determined by ^{19}F NMR using trifluoromethylbenzene as the internal standard.

Experimental results confirmed that the optimized hydrothermal reaction conditions were at a temperature of 120°C, a duration of 6 h and HCl) was employed to adjust the pH of the precursor solution.

Stability tests of the photoanode

Reusability tests

Stability tests of the **best Fe_2O_3-FTO photoanode (procedure 120 °C, 6 h) under CPE 2.0 V**:

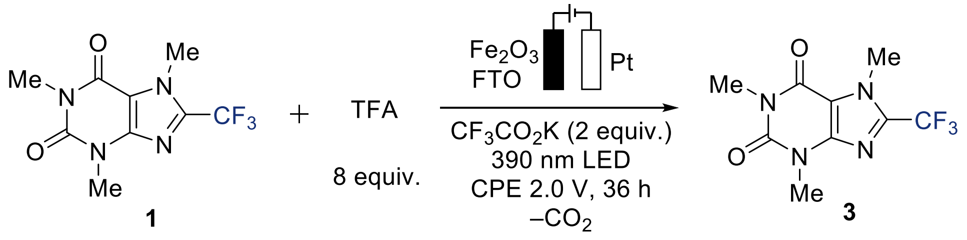

Cycle	NMR yield (%)
1	50
2	48
3	45
4	41

Reaction conditions: caffeine (0.3 mmol), CF_3COOK (0.6 mmol), TFA (8 equiv.), and MeCN (2.7 mL) with a constant potential of 2.0 V. The reaction time was 36 h. Yields were determined by ^{19}F NMR using trifluoromethylbenzene as the internal standard.

Stability of the Fe_2O_3-FTO photoanode under galvanostatic conditions was found to be inferior to potentiostatic conditions, albeit the higher initial yield on the model substrate. Etching of the active Fe_2O_3 surface under potentials above 3.0 V proceeds quickly and diminishes the catalytic potential of the photoanode.

Summary of Fe_2O_3-FTO photoanode tests (different procedures) under CCE 1.0 mA, 24 h

Anode	Fe_2O_3-HCl-FTO (95 °C, 6 h)	Fe_2O_3-TFA-FTO	Fe_2O_3-TBAOH-FTO	Fe_2O_3-HCl-FTO (120 °C, 6 h)	Fe_2O_3-HCl-FTO (120 °C, 12 h)
1st cycle	58	47	15	**59**	40
2nd cycle	43	12		**51**	29
3rd cycle	27			39	

Reaction conditions: caffeine (0.3 mmol), CF_3COOK (0.6 mmol), TFA (8 equiv.), and MeCN (2.7 mL) with a constant current of 1 mA. The reaction time was 24 h. Yields were determined by ^{19}F NMR using trifluoromethylbenzene as the internal standard.

Analysis of the photoanode samples by energy-dispersive X-ray spectroscopy (EDX) coupled with scanning electron microscopy (SEM)

A. Electrode before use

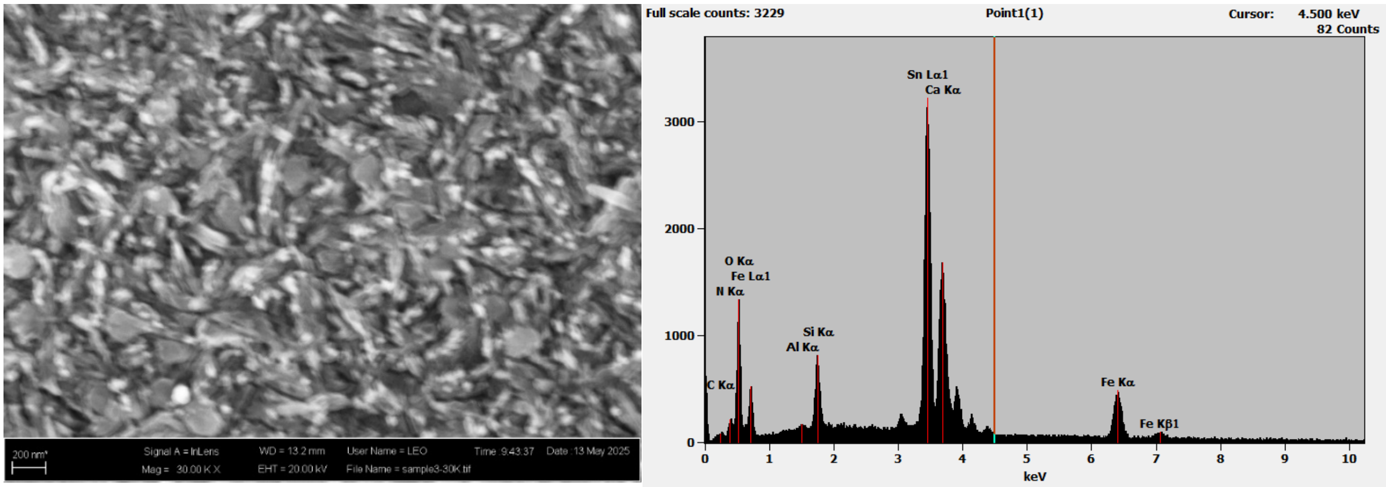

Fig. S7. SEM image and a corresponding EDX spectrum of α-Fe_2O_3/FTO photoanode surface before the reaction (CPE at 2.0 V, 36 h).

Quantitative results

Element Line	*ZAF*	*Atom %*	*Atom % Error*
C K	2.01	0.0	± 0.0
N K	1.77	4.3	± 0.9
O K	5.16	71.5	± 1.7
Si K	1.73	3.2	± 0.1
Ca K	0.99	0.7	± 0.1
Fe K	1.11	5.0	± 0.1
Sn L	1.15	15.4	± 0.2
Total		100.0	

B. Electrode after one time use

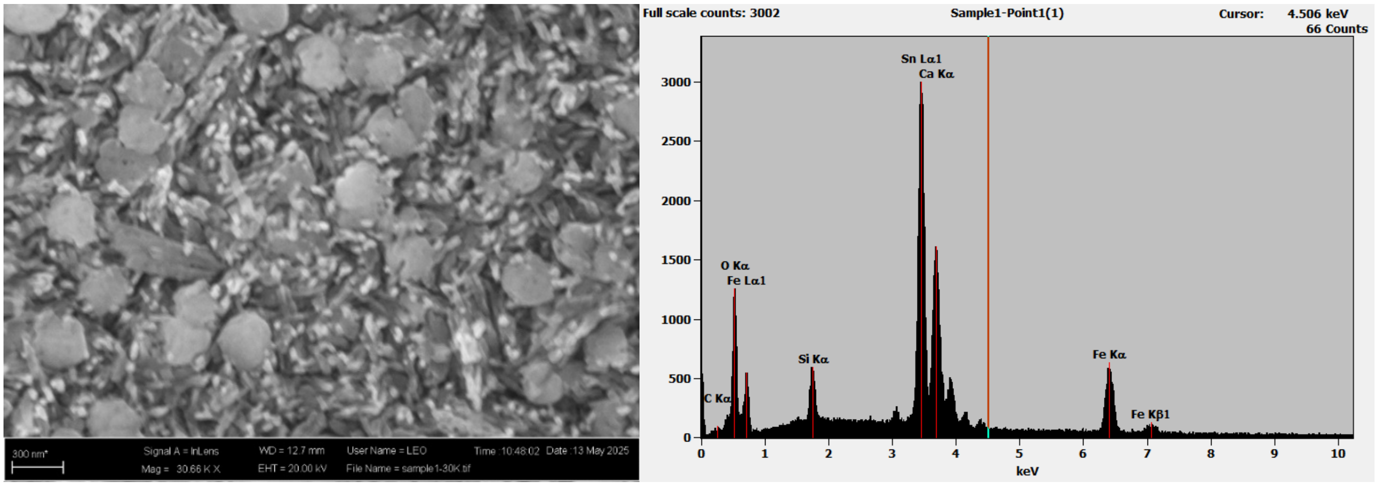

Fig. S8. SEM image and a corresponding EDX spectrum of α-Fe_2O_3/FTO photoanode surface after the reaction (CPE at 2.0 V, 36 h).

Quantitative results

Element Line	*ZAF*	*Atom %*	*Atom % Error*
C K	2.23	5.5	± 1.2
N K	1.98	4.6	± 1.1
O K	5.14	68.3	± 1.7
Si K	1.74	2.3	± 0.1
Ca K	1.00	0.8	± 0.1
Fe K	1.12	5.3	± 0.1
Sn L	1.15	13.2	± 0.1
Total		100.0	

X-ray photoelectron spectroscopy (XPS) measurements of the photoanode before and after the reaction

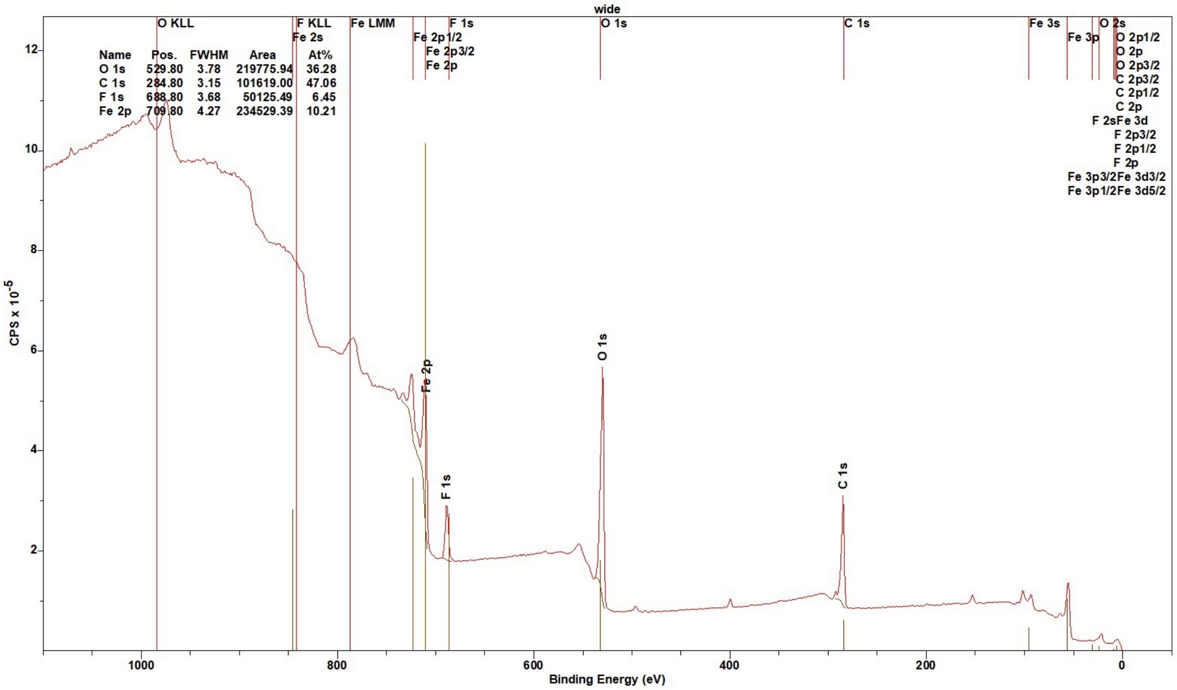

Fig. S9. XPS spectrum of α-Fe_2O_3/FTO photoanode before the reaction.

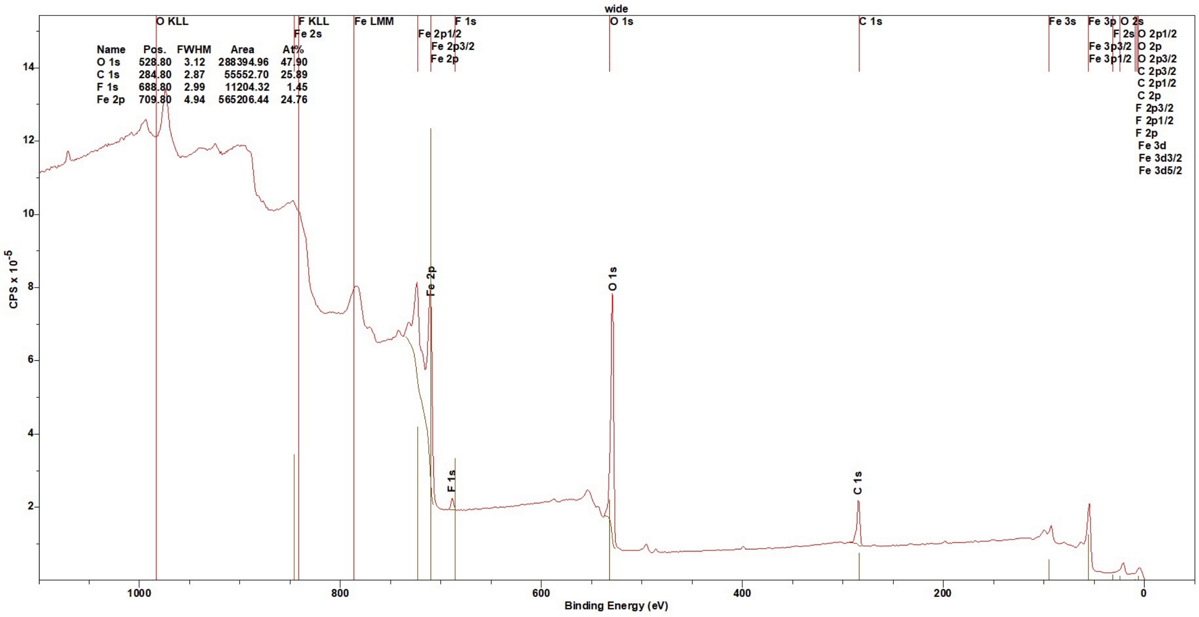

Fig. S10. XPS spectrum of α-Fe_2O_3/FTO photoanode after the reaction (conditions: CPE at 2.0 V, 36 h).

General procedure 1 for fluoroalkylation using Fe_2O_3-FTO photoanode

The mixture of substrate (if solid) (0.3 mmol, 1.0 equiv.) and freshly dried potassium salt of the corresponding carboxylic acid (0.6 mmol, 2.0 equiv.) charged in an undivided cell equipped with Pt wire cathode, Fe_2O_3-FTO photoanode and a small stirring bar (ca. 9 × 2 × 2 mm) was evacuated and backfilled with nitrogen 3 times. A solution of carboxylic acid (1.8-2.4 mmol, 6.0-8.0 equiv. as indicated for each entry below) and a substrate (if liquid) in dry MeCN (3.0 mL total volume) was added under positive pressure of nitrogen. The resulting mixture was subjected to constant current electrolysis conditions at 1 mA under irradiation of two 390 nm Kessil lamps each of them located 3 cm away from the vessel for 24 h unless indicated otherwise. Then the mixture was exposed to air, diluted with 20 mL EtOAc, and 5 mL of 10% aqueous Na_2CO_3 solution was added. Aqueous layer was extracted with EtOAc (2 × 30 mL), combined organic layers were dried over anhydrous Na_2SO_4, and evaporated under reduced pressure (unless noted otherwise for volatile products). Crude product was purified by column chromatography on silica gel using hexane/EtOAc mixtures as an eluent.

Note: To ensure complete dryness of the acid (reaction is moisture-sensitive) 50 µl of Ac_2O could be added to the mixture together with carboxylic acid. In the case of TFA, 5% of TFAA was added to the acid beforehand for the same purpose. Ac_2O and AcOH do not participate in the reaction and do not undergo decarboxylation under standard conditions.

Radical scavenger experiment

The radical scavenger experiment was performed according to general procedure 1. To the solid mixture of reagents and (2,2,6,6-Tetramethylpiperidin-1-yl)oxyl (141 mg, 0.9 mmol, 3.0 equiv.) in the Schlenk flask dry MeCN was added. The photoelectrochemical reaction was performed under 390 nm irradiation and CCE at 1.0 mA. After 24 h, benzotrifluoride was added to the reaction mixture, which was subsequently analyzed by ^{19}F NMR spectroscopy and HRMS.

The analysis via HRMS revealed the formation of TEMPO-CF_3 adduct.

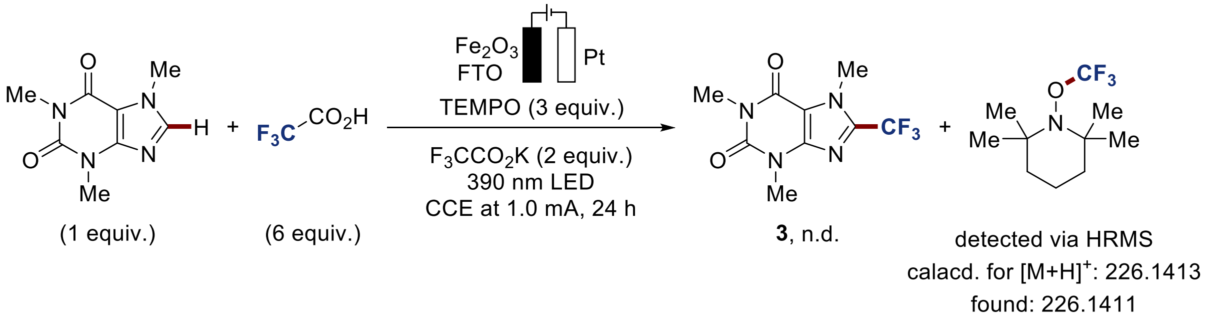

Qualitative detection of molecular hydrogen via headspace analysis

The analysis of the reaction's headspace was performed after running the fluoroalkylation under standard conditions following general procedure 1. Instead of a Teflon cap, the Schlenk tube was sealed with a septum. After photoelectrochemical fluoroalkylation, a sample of 1 mL of the headspace was carefully

collected using a gas syringe. The sample was analyzed using an Agilent 7890B GC System using a Thermal Conductivity Detector and a 5 Å MS column. A characteristic peak a 1.55 min was assigned to molecular hydrogen, as confirmed by the comparison with pure hydrogen as reference sample.

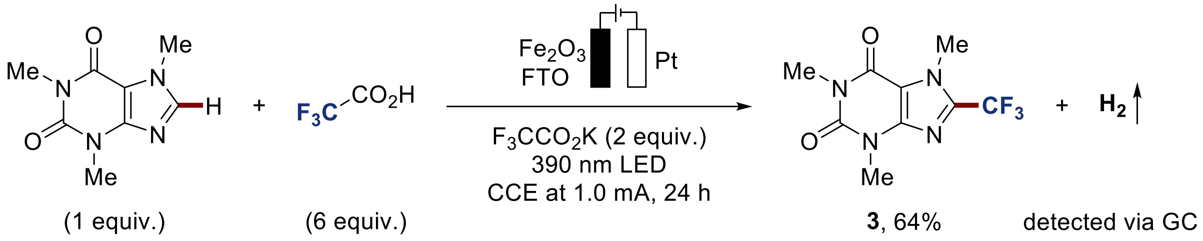

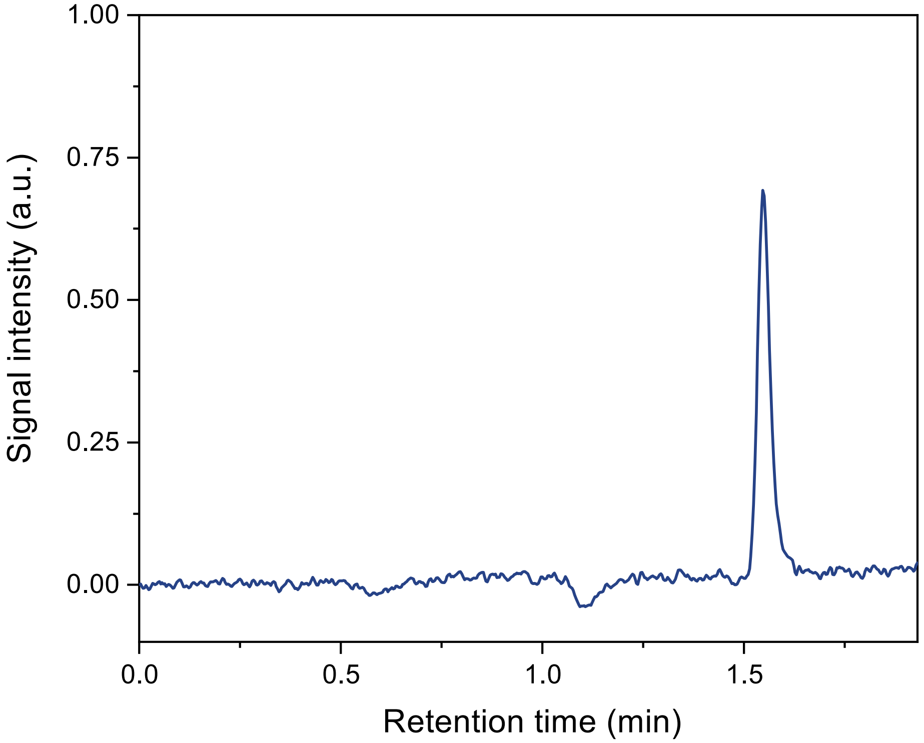

Fig. S11. Excerpt of the GC chromatogram for the headspace analysis.

Linear-sweep voltammetry (LSV) and light-response of photoanode

An electrochemical cell was equipped with the Fe_2O_3-FTO electrode, a coiled platinum wire and a silver wire as reference electrode. Under nitrogen atmosphere, caffeine (97.1 mg, 0.5 mmol, 1.0 equiv.), trifluoroacetic acid (310 µL, 4.0 mmol, 8.0 equiv.) and potassium trifluoroacetate (152 mg, 1.0 mmol, 2.0 equiv.) were dissolved in MeCN (5 mL). The measurements were performed with a scan rate of 10 mV s^{-1} and 390 nm irradiation. For cathodic HER LSV, the platinum wire was used as working electrode and the Fe_2O_3-FTO electrode as photoanode (Fig. S11). For photoelectrochemical characterization of the Fe_2O_3-

FTO electrode, this electrode was used as working electrode with the platinum wire as counter electrode (Fig. S12). To achieve light-chopped irradiation, the 390 nm LED was turned off and on every 5 s.

After (photo)electrochemical analysis, ferrocene was added to the cell and a cyclovoltammetry experiment was performed to determine the Fc/Fc^+ redox wave for referencing.

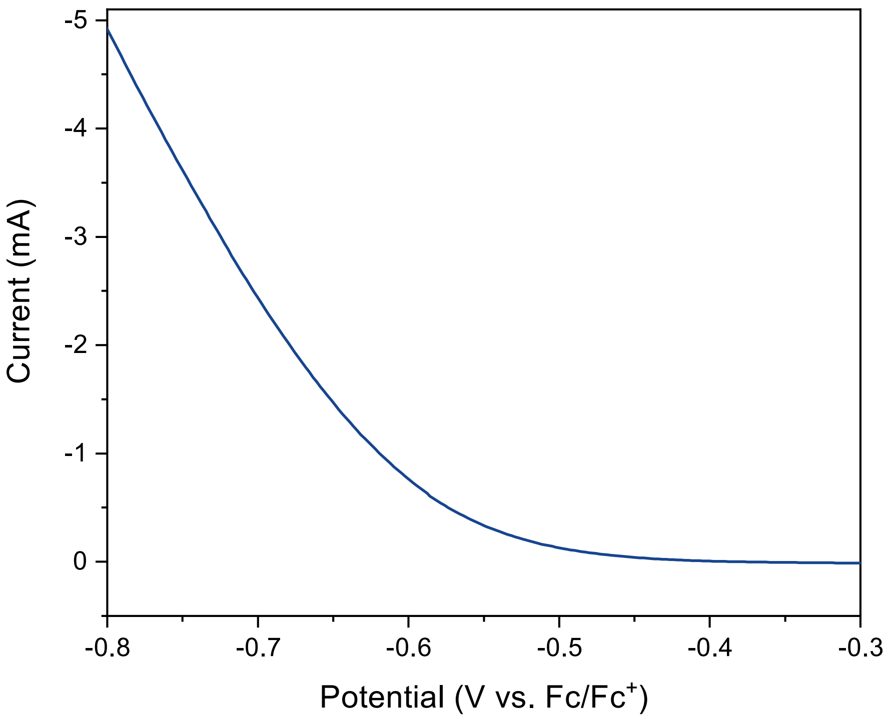

Fig. S12. LSV analysis for cathodic hydrogen evolution reaction.

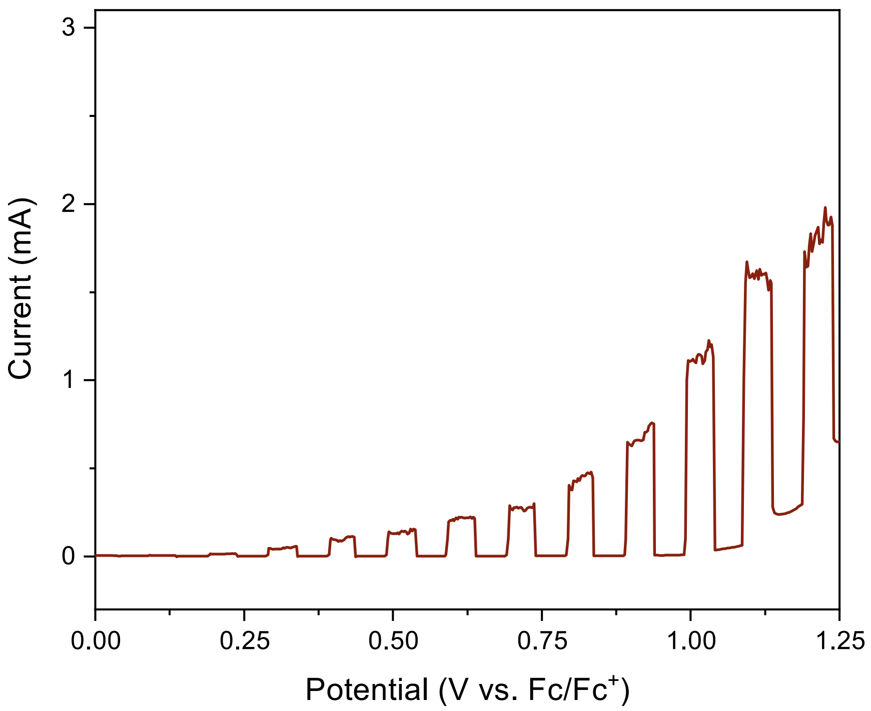

Fig. S13. LSV analysis for the Fe_2O_3-FTO photoanode under light-chopped irradiation.

Characterization data

1,3,7-Trimethyl-8-(trifluoromethyl)-3,7-dihydro-1*H*-purine-2,6-dione (3)

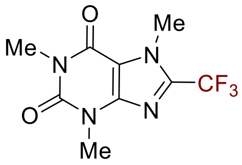

Product **3** was obtained from caffeine (58.5 mg, 0.3 mmol, 1.0 equiv.) and trifluoroacetic acid (8.0 equiv.) according to the general procedure 1. Column chromatography (hexane/EtOAc, 3:1 to 1:1) afforded the title compound (50 mg, 64%) as a white solid.

^{1}H NMR (300 MHz, $CDCl_3$) δ 4.15 (q, J = 1.3 Hz, 3H, Me), 3.58 (s, 3H, Me), 3.41 (s, 3H, Me) ppm; **^{13}C NMR** (75 MHz, $CDCl_3$) δ 155.5, 151.5, 146.6, 139.0 (q, J = 40.3 Hz), 118.3 (q, J = 271.4 Hz), 109.8, 33.3 (q, J = 2.2 Hz, Me), 30.0 (Me), 28.8 (Me); **^{19}F NMR** (282 MHz, $CDCl_3$) δ -62.4 (s, 3F); **m.p.**= 129–130 °C; **IR** (ATR): $\tilde{\nu}$ = 2956, 2926, 1705, 1663, 1546, 1466, 1429, 1172, 1126, 1095 cm^{-1}; **HRMS** (ESI) *m*/*z* calcd for $C_9H_{10}F_3N_4O_2^+$: 263.0750 $[M+H]^+$, found 263.0751.

Characterization details matched previously reported data.[6]

1,3,7-Trimethyl-8-(perfluoroethyl)-3,7-dihydro-1*H*-purine-2,6-dione (4)

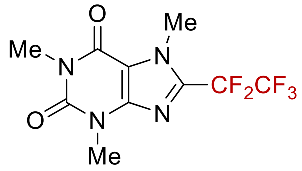

Product **4** was obtained from caffeine (58.5 mg, 0.3 mmol, 1.0 equiv.) and perfluoropropionic acid (6.0 equiv.) according to the general procedure 1. Column chromatography (hexane/EtOAc 4:1 to 3:1) afforded the title compound (59 mg, 63%) as a white solid.

^{1}H NMR (300 MHz, $CDCl_3$) δ 4.18 (t, J = 1.9 Hz, 3H, Me), 3.58 (s, 3H, Me), 3.41 (s, 3H, Me); **^{13}C NMR** (75 MHz, $CDCl_3$) δ 155.4, 151.2, 146.8, 137.6 (t, J = 28.7 Hz, $\underline{C_q}$-CF_2), 118.1

(qt, J = 286.6, 35.8 Hz, CF_3), 110.0, 109.2 (tq, J = 251.0, 40.1 Hz, CF_2), 33.6 (t, J = 4.0 Hz, Me), 29.8 (Me), 28.1 (Me); **^{19}F NMR** (282 MHz, $CDCl_3$) δ -82.7 (t, J = 2.8 Hz, 3F), -111.3 – -111.7 (m, 2F); **m.p.** = 113–115 °C; **IR** (ATR): $\tilde{\nu}$ = 1704, 1670, 1329, 1203, 1156, 1112, 996, 968, 929, 740 cm^{-1}; **HRMS** (ESI) *m*/*z* calcd for $C_{10}H_{10}F_5N_4O_2^+$: 313.0718 [M+H]$^+$, found 313.0723.

1,3,7-Trimethyl-8-(perfluoropropyl)-3,7-dihydro-1*H*-purine-2,6-dione (5)

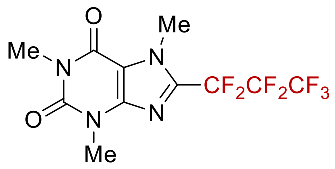

Product **5** was obtained from caffeine (58.5 mg, 0.3 mmol, 1.0 equiv.) and heptafluorobutyric acid (6.0 equiv.) according to the general procedure 1. Column chromatography (hexane/EtOAc 4:1 to 3:1) afforded the title compound (82 mg, 75%) as a white solid.

^{1}H NMR (300 MHz, $CDCl_3$) δ 4.18 (t, J = 1.9 Hz, 3H), 3.58 (s, 3H), 3.41 (s, 3H) ppm; **^{13}C NMR** (75 MHz, $CDCl_3$) δ 155.4, 151.2, 147.0, 137.6 (t, J = 29.0 Hz), 119.6 (qt, J = 288.3, 33.7 Hz, CF_3), 113.0 – 104.4 (m, $CF_2\underline{CF_2}CF_3$), 111.3 (tt, J = 257.2, 32.7 Hz, $\underline{CF_2}CF_2CF_3$), 110.2, 33.8 (dt, J = 4.4, 2.2 Hz, Me), 29.9 (Me), 28.2 (Me) ppm; **^{19}F NMR** (282 MHz, $CDCl_3$) δ -80.0 (t, J = 9.5 Hz, 3F), -109.8 (q, J = 9.5 Hz, 2F), -125.6 (s, 2F) ppm; **m.p.** = 96–98 °C; **IR** (ATR): $\tilde{\nu}$ = 1706, 1671, 1546, 1427, 1343, 1192, 1122, 949, 880, 741 cm^{-1}; **HRMS** (ESI) *m*/*z* calcd for $C_{11}H_9F_7N_4O_2Na^+$: 385.0506 [M+Na]$^+$, found 385.0499.

1,3,7-Trimethyl-8-(1,1,2,2-tetrafluoroethyl)-3,7-dihydro-1*H*-purine-2,6-dione (6)

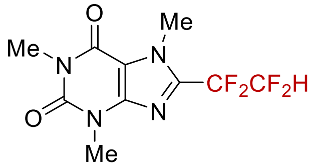

Product **6** was obtained from caffeine (58.5 mg, 0.3 mmol, 1.0 equiv.) and 3*H*-tetrafluoropropionic acid (6.0 equiv.) according to the general procedure 1. Column chromatography (hexane/EtOAc 3:1 to 1:1) afforded the title compound (51 mg, 58%) as a white solid.

^{1}H NMR (300 MHz, $CDCl_3$) δ 6.40 (tt, J = 52.8, 4.8 Hz, 1H, $\underline{H}CF_2CF_2$), 4.18 (t, J = 1.8 Hz, 3H, Me), 3.55 (s, 3H, Me), 3.40 (s, 3H, Me) ppm; **^{13}C NMR** (75 MHz, $CDCl_3$) δ 155.4, 151.3, 146.8, 139.8 (t, J = 28.9 Hz), 110.8 (tt, J = 251.0, 27.4 Hz, CF_2), 109.6, 108.9 (tt, J = 252.0, 32.4 Hz, CF_2H), 33.5 (Me), 29.8 (Me), 28.1 (Me) ppm; **^{19}F NMR** (282 MHz, $CDCl_3$) δ -113.9 (dddd, J = 10.6, 6.7, 4.3, 2.0 Hz, 2F), -137.0 – -137.6 (m, 2F) ppm; **m.p.** = 139–140 °C; **IR** (ATR): $\tilde{\nu}$ = 1712, 1667, 1550, 1455, 1232, 1115, 1009, 820, 763, 742 cm^{-1}. **HRMS** (ESI) *m*/*z* calcd for $C_{10}H_{11}F_4N_4O_2^+$: 295.0813 [M+H]$^+$, found 295.0809.

8-(1,1-Difluoroethyl)-1,3,7-trimethyl-3,7-dihydro-1*H*-purine-2,6-dione (7)

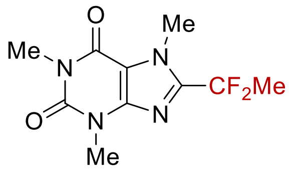

Product **7** was obtained from caffeine (58.5 mg, 0.3 mmol, 1.0 equiv.) and 2,2-difluoropropanoic acid (6.0 equiv.) according to general procedure 1. Column chromatography (hexane/EtOAc 3:1 to 1:1) afforded **7** (34 mg, 44%) as a white solid.

^{1}H NMR (300 MHz, $CDCl_3$) δ 4.15 (t, J = 1.5 Hz, 3H, Me), 3.56 (s, 3H, Me), 3.41 (s, 3H, Me), 2.15 (t, J = 19.1 Hz, 3H, $MeCF_{\underline{2}}$) ppm; **^{13}C NMR** (75 MHz, $CDCl_3$) δ 155.8, 151.7, 146.5, 145.4 (t, J = 32.2 Hz, $\underline{C_q}$–CF_2), 118.5 (t, J = 234.4 Hz, CF_2), 109.4, 33.5 (t, J = 3.7 Hz, Me), 29.9 (Me), 28.2 (Me), 23.1 (t, J = 24.9 Hz, $\underline{Me}CF_2$) ppm; **^{19}F NMR** (282 MHz, $CDCl_3$) δ -87.2 (q, J = 19.1 Hz) ppm; **IR** (ATR): $\tilde{\nu}$ = 1706, 1656, 1547, 1388, 1178, 1120, 915, 902, 749, 664 cm^{-1}; **m.p.** = 153–155°C; **HRMS** (ESI) *m*/*z* calcd for $C_{10}H_{13}N_4O_2F_2^+$: 259.1001 [M+H]$^+$, found 259.1010.

Trifluoromethylbenzene (8)

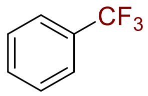

Product **8** was obtained from benzene (23.5 mg, 0.3 mmol, 1.0 equiv.) and trifluoroacetic acid (8.0 equiv.) according to modified general procedure 1 using CPE 2.0 V, reaction time 36 h. Yield determined by ^{19}F NMR after extraction: 48%. Product was not isolated due to its volatility.

^{19}F NMR (282 MHz, $CDCl_3$) δ -63.1 (s, 3F) ppm.

NMR matches previously reported data.[7]

1,3,5-Trimethyl-2-(trifluoromethyl)benzene (9)

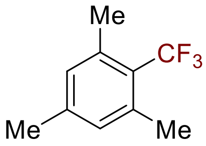

Product **9** was obtained from mesitylene (36 mg, 0.3 mmol, 1.0 equiv.) and trifluoroacetic acid (8.0 equiv.) according to modified general procedure 1 using CPE 2.0 V, reaction time 36 h. Yield determined by ^{19}F NMR after extraction: 47%. Product was not isolated due to its volatility.

^{19}F NMR (282 MHz, $CDCl_3$) δ -54.2 (s, 3F) ppm.

NMR matches previously reported data.[8]

1,3-Dimethyl-5-(trifluoromethyl)pyrimidine-2,4(1*H*,3*H*)-dione (10)

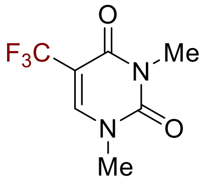

Product **10** was obtained from 1,3-dimethyluracil (42 mg, 0.3 mmol, 1.0 equiv.) and trifluoroacetic acid (8.0 equiv.) according to general procedure 1. Column chromatography (hexane/EtOAc 5:1) afforded the title compound (24 mg, 38%) as a colorless oil.

^{1}H NMR (300 MHz, $CDCl_3$) δ 7.68 (q, *J* = 1.2 Hz, 1H), 3.48 (s, 3H, Me), 3.35 (s, 3H, Me); **^{13}C NMR** (75 MHz, $CDCl_3$) δ 158.8, 151.1, 143.6 (q, *J* = 5.9 Hz), 122.1 (q, *J* = 269.8 Hz), 104.3 (q, *J* = 33.0 Hz), 37.9, 28.2; **^{19}F NMR** (282 MHz, $CDCl_3$) δ -63.8 (d, *J* = 1.0 Hz); **IR** (ATR): $\tilde{\nu}$ = 1722, 1675, 1492, 1460, 1383, 1325, 1212, 1128, 413 cm^{-1}; **HRMS** (ESI) *m/z* calcd for $C_7H_8F_3N_2O_2^+$: 209.0532 $[M+H]^+$, found 209.0533.

Characterization details matched previously reported data.[6]

2,4-Dimethyl-6-(trifluoromethyl)-1,2,4-triazine-3,5(2*H*,4*H*)-dione (11)

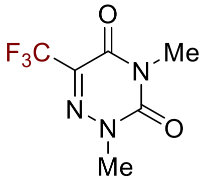

Product **11** was obtained from 1,3-dimethyl-6-azauracil (42 mg, 0.3 mmol, 1.0 equiv.) and trifluoroacetic acid (8.0 equiv.) according to general procedure 1. Column chromatography (hexane/EtOAc 5:1) afforded the title compound (34.5 mg, 55%) as a colorless oil.

^{1}H NMR (300 MHz, $CDCl_3$) δ 3.73 (s, 3H), 3.39 (s, 3H) ppm; **^{13}C NMR** (75 MHz, $CDCl_3$) δ 152.5, 148.6, 130.8 (q, *J* = 35.9 Hz), 119.3 (q, *J* = 273.6 Hz, CF_3), 40.4 (Me), 27.5 (Me); **^{19}F NMR** (282 MHz, $CDCl_3$) δ -67.9 (s, 3F); **IR** (ATR): $\tilde{\nu}$ = 1738, 1688, 1456, 1332, 1254, 1191, 1147, 1016, 745, 408 cm^{-1}; **HRMS** (ESI) *m/z* calcd for $C_6H_7F_3N_3O_2^+$: 210.0485 $[M+H]^+$, found 210.0484.

Characterization details matched previously reported data.[6]

2,4-Dimethyl-6-(perfluoroethyl)-1,2,4-triazine-3,5(2*H*,4*H*)-dione (12)

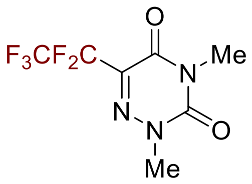

Product **12** was obtained from 1,3-dimethyl-6-azauracil (42 mg, 0.3 mmol) and perfluoropropionic acid (6.0 equiv.) according to general procedure 1. Column chromatography (hexane/EtOAc 6:1) afforded the title compound (45 mg, 58%) as a colorless oil.

^{1}H NMR (300 MHz, $CDCl_3$) δ 3.73 (s, 3H, Me), 3.37 (s, 3H, Me) ppm; **^{13}C NMR** (75 MHz, $CDCl_3$) δ 152.6, 148.5, 130.3 (t, *J* = 26.8 Hz, $\underline{C_q}$-CF_2), 118.5 (qt, *J* = 286.9, 35.6 Hz, CF_3), 110.4 (tq, *J* = 255.0, 38.8 Hz, CF_2), 40.7 (Me), 27.7 (Me) ppm; **^{19}F NMR** (282 MHz, $CDCl_3$) δ -82.0 (s, 3F), -115.6 (s, 2F) ppm; **IR** (ATR): $\tilde{\nu}$ = 1736, 1684, 1590, 1445, 1355, 1207, 1149, 1117, 980, 738 cm^{-1}; **HRMS** (ESI) *m*/*z* calcd for $C_7H_6F_5N_3O_2Na^+$: 282.0272 $[M+Na]^+$, found 282.0267.

2,4-Dimethyl-6-(perfluoropropyl)-1,2,4-triazine-3,5(2*H*,4*H*)-dione (13)

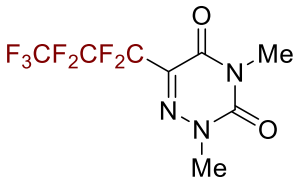

Product **13** was obtained from 1,3-dimethyl-6-azauracil (42 mg, 0.3 mmol, 1.0 equiv.) and heptafluorobutanoic acid (6.0 equiv.) according to general procedure 1. Column chromatography (hexane/EtOAc 6:1) afforded the title compound (57.5 mg, 62%) as a colorless oil, which solidifies upon storage.

^{1}H NMR (300 MHz, $CDCl_3$) δ 3.73 (s, 3H, Me), 3.37 (s, 3H, Me) ppm; **^{13}C NMR** (75 MHz, $CDCl_3$) δ 152.6, 148.5, 130.3 (t, *J* = 25.7 Hz), 123.5 – 105.1 (m, C_3F_7), 40.7, 27.7 ppm; **^{19}F NMR** (282 MHz, $CDCl_3$) δ -80.2 (s, 3F), -113.4 (q, *J* = 9.4 Hz, 2F), -125.2 (m, 2F) ppm; **IR** (ATR): $\tilde{\nu}$ = 1736, 1685, 1445, 1345, 1207, 1118, 911, 736, 723, 524 cm^{-1}; **HRMS** (ESI) *m*/*z* calcd. for $C_8H_6N_3O_2F_7Na^+$: 332.0240 $[M+Na]^+$, found 332.0233.

Methyl 1,3-dimethyl-2,6-dioxo-5-(trifluoromethyl)-1,2,3,6-tetrahydropyrimidine-4-carboxylate (14)

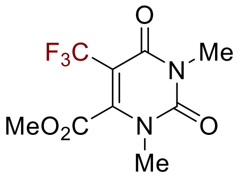

Product **14** was obtained from methyl 1,3-dimethyl-2,6-dioxo-1,2,3,6-tetrahydropyrimidine-4-carboxylate (59.5 mg, 0.3 mmol, 1.0 equiv.) and trifluoroacetic acid (8.0 equiv.) according to general procedure 1. Column chromatography (hexane/EtOAc 1:1) afforded the title compound (56 mg, 70%) as a yellow solid.

^{1}H NMR (300 MHz, $CDCl_3$) δ 4.00 (s, 3H, CO_2Me), 3.36 (s, 6H, Me) ppm; **^{13}C NMR** (75 MHz, $CDCl_3$) δ 172.7, 160.8, 158.4, 150.3, 146.5 (q, *J* = 3.3 Hz), 121.6 (q, *J* = 272.5 Hz), 101.5 (q, *J* = 32.6 Hz,), 54.4 (CO_2Me), 33.7, 28.5 ppm; **^{19}F NMR** (282 MHz, $CDCl_3$) δ -60.3 (s, 3F) ppm; **m.p.** = 128–130°C; **IR** (ATR): $\tilde{\nu}$ = 1709, 1663, 1435, 1246, 1171, 1125, 1036, 745, 500, 412 cm^{-1}; **HRMS** (ESI) *m*/*z* calcd for $C_9H_9N_2O_4F_3Na^+$: 289.0407 $[M+Na]^+$, found 289.0400.

7-(1,3-Dioxolan-2-yl)-1,3-dimethyl-8-(trifluoromethyl)-3,7-dihydro-1*H*-purine-2,6-dione (15)

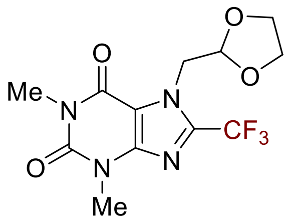

Product **15** was obtained from doxofylline (80 mg, 0.3 mmol, 1.0 equiv.) and trifluoroacetic acid (8.0 equiv.) according to the modified general procedure 1 using CPE 2.0 V, reaction time 36 h. Column chromatography (hexane/EtOAc 1:1) afforded the title compound (43 mg, 45%) as a colorless oil, which solidifies upon storage. Characterization details matched previously reported data [6].

^{1}H NMR (300 MHz, $CDCl_3$) δ 5.34 (t, J = 4.4 Hz, 1H), 4.68 (d, J = 4.4 Hz, 2H), 3.97 – 3.88 (m, 4H), 3.60 (s, 3H), 3.42 (s, 3H); **^{13}C NMR** (75 MHz, $CDCl_3$) δ 155.3, 151.3, 146.7, 139.2 (q, J = 39.9 Hz), 118.2 (q, J = 271.6 Hz), 109.5, 100.9, 65.3, 48.7, 29.9 (Me), 28.3 (Me); **^{19}F NMR** (282 MHz, $CDCl_3$) δ -60.8 (s, 3F); **IR** (ATR): $\tilde{\nu}$ = 2957, 2896, 1710, 1661, 1612, 1545, 1455, 1346, 1267, 1128, 1038 cm^{-1}; **HRMS** (ESI) m/z calcd for $C_{12}H_{13}N_4O_4F_3Na^+$: 357.0781 $[M+Na]^+$, found 357.0781.

3,7-Dimethyl-1-(5-oxohexyl)-8-(trifluoromethyl)-3,7-dihydro-1*H*-purine-2,6-dione (16)

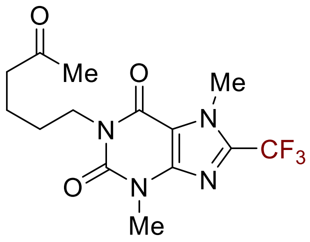

Product **16** was obtained from pentoxyfilline (83.5 mg, 0.3 mmol, 1.0 equiv.) and trifluoroacetic acid (8.0 equiv.) according to the modified general procedure 1 using CPE 2.0 V, reaction time 36 h. Column chromatography (hexane/EtOAc 1:1 to 1:2) afforded the title compound (39.5 mg, 38%) as a colorless oil, which solidifies upon storage.

^{1}H NMR (300 MHz, $CDCl_3$): δ 4.14 (s, 3H), 4.01 (t, J = 6.3 Hz, 2H), 3.56 (s, 3H), 2.49 (t, J = 6.6 Hz, 2H), 2.13 (s, 3H), 1.66 – 1.59 (m, 4H); **^{13}C NMR** (75 MHz, $CDCl_3$): δ 208.6, 155.5, 151.2, 146.7, 139.1 (q, J = 40.2 Hz), 118.3 (q, J = 271.3 Hz), 109.8, 43.2, 41.3, 33.3 (d, J = 2.3 Hz), 31.0, 30.0, 27.5, 21.0; **^{19}F NMR** (288 MHz, $CDCl_3$): δ -62.4 (s, 3F); **IR** (ATR): $\tilde{\nu}$ = 2957, 1708, 1661, 1609, 1547, 1462, 1334, 1247, 1130, 1098 cm^{-1}; **HRMS** (ESI) m/z calcd for $C_{14}H_{18}N_4O_4F_3^+$: 347.1326 $[M+H]^+$, found 347.1327.

Characterization details matched previously reported data.[6]

3-Methyl-2-(methylthio)-5-(trifluoromethyl)pyrimidin-4(*3H*)-one (17)

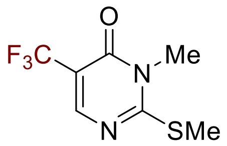

Product **17** was obtained from *N*,*S*-dimethyl-2-thiouracil (48 mg, 0.3 mmol, 1.0 equiv.) and trifluoroacetic acid (8.0 equiv.) according to the modified general procedure 1 using CPE 2.0 V, reaction time 36 h. Column chromatography (hexane/EtOAc 5:1 to 2:1) afforded the title compound (28 mg, 42%) as a colorless oil, which solidifies upon storage.

^{1}H NMR (300 MHz, $CDCl_3$): δ 8.12 (q, J = 1.2 Hz, 1H), 3.54 (s, 3H, Me), 2.62 (s, 3H, Me); **^{13}C NMR** (75 MHz, $CDCl_3$): δ 168.4, 157.9, 150.9 (q, J = 5.4 Hz), 122.9 (q, J = 271.1 Hz), 111.7 (q, J = 31.3 Hz), 30.5 (Me), 15.3 (Me); **^{19}F NMR** (288 MHz, $CDCl_3$): δ -64.9 (s, 3F); **IR** (ATR): $\tilde{\nu}$ = 1693, 1594, 1503, 1415, 1390, 1326, 1129, 1106, 888, 785 cm^{-1}; **HRMS** (ESI) m/z calcd for $C_7H_7N_2F_3OSNa^+$: 247.0123 $[M+Na]^+$, found 247.0123.

Trimethyl 2-(trifluoromethyl)benzene-1,3,5-tricarboxylate (18)

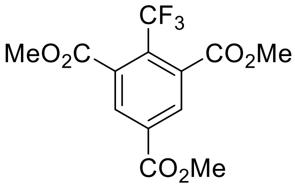

Product **18** was obtained from trimethyl benzene-1,3,5-tricarboxylate (76 mg, 0.3 mmol, 1.0 equiv.) and trifluoroacetic acid (8.0 equiv.) according to the general procedure 1. Yield determined by ^{19}F NMR after extraction: 58%.

^{19}F NMR (282 MHz, $CDCl_3$) δ -56.3 (s, 3F); **IR** (ATR): $\tilde{\nu}$ = 2958, 1730, 1437, 1246, 1212, 1160, 1140, 1000, 744, 721 cm^{-1}; **HRMS** (ESI) *m*/*z* calcd for $C_{13}H_{11}O_6F_3Na^+$: 343.0400 $[M+Na]^+$, found 343.0400.

Characterization details match previously reported data.[7]

3,3,3-Trifluoro-1-(4-ethoxyphenyl)propan-1-one (19)

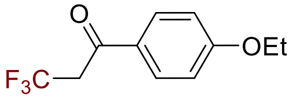

Product **19** was obtained from trimethyl 4-ethoxyacetophenone (49 mg, 0.3 mmol, 1.0 equiv.) and trifluoroacetic acid (8.0 equiv.) according to the modified general procedure 1 using CPE 2.0 V, reaction time 24 h. Column chromatography (hexane/EtOAc 4:1) afforded the title compound (27 mg, 41%) as a yellow solid.

^{1}H NMR (300 MHz, $CDCl_3$) δ 7.89 (d, *J* = 8.8 Hz, 2H), 6.94 (d, *J* = 8.8 Hz, 2H), 4.11 (q, *J* = 7.2 Hz, 2H), 3.73 (q, *J* = 10.0 Hz, 2H), 1.45 (t, *J* = 7.2 Hz, 3H) ppm; **^{13}C NMR** (75 MHz, $CDCl_3$) δ 188.3 (C=O), 163.9, 131.0, 128.9 (m), 124.3 (q, *J* = 277.1 Hz), 114.7, 64.1, 41.9 (q, *J* = 28.1 Hz, CH_2), 14.7 (Me); **^{19}F NMR** (282 MHz, $CDCl_3$) δ -62.0 (s, 3F) ppm; **IR** (ATR): $\tilde{\nu}$ = 2989, 1685, 1599, 1369, 1283, 1269, 1234, 1174, 1115, 1094 cm^{-1}; **HRMS** (ESI) *m*/*z* calcd for $C_{11}H_{12}F_3O_2Na^+$: 255.0603 $[M+Na]^+$, found 255.0604. Characterization details match previously reported data.[9]

List of unsuccessful substrates

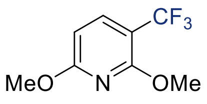

28% (NMR)

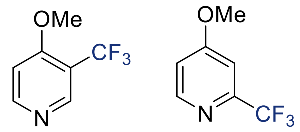

13% total, 1:2 ratio

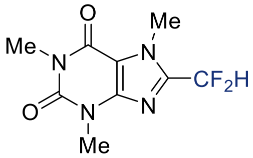

10% (NMR)

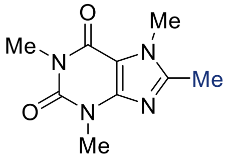

from AcOH
n.d.

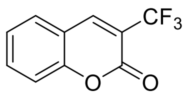

n.d.

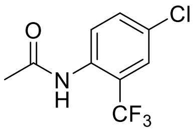

decomposition

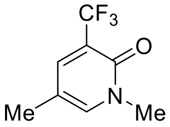

18% (NMR)

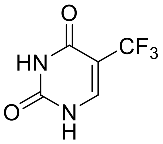

n.d.

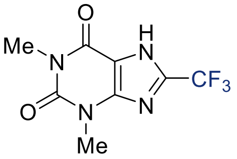

n.d.

References

1. M. B. Sahana, C. Sudakar, A. Dixit, J. S. Thakur, R. Naik, & V. M. Naik, *Acta Materialia*, **60**, 1072–1078 (2012).
2. S. More, S. Raut, S. Premkumar, S. Bhopale, S. Bhoraskar, M. More, V. Mathe, *RSC Adv.*, **10**, 32088–32101 (2020).
3. F. Käufer, A. Quade, A. Kruth, H. Kahlert, *Nanomaterials*, **14**, 252 (2024).
4. Z. Hou, P. Yan, B. Sun, H. Elshekh, & B. Yan, *Results Phys.,* **14**, 102498 (2019).
5. T. Yamashita, & P. Hayes, *Appl. Surf. Sci.*, **254**, 2441–2449 (2008).
6. L. Zuo, G. Qiu, Y. Liu, X. Chen, K. Sun, I. B. Krylov, L. Qu, A. O. Terentiev, & B. Yu, *Chem. Catal.*, **4**, 101–151 (2024).
7. J. Qi, J. Xu, H. T. Ang, B. Wang, N. K. Gupta, S. R. Dubbaka, P. O'Neill, X. Mao, Y. Lum, & J. Wu, *J. Am. Chem. Soc.,* **145**, 24965–24971 (2023).
8. Y. Chen, Y. He, Y. Gao, J. Xue, W. Qu, J. Xuan, & Y. Mo, *Science,* **384**, 670–676 (2024).
9. C.-Y. Wu, X.-L. Chen, H.-Y. Wang, D.-S. Yang, S.-Y. Zhuang, Y. Zhou, Z.-C. Yu, Y.-D. Wu, X. Geng, & A.-X. Wu, *Org. Chem. Front.*, **10**, 3741–3745 (2023).

NMR spectra

1,3,7-Trimethyl-8-(trifluoromethyl)-3,7-dihydro-1*H*-purine-2,6-dione (3)

^{1}H NMR (300 MHz, $CDCl_3$)

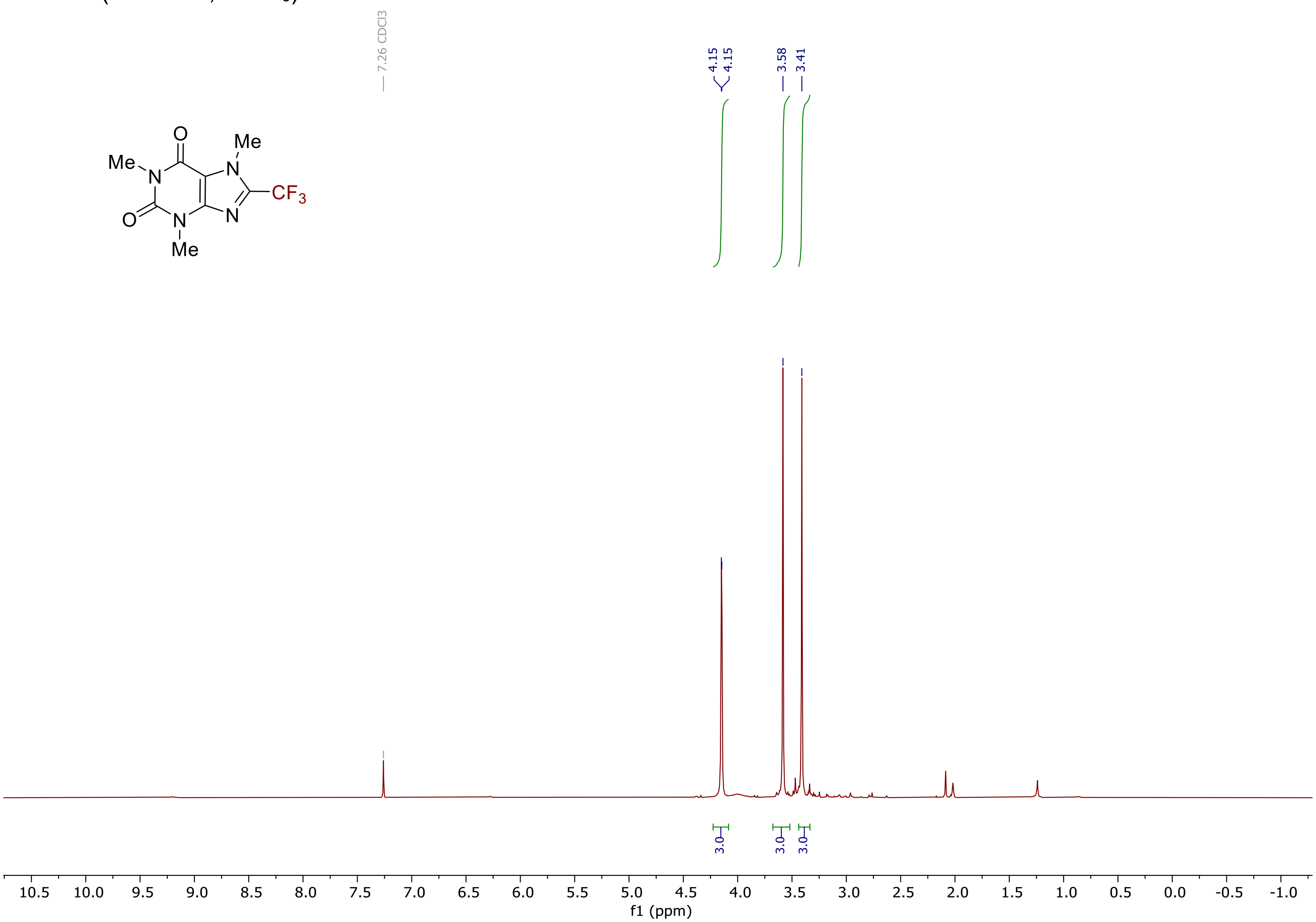

^{13}C NMR (75 MHz, $CDCl_3$)

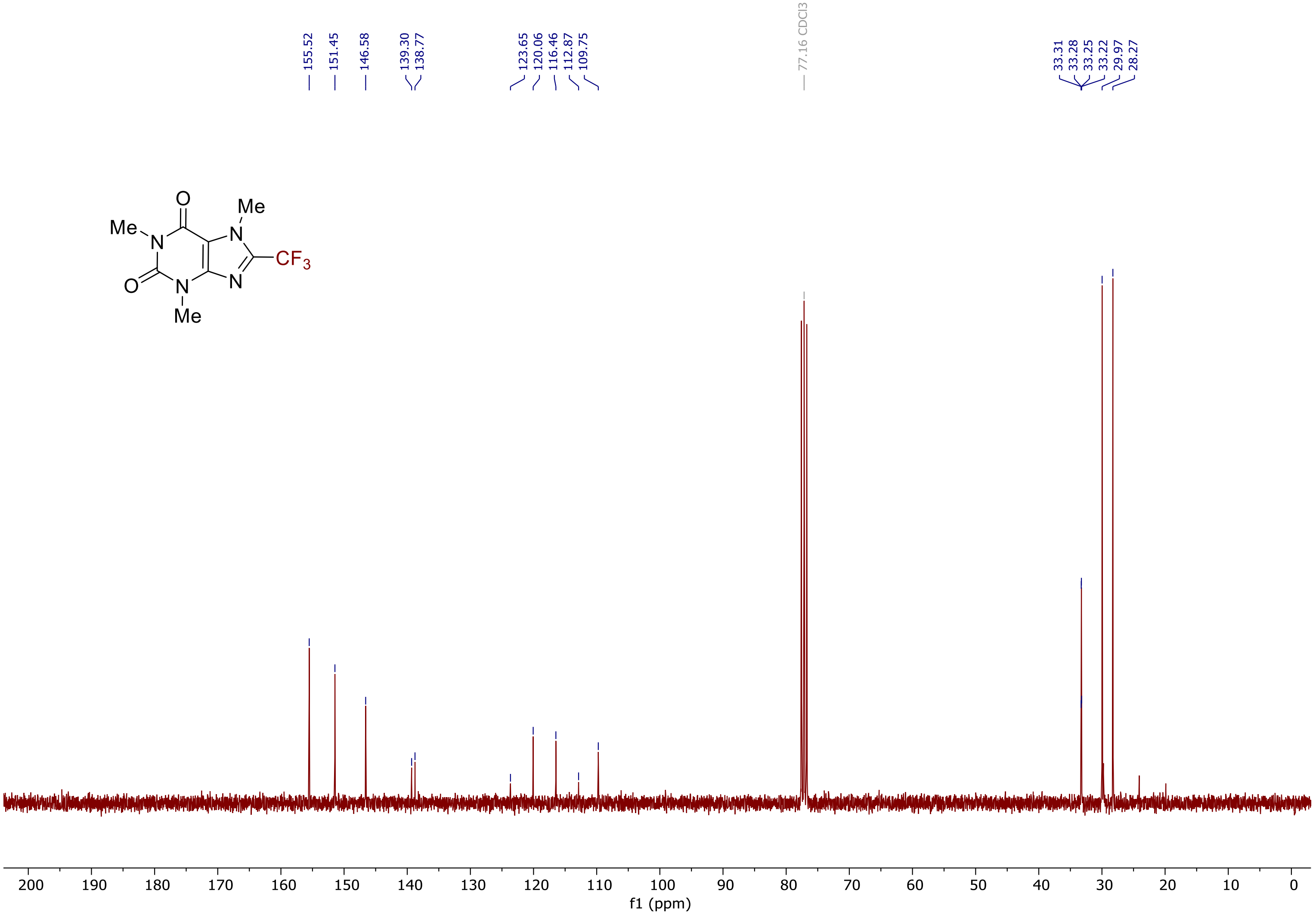

^{19}F NMR (282 MHz, $CDCl_3$)

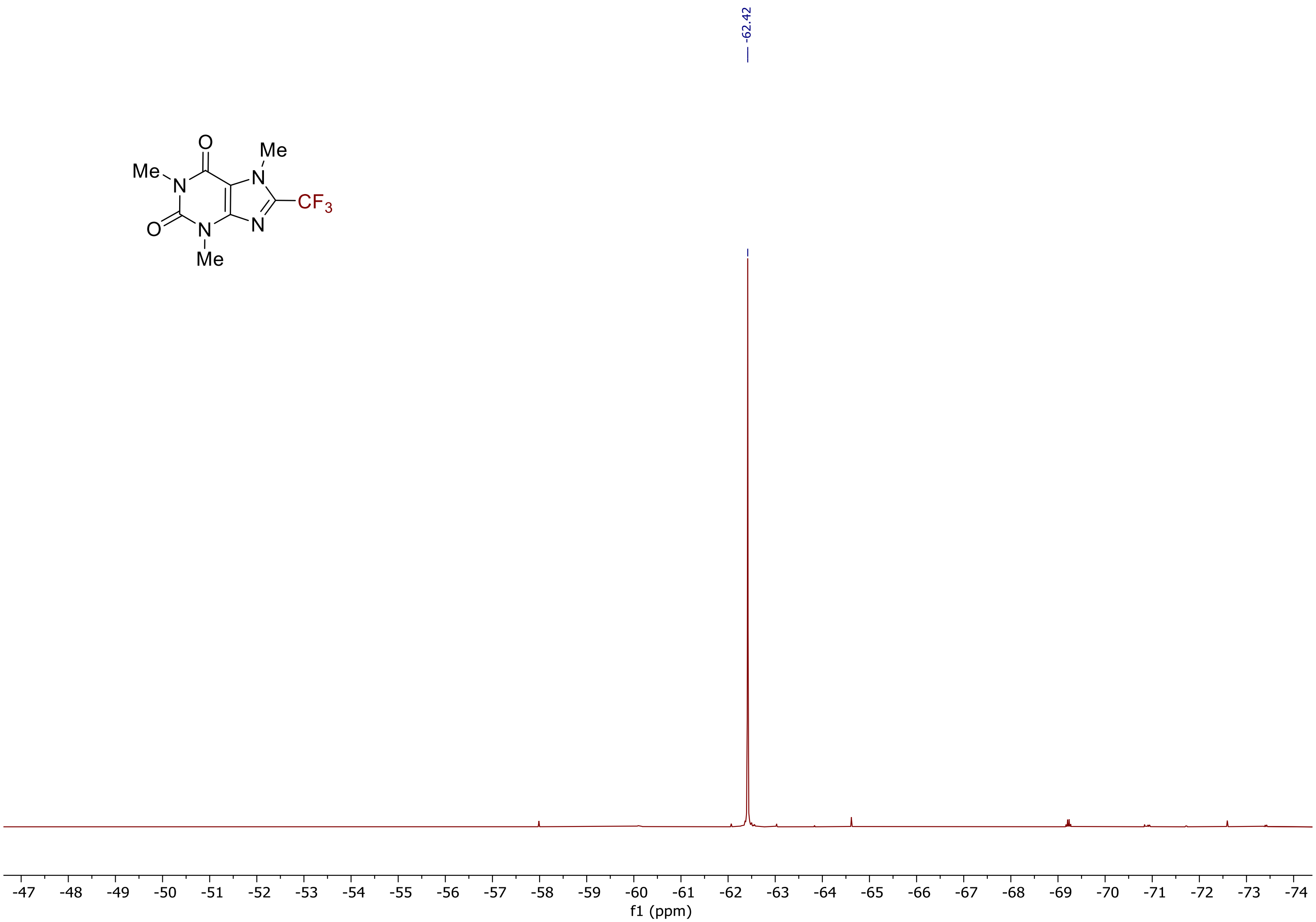

1,3,7-Trimethyl-8-(perfluoroethyl)-3,7-dihydro-1*H*-purine-2,6-dione (4)

^{1}H NMR (300 MHz, $CDCl_3$)

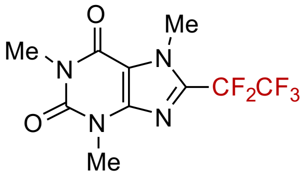

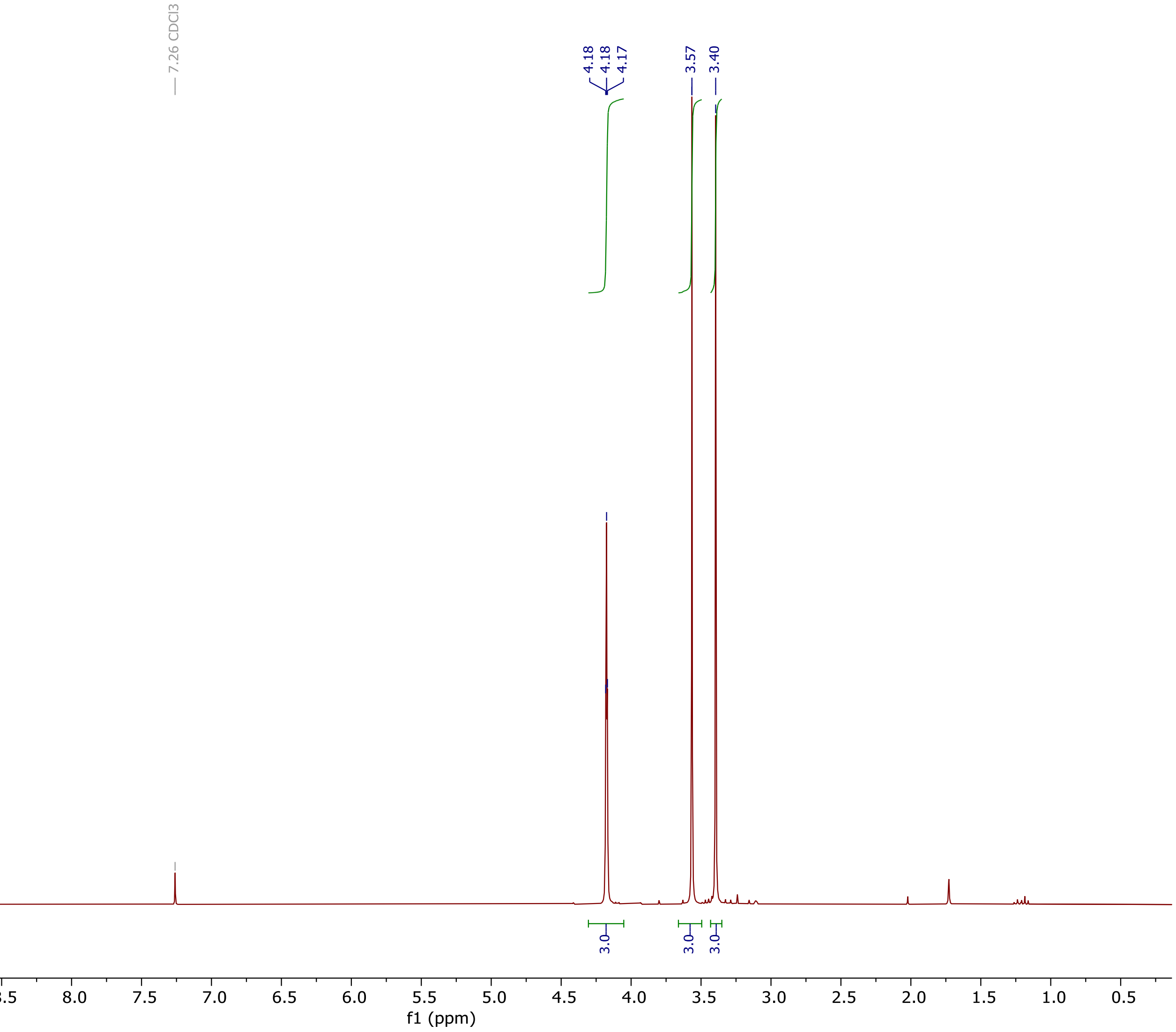

^{13}C NMR (75 MHz, $CDCl_3$)

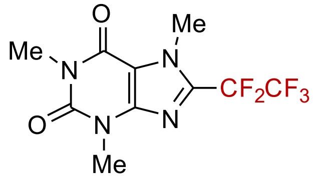

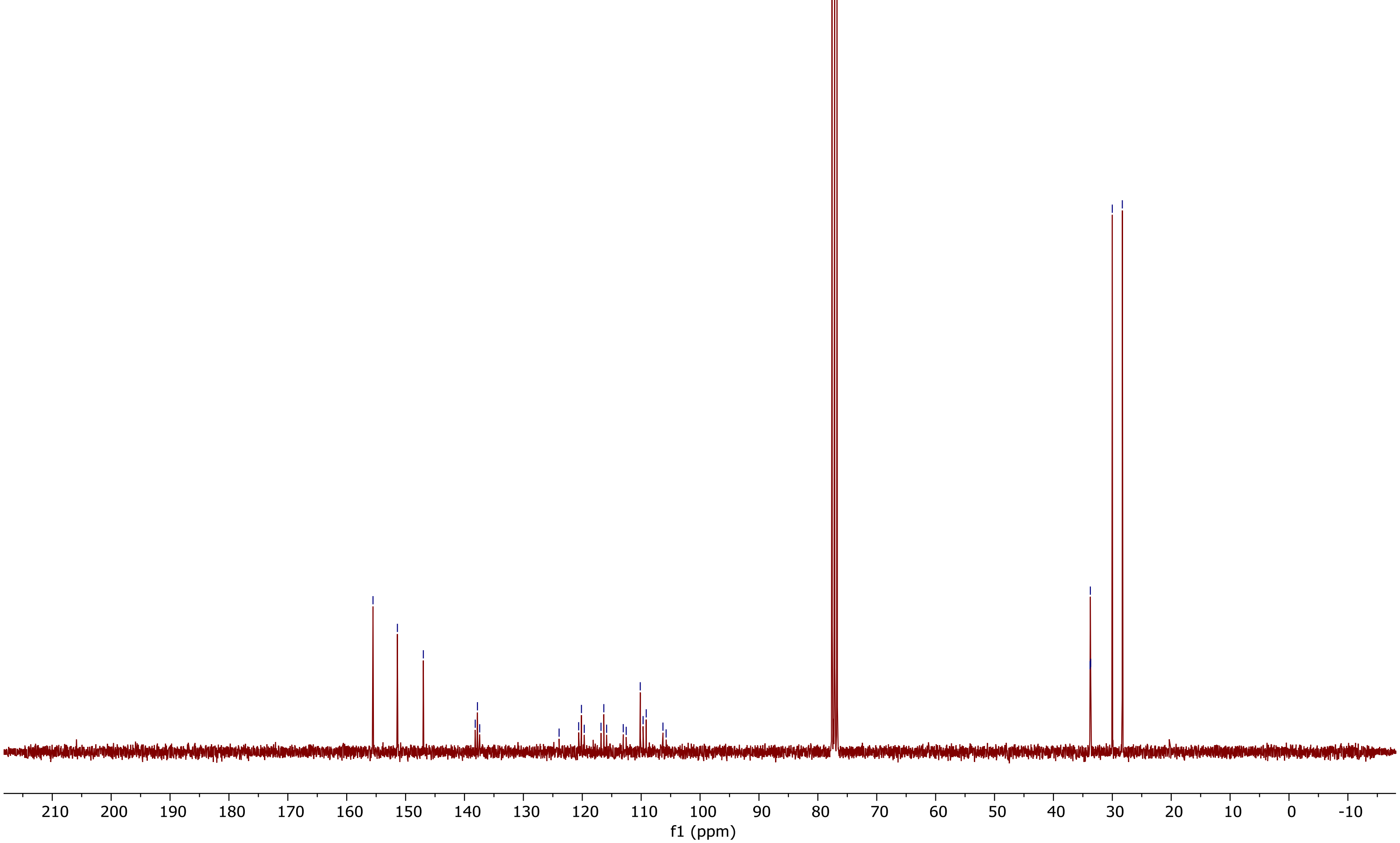

^{19}F NMR (282 MHz, $CDCl_3$)

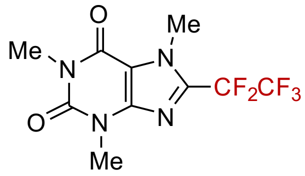

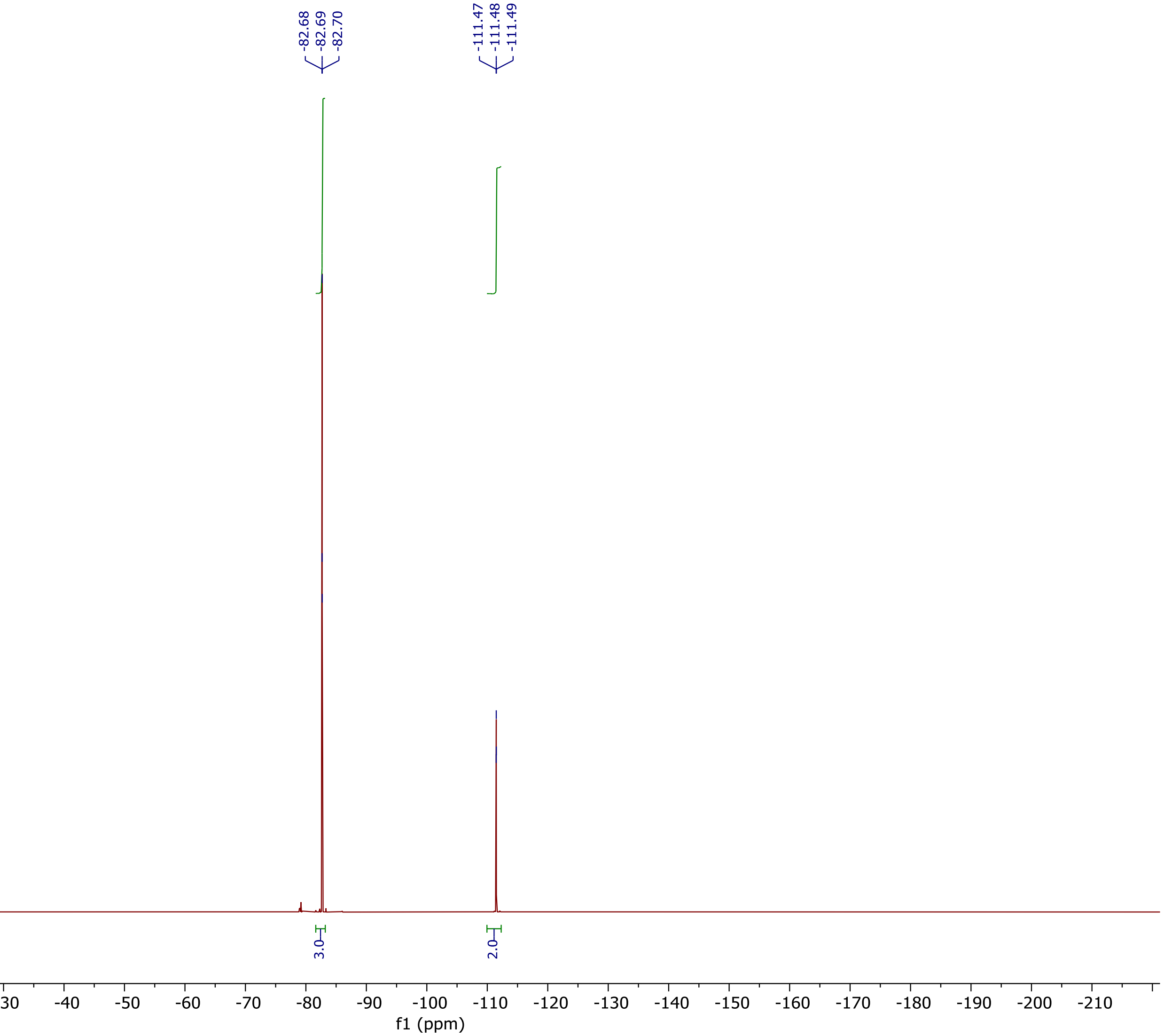

1,3,7-Trimethyl-8-(perfluoropropyl)-3,7-dihydro-1*H*-purine-2,6-dione (5)

^{1}H NMR (300 MHz, $CDCl_3$)

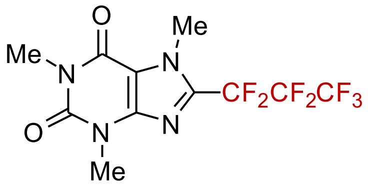

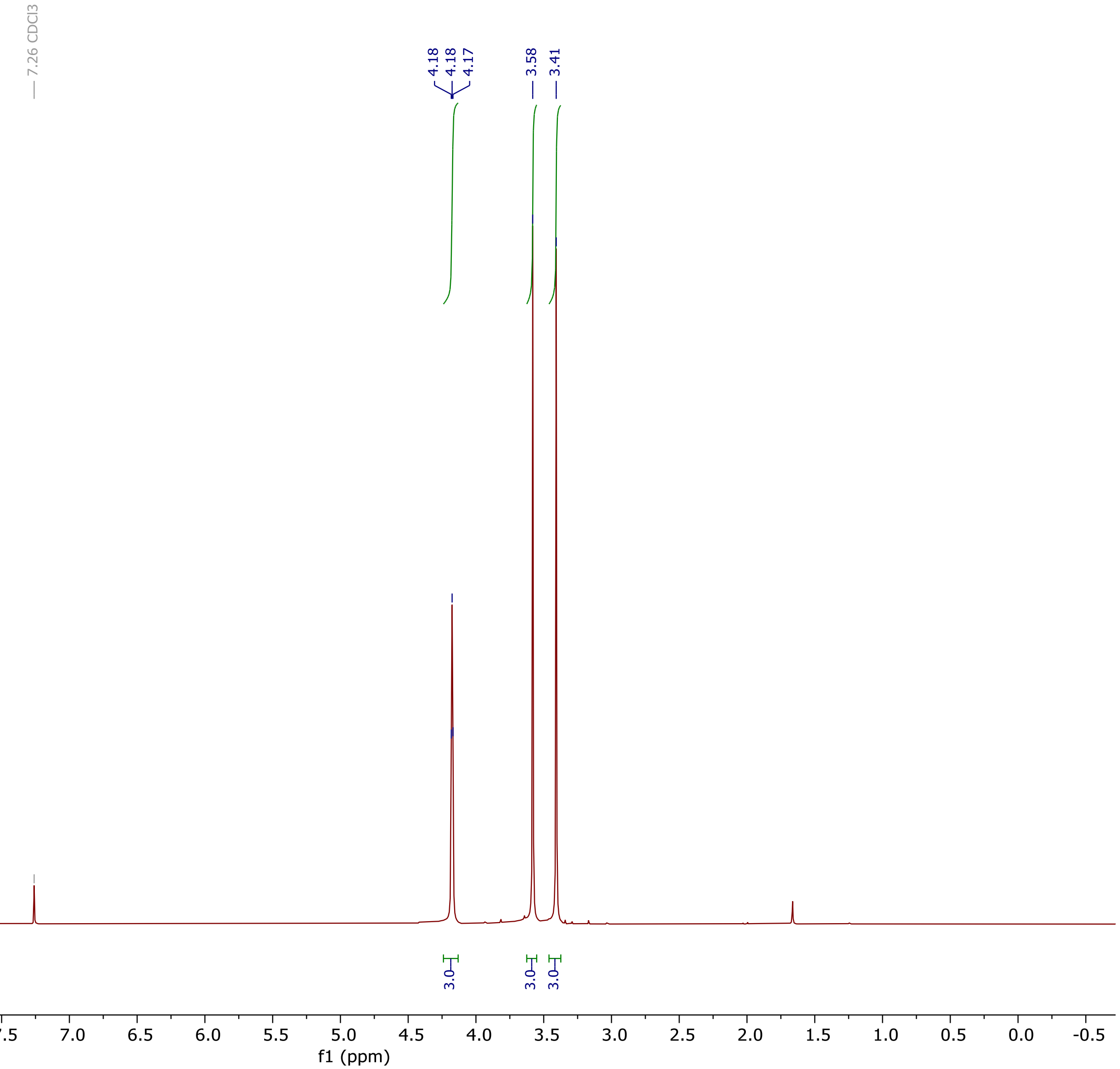

^{13}C NMR (75 MHz, $CDCl_3$)

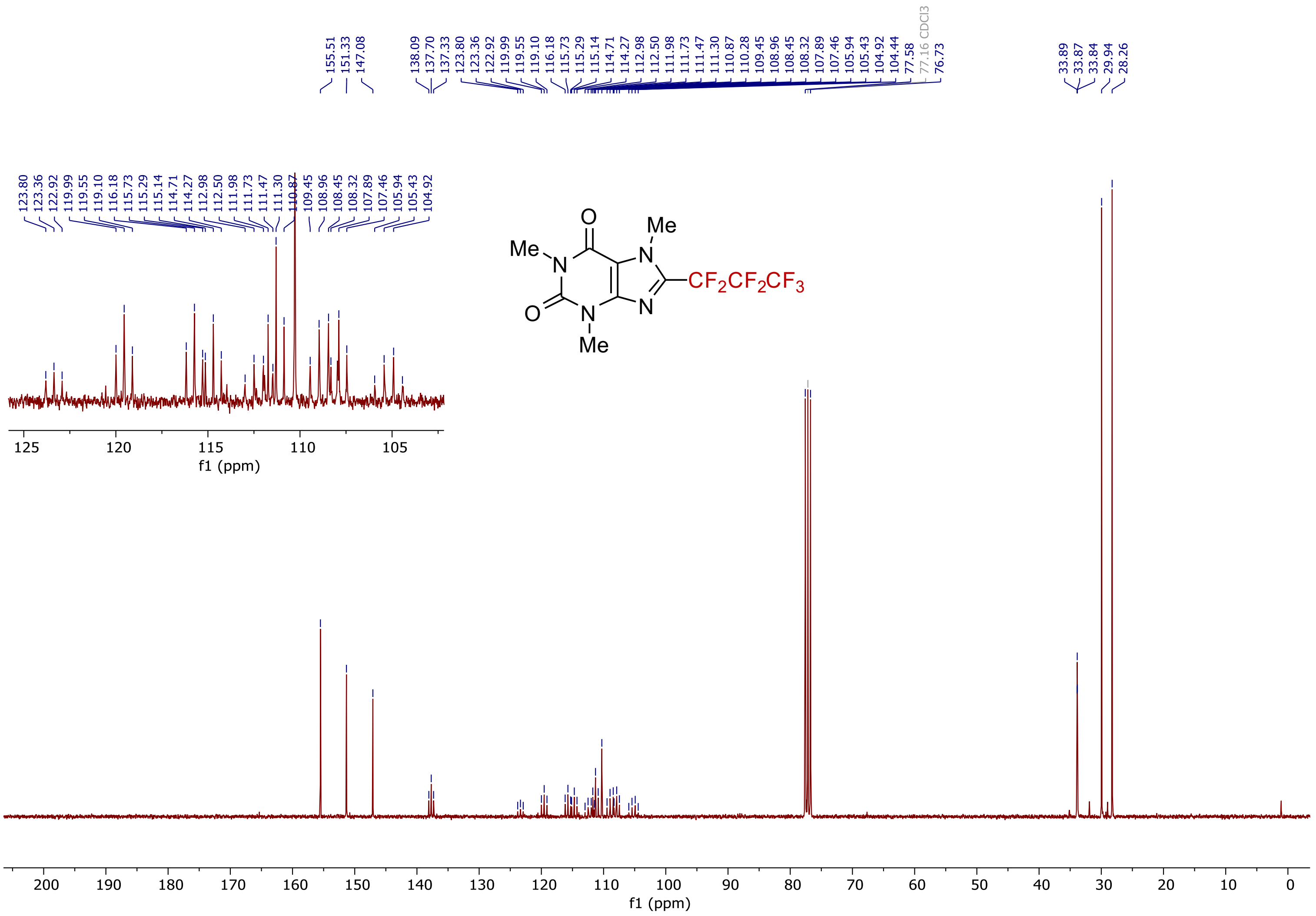

^{19}F NMR (282 MHz, $CDCl_3$)

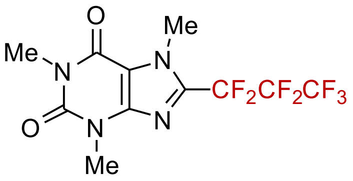

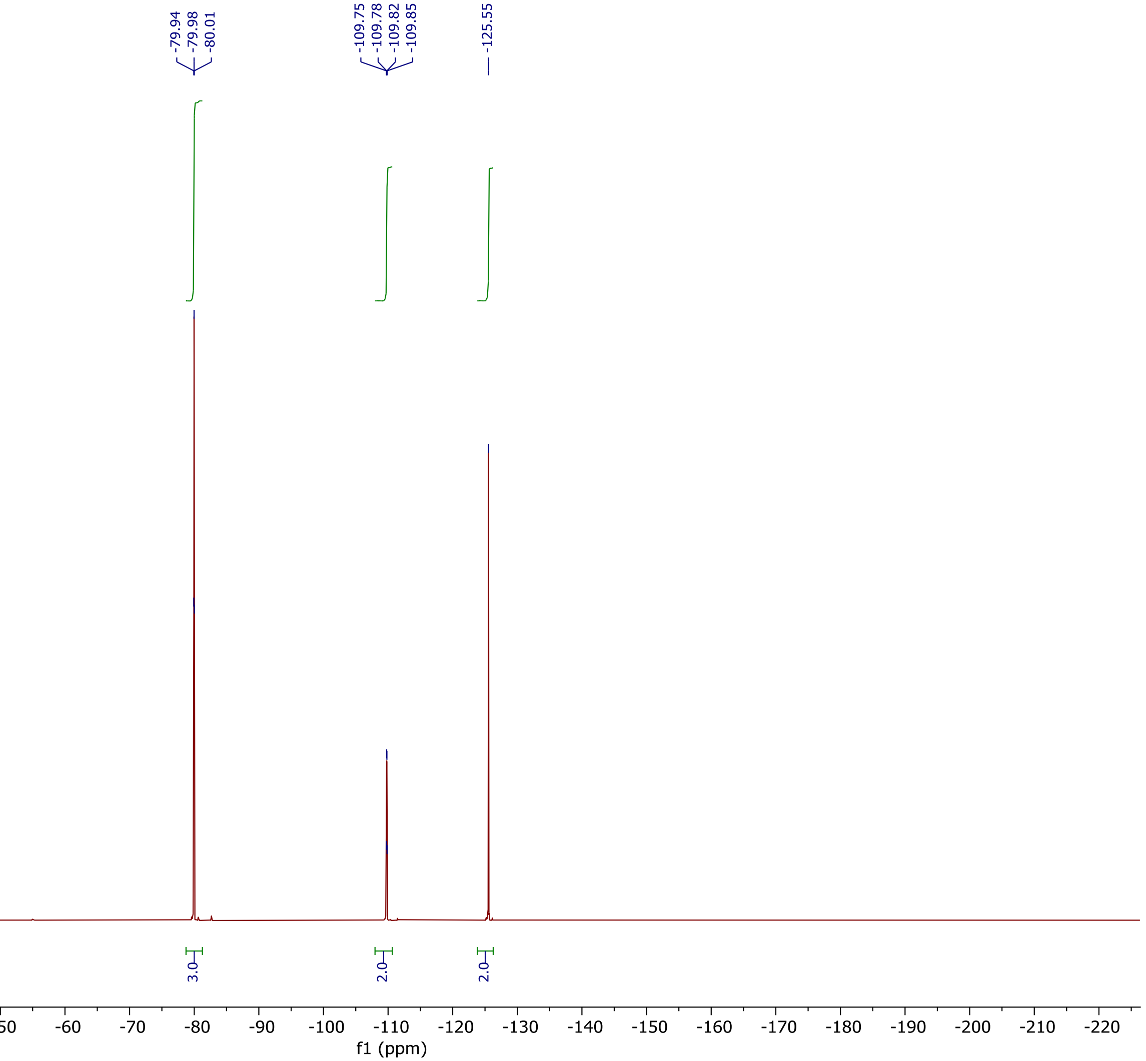

1,3,7-Trimethyl-8-(1,1,2,2-tetrafluoroethyl)-3,7-dihydro-1*H*-purine-2,6-dione (6)

^{1}H NMR (300 MHz, $CDCl_3$)

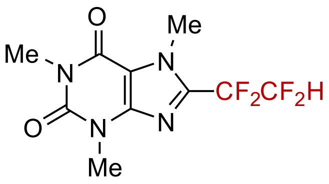

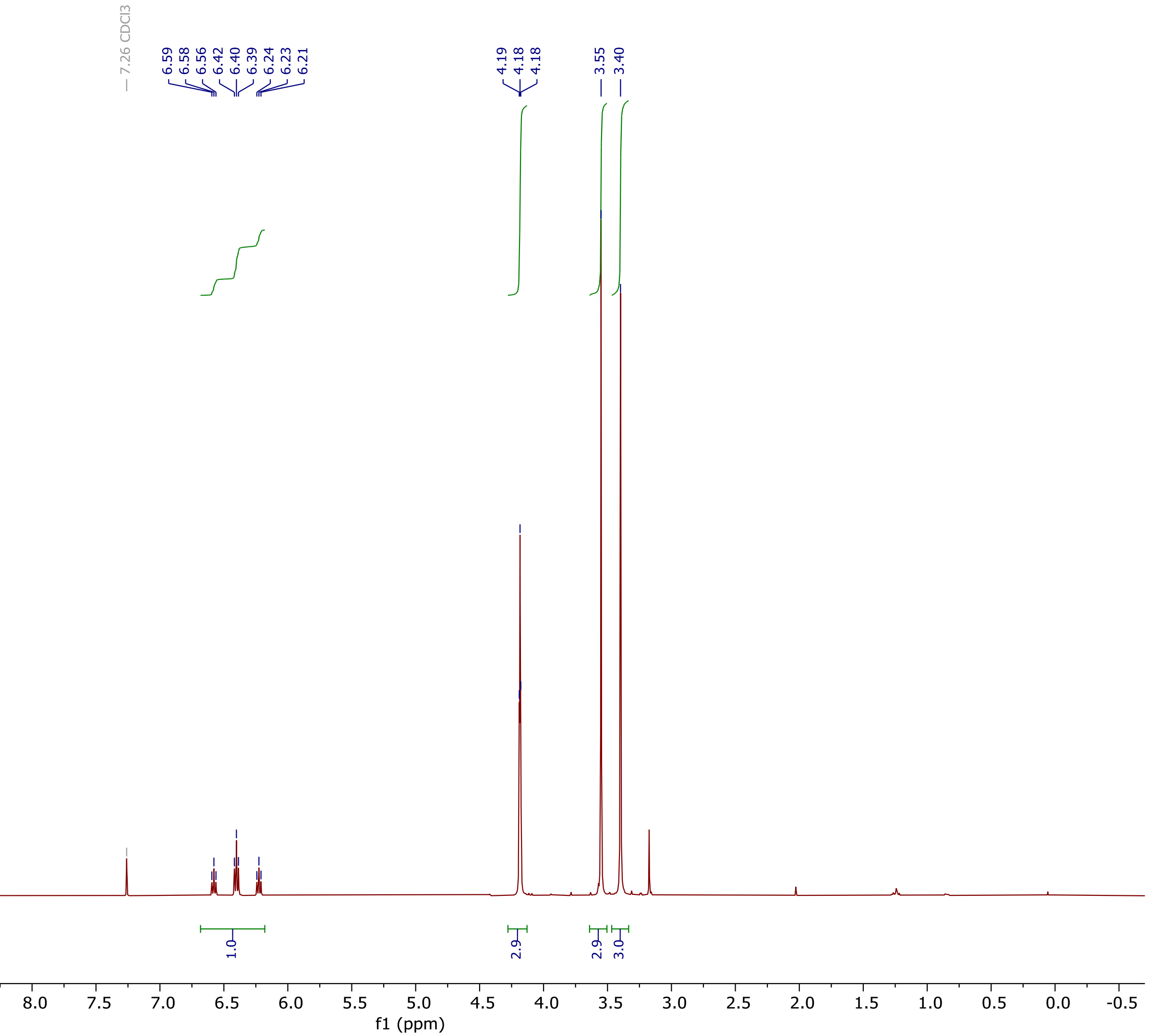

^{13}C NMR (75 MHz, $CDCl_3$)

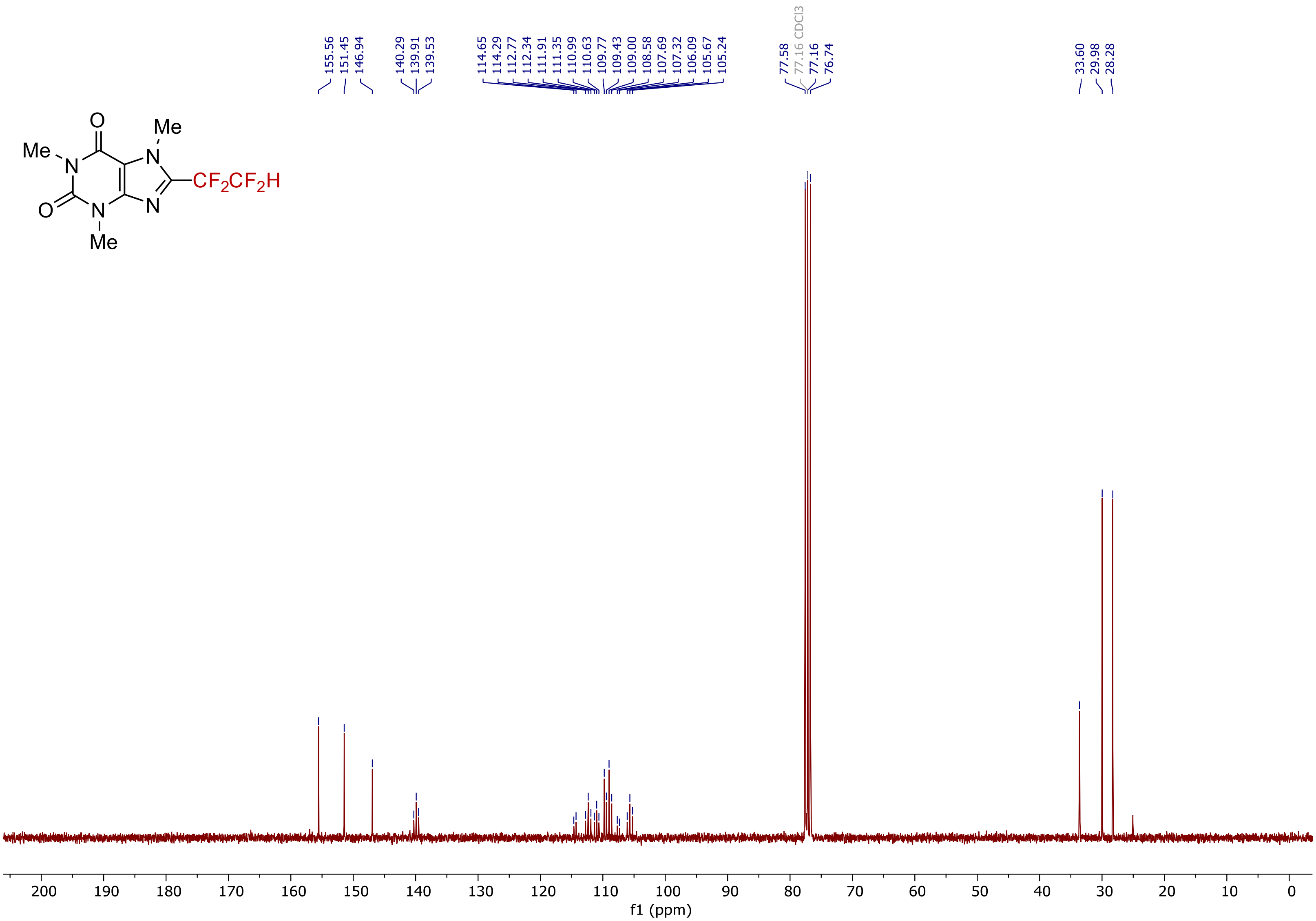

^{19}F NMR (282 MHz, $CDCl_3$)

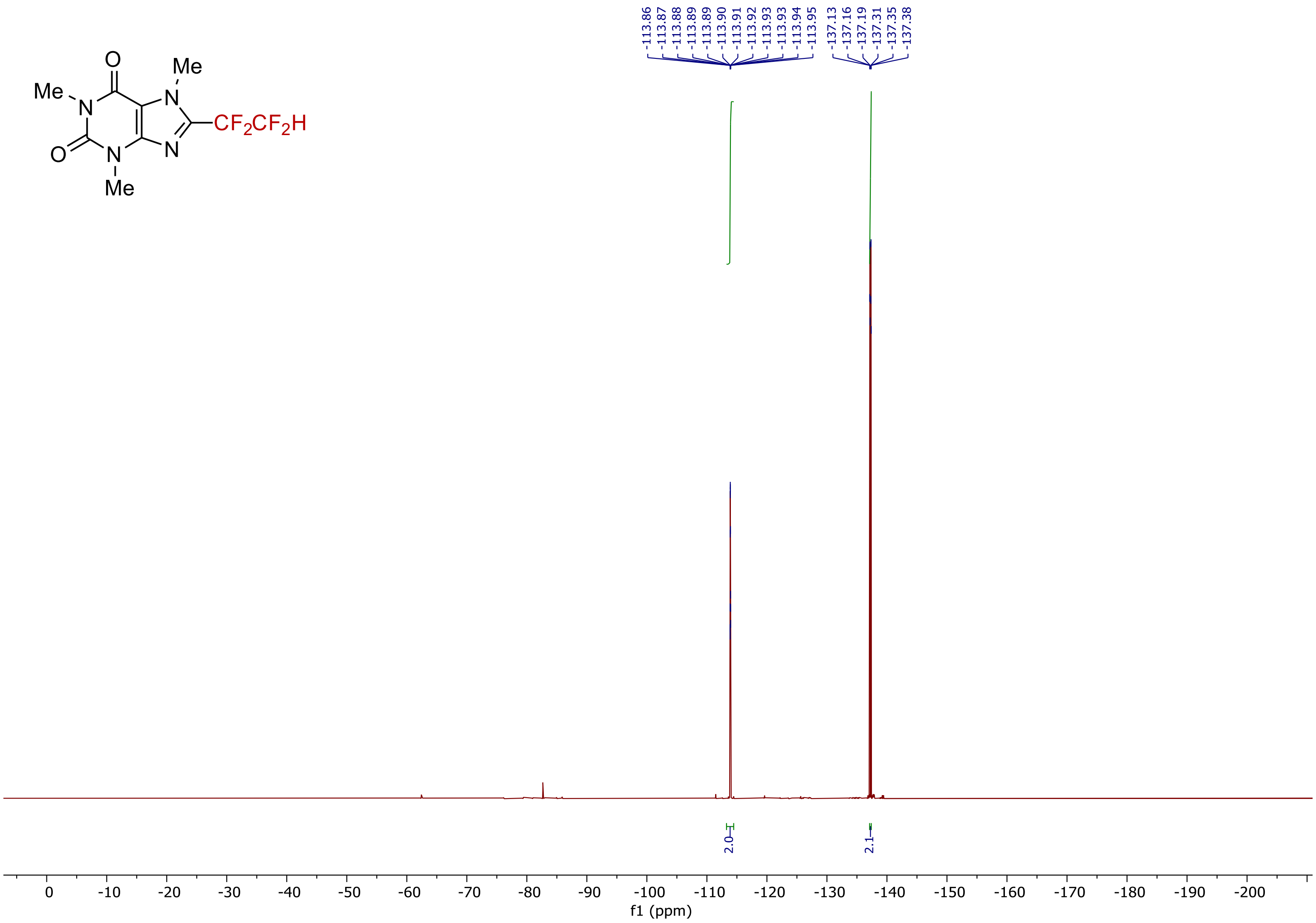

8-(1,1-Difluoroethyl)-1,3,7-trimethyl-3,7-dihydro-1*H*-purine-2,6-dione (7)

^{1}H NMR (300 MHz, $CDCl_3$)

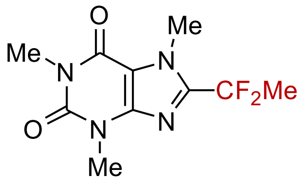

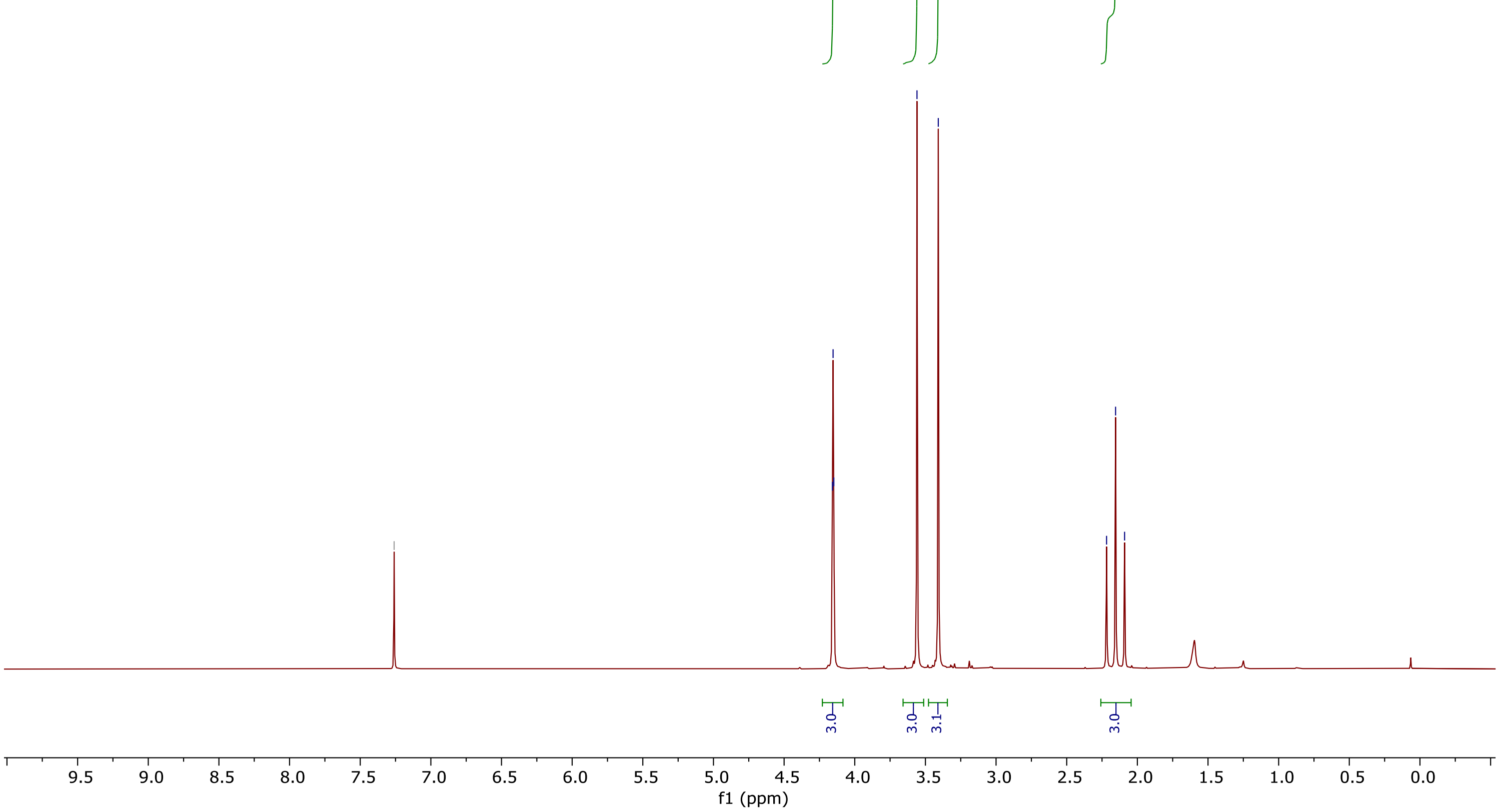

^{13}C NMR (75 MHz, $CDCl_3$)

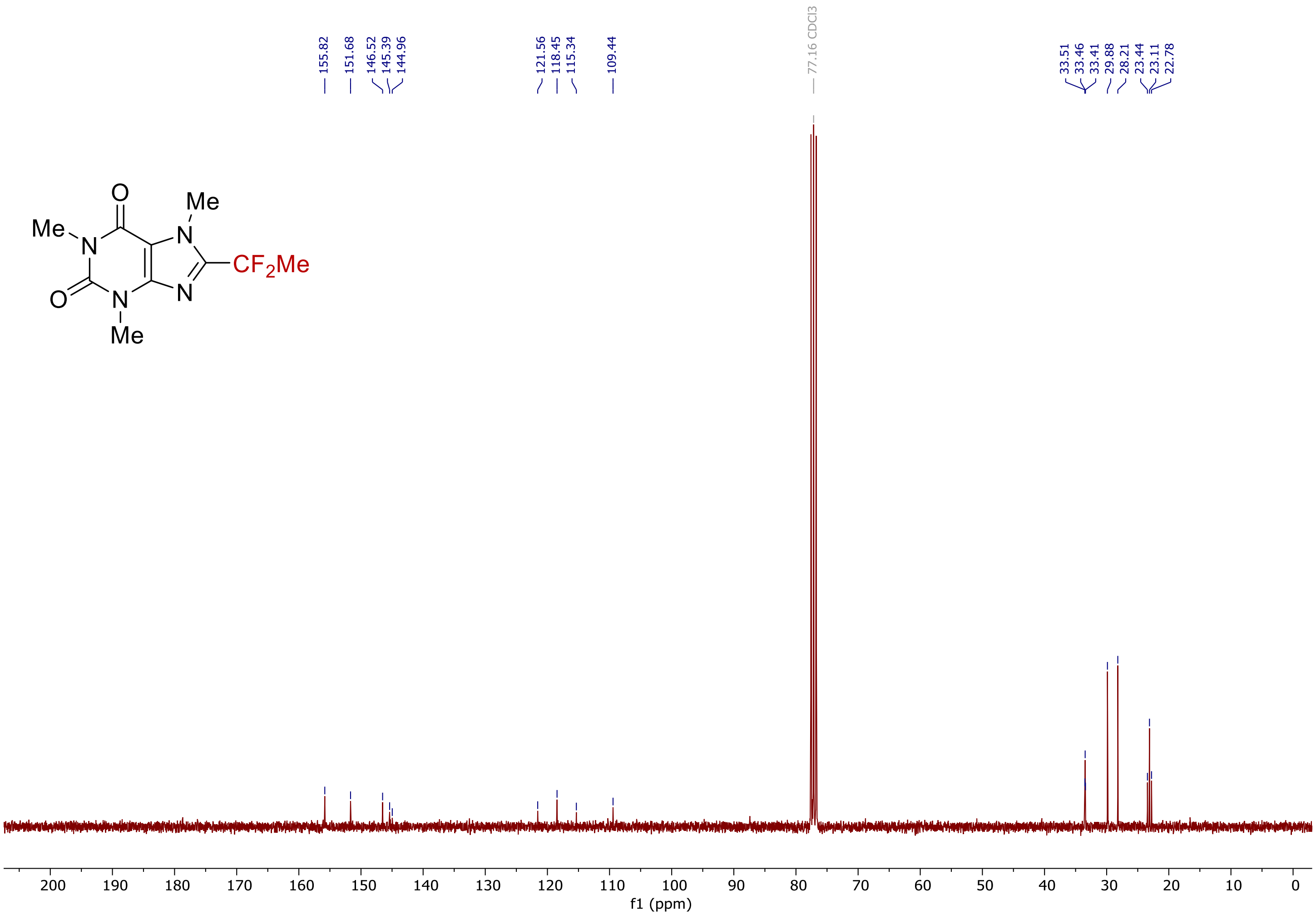

^{19}F NMR (282 MHz, $CDCl_3$)

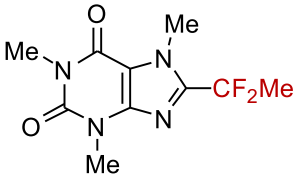

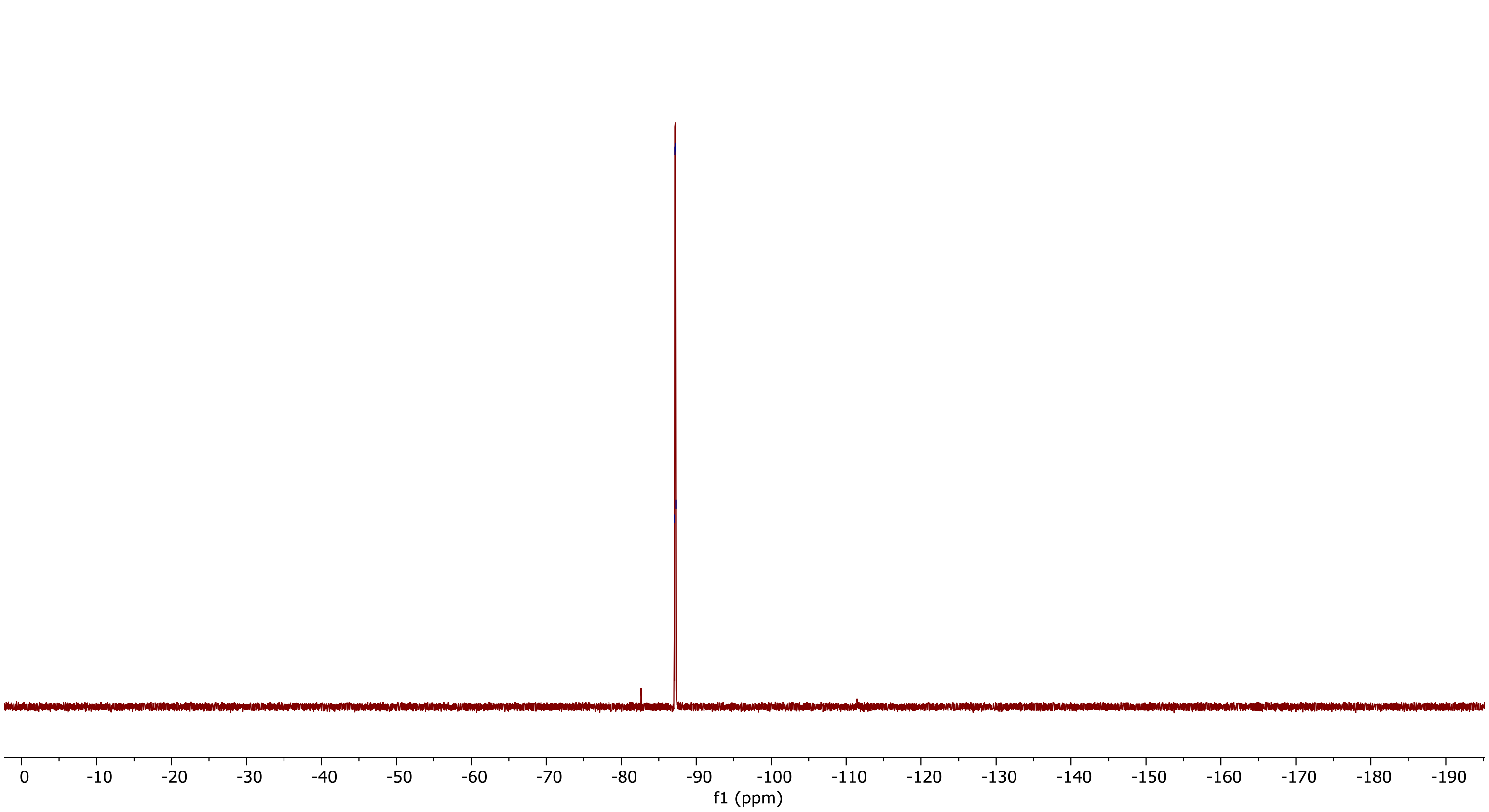

1,3-Dimethyl-5-(trifluoromethyl)pyrimidine-2,4(1*H*,3*H*)-dione (10)

^{1}H NMR (300 MHz, $CDCl_3$)

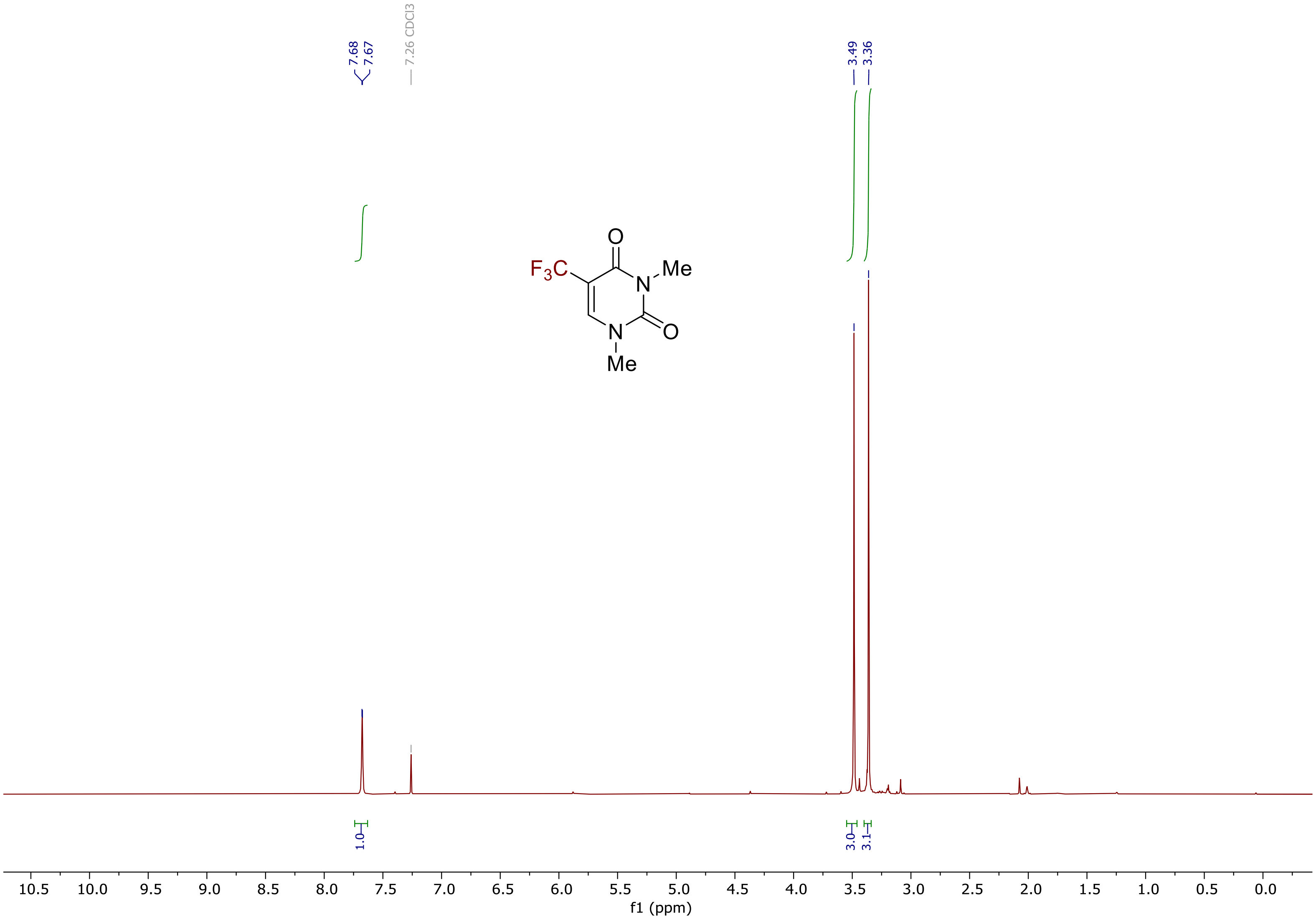

^{13}C NMR (75 MHz, $CDCl_3$)

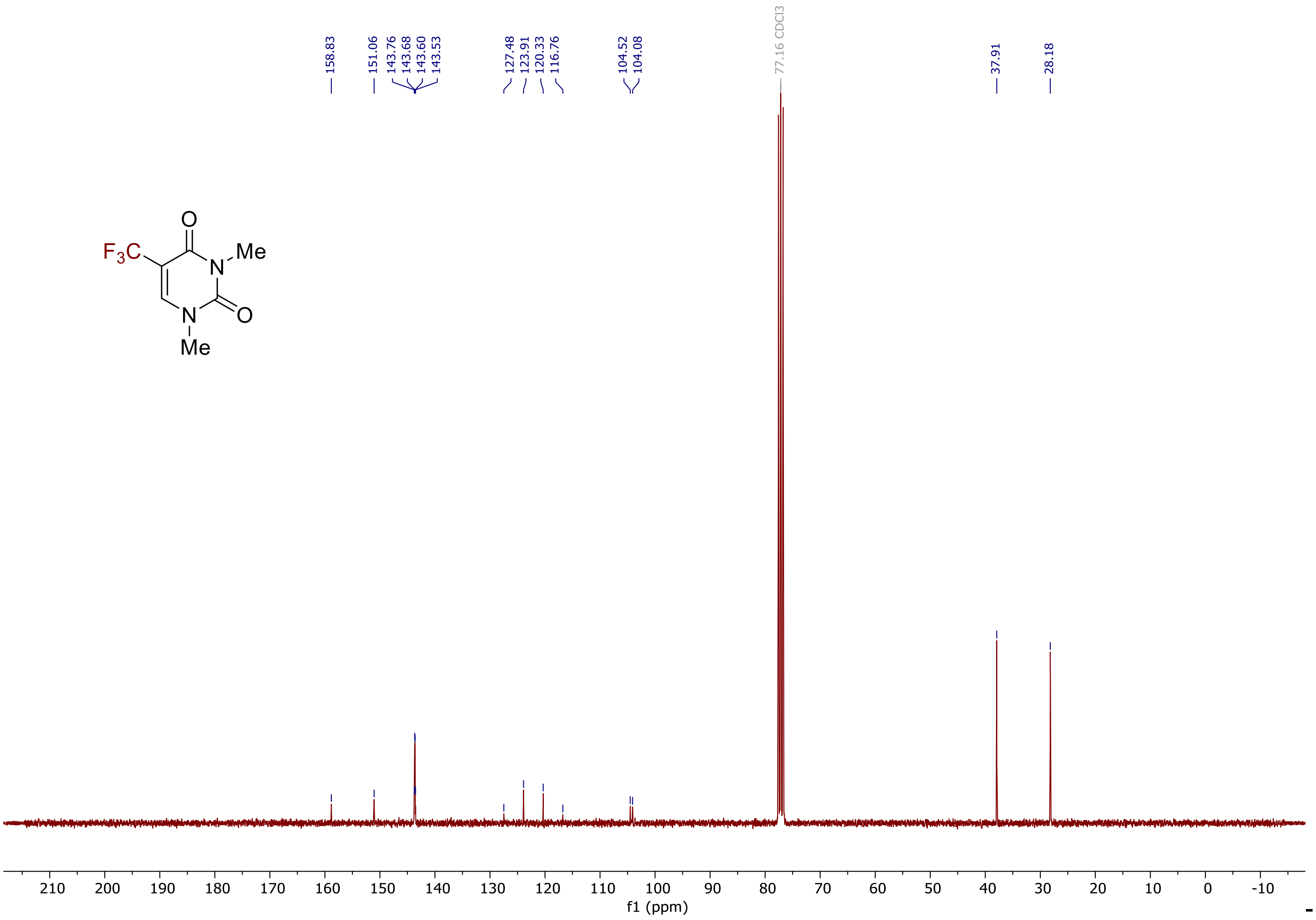

^{19}F NMR (282 MHz, $CDCl_3$)

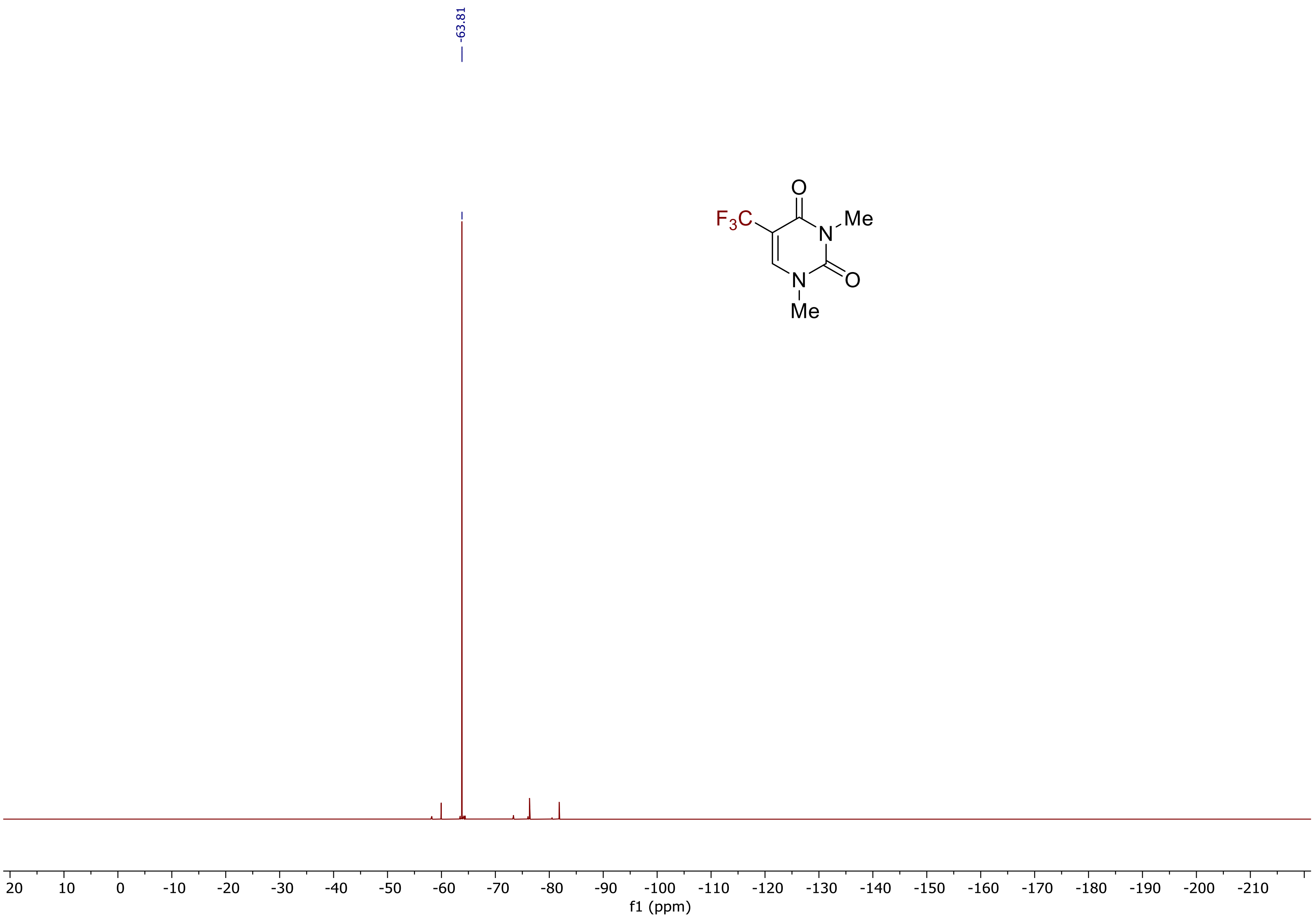

2,4-Dimethyl-6-(trifluoromethyl)-1,2,4-triazine-3,5(2*H*,4*H*)-dione (11)

^{1}H NMR (300 MHz, $CDCl_3$)

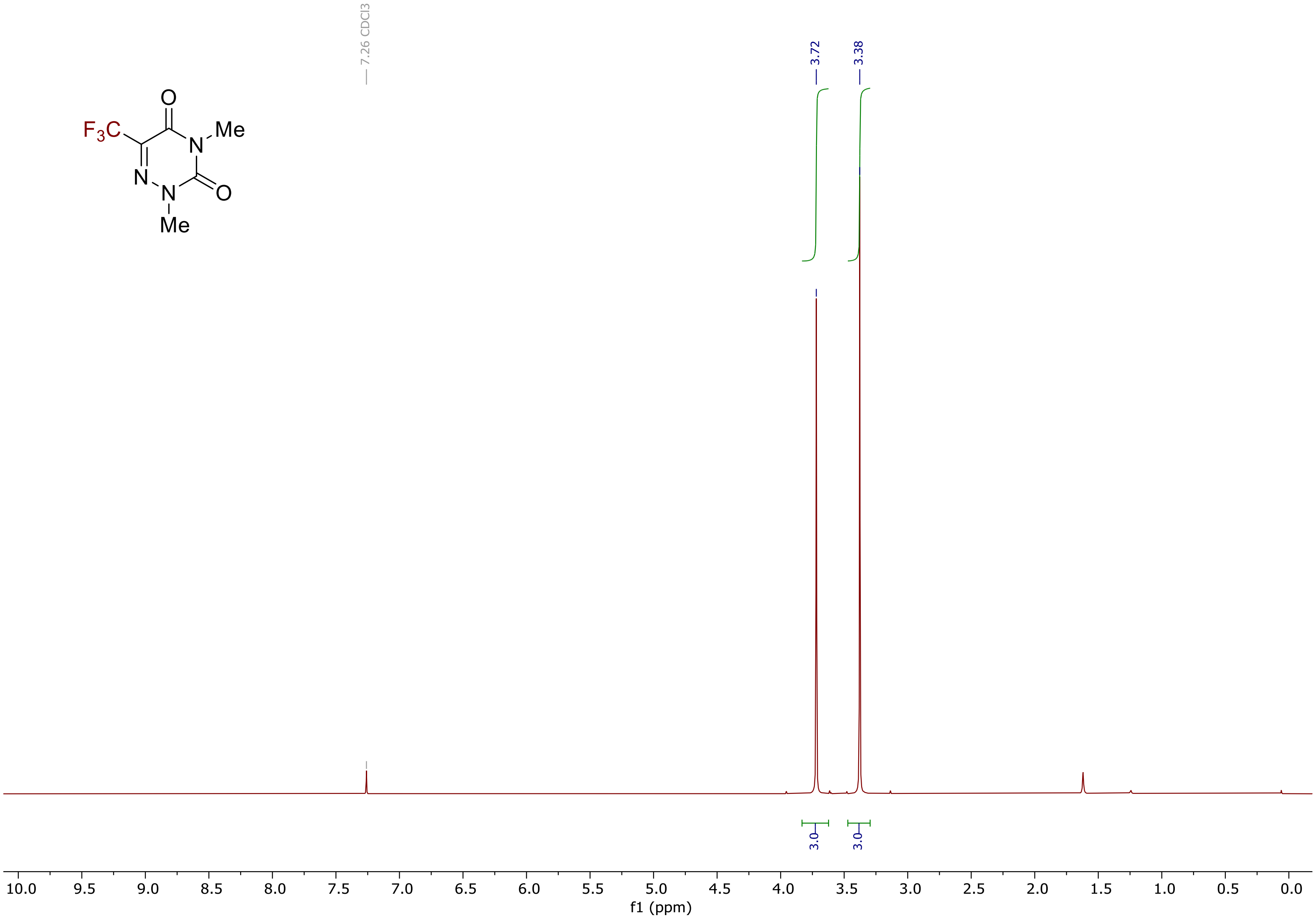

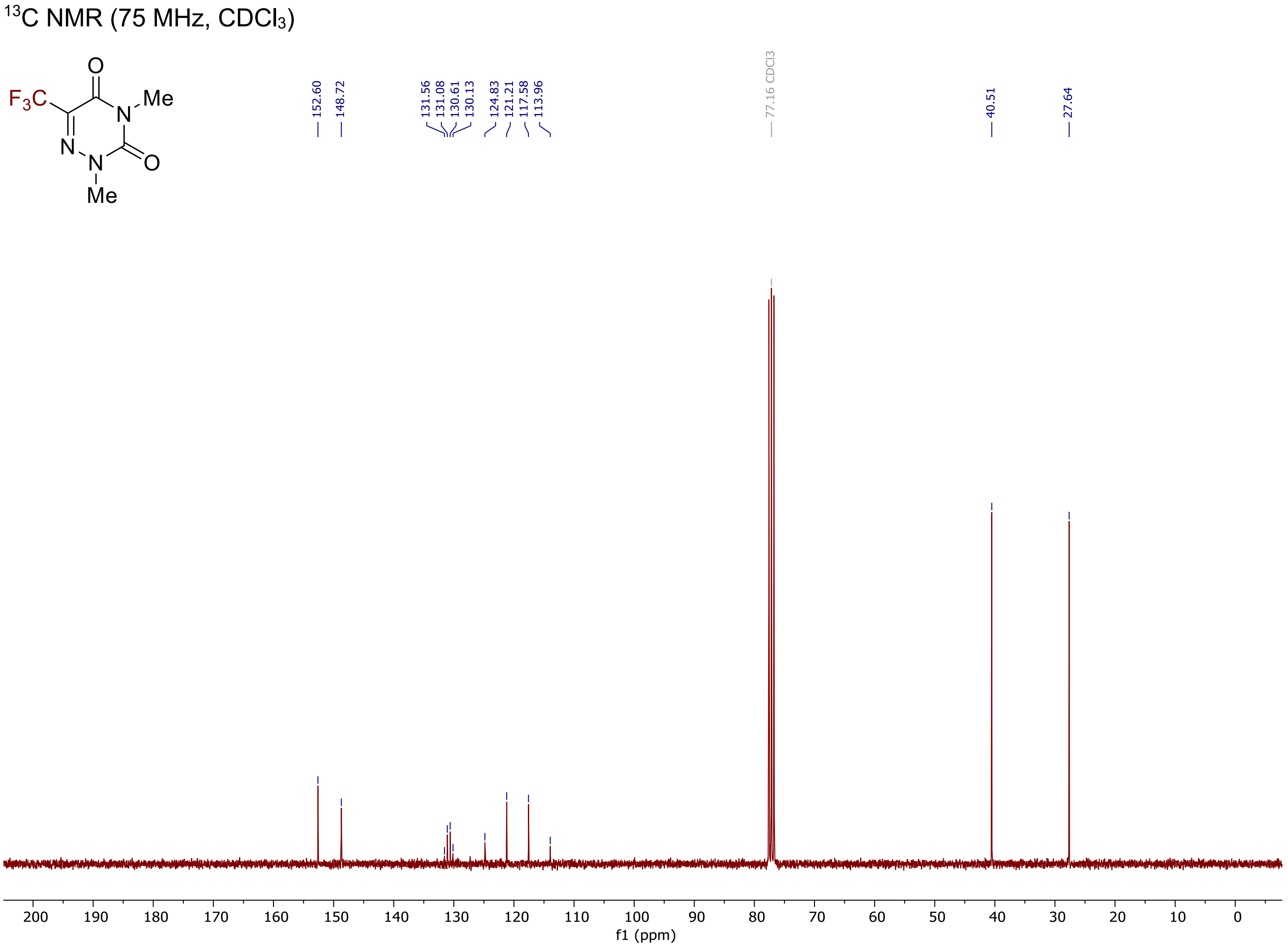
13C NMR (75 MHz, CDCl3)
F3C
O
N
Me
N
N
O
Me
152.60
148.72
131.56
131.08
130.61
130.13
124.83
121.21
117.58
113.96
77.16 CDCl3
40.51
27.64
200
190
180
170
160
150
140
130
120
110
100
90
80
70
60
50
40
30
20
10
0
f1 (ppm)

^{19}F NMR (282 MHz, $CDCl_3$)

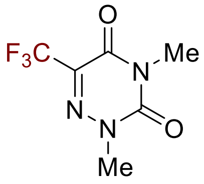

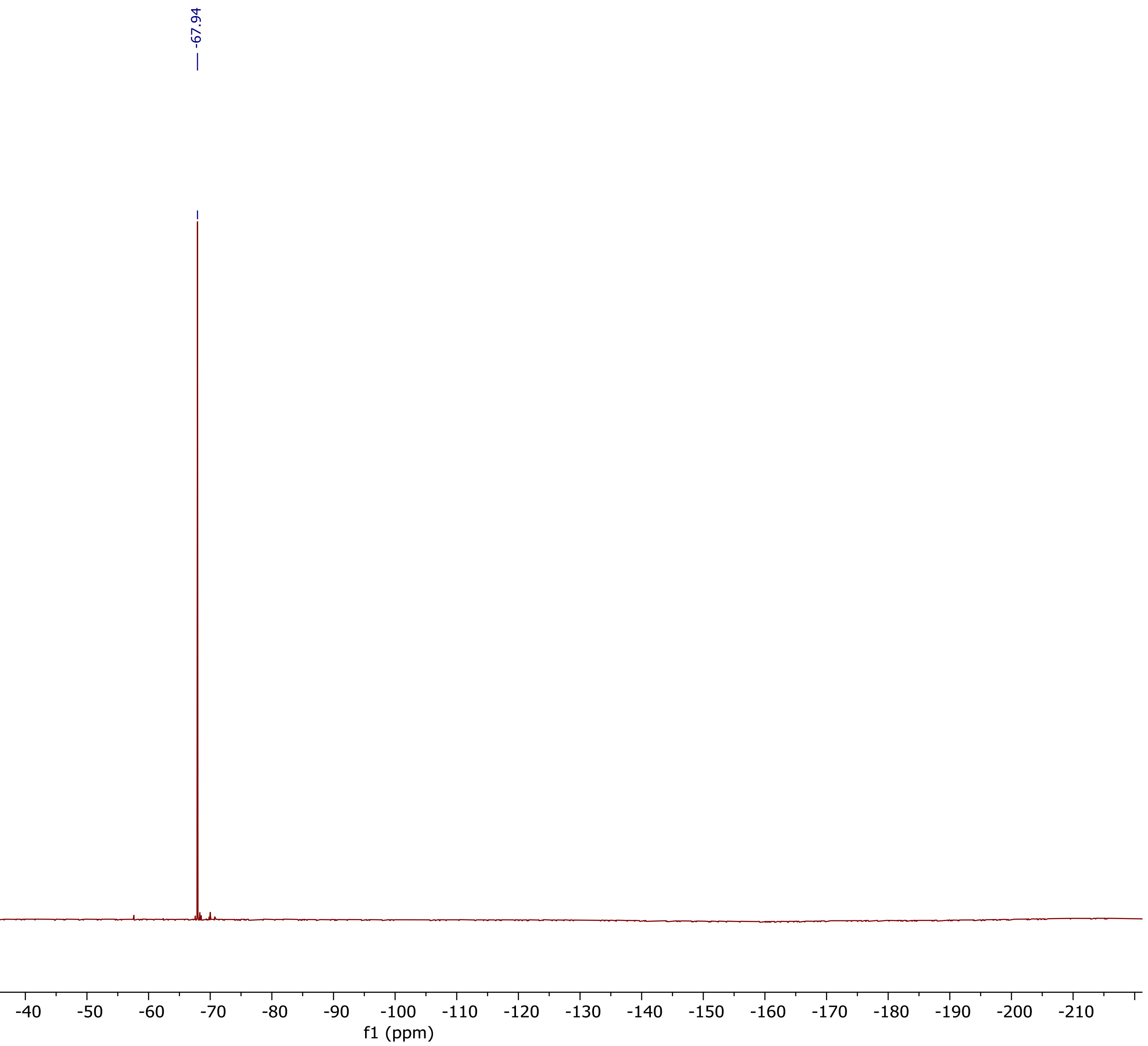

2,4-Dimethyl-6-(perfluoroethyl)-1,2,4-triazine-3,5(2*H*,4*H*)-dione (12)

^{1}H NMR (300 MHz, $CDCl_3$)

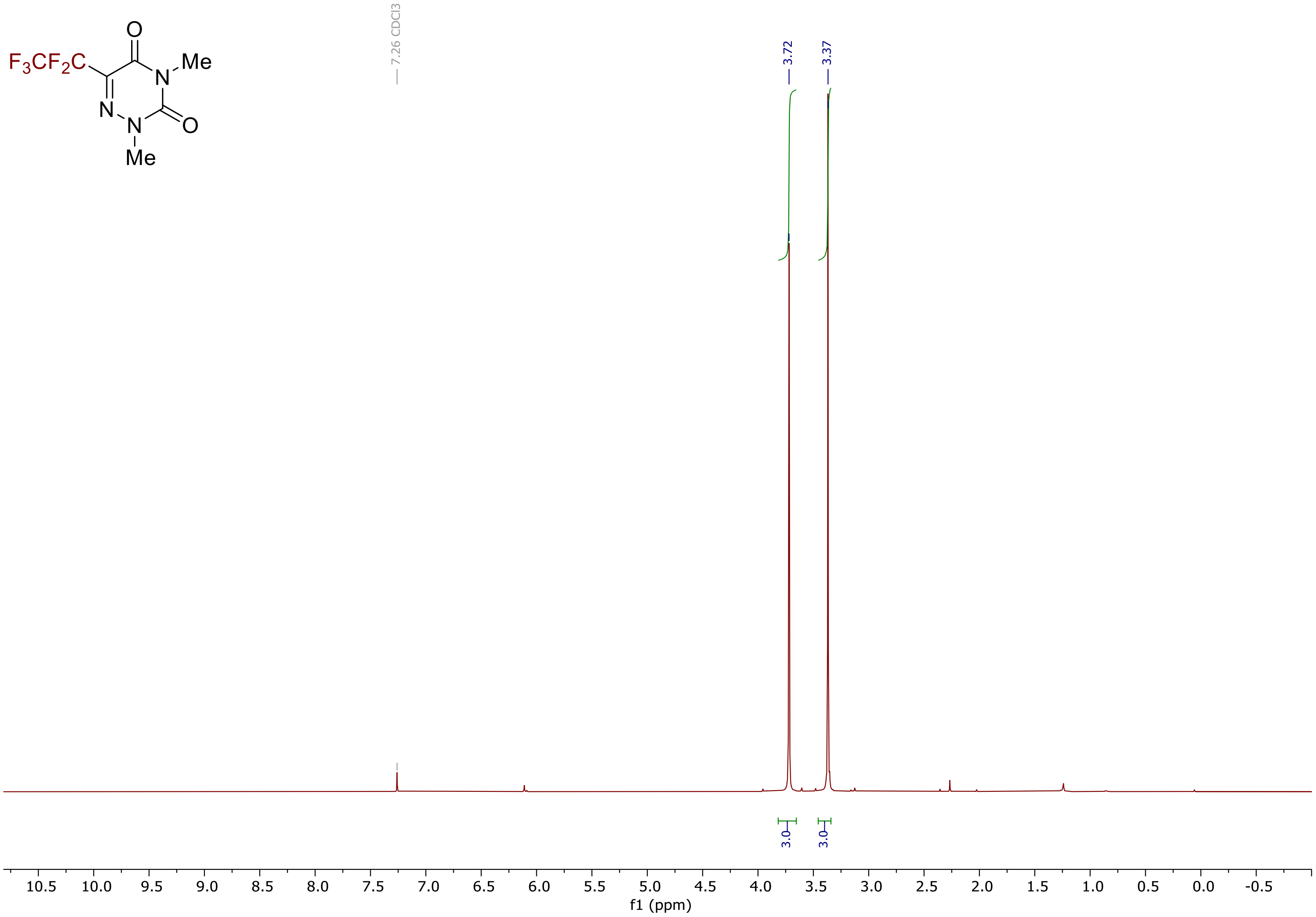

^{13}C NMR (75 MHz, $CDCl_3$)

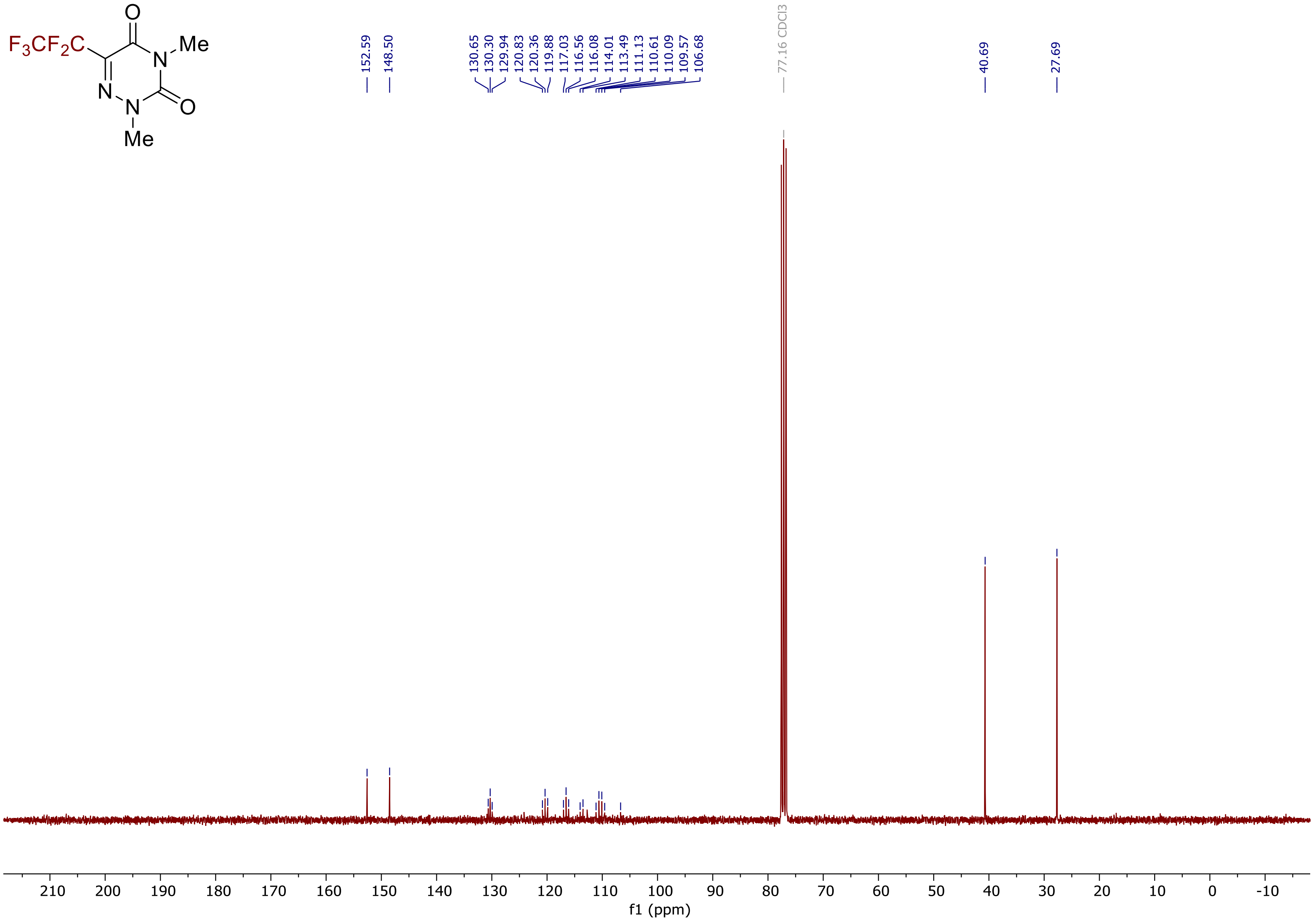

^{19}F NMR (282 MHz, $CDCl_3$)

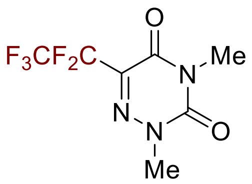

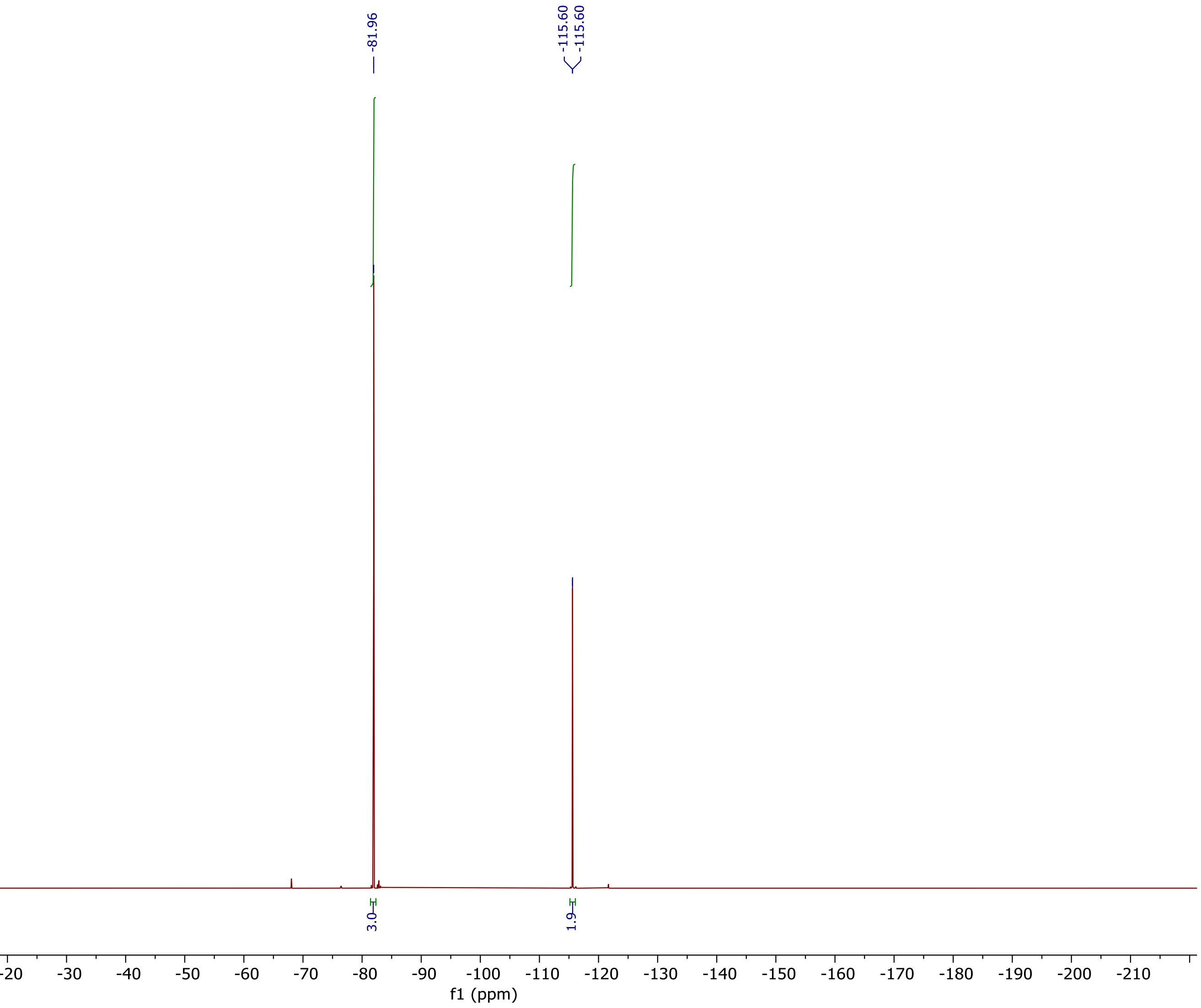

2,4-Dimethyl-6-(perfluoropropyl)-1,2,4-triazine-3,5(2*H*,4*H*)-dione (13)

^{1}H NMR (300 MHz, $CDCl_3$)

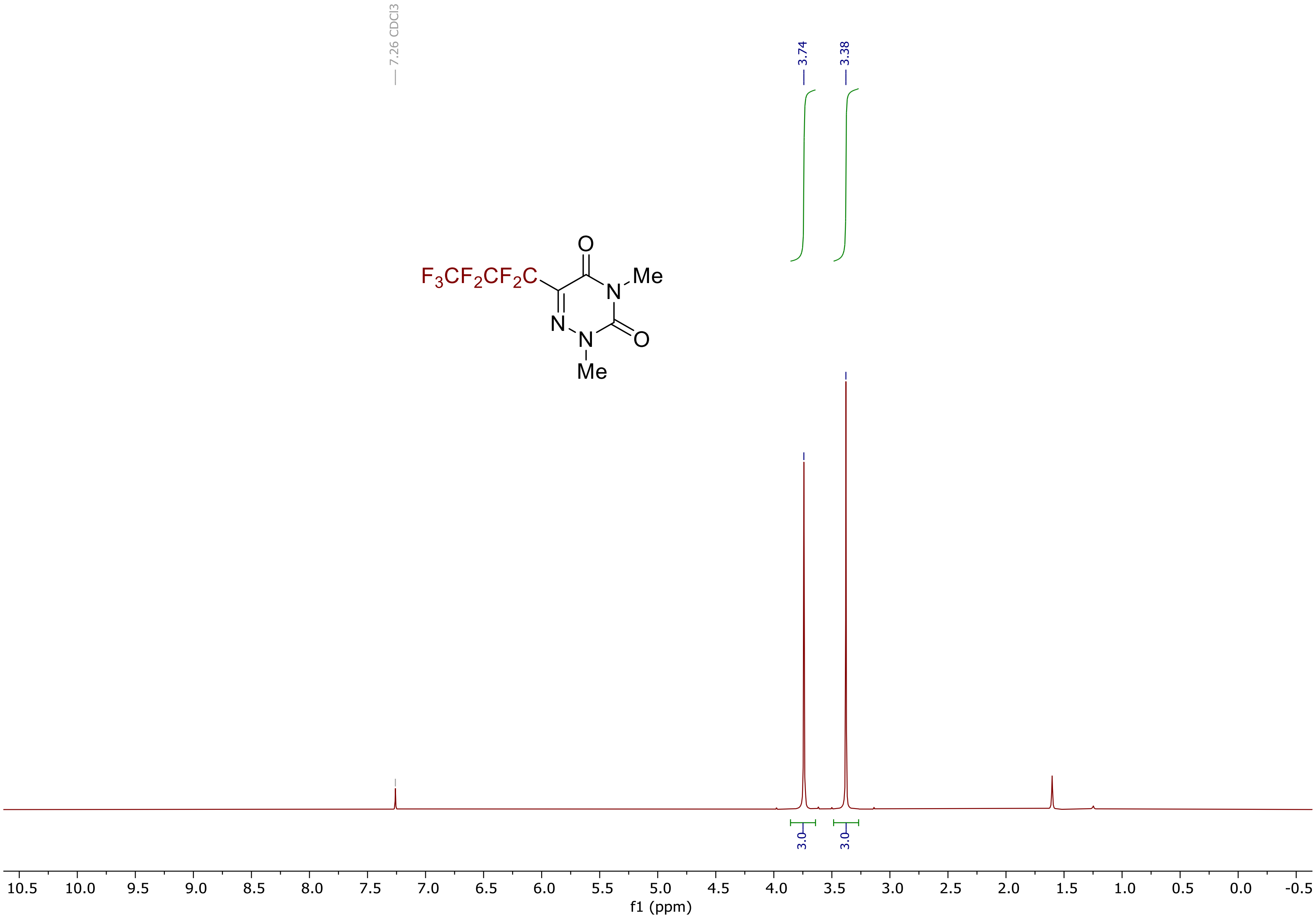

^{13}C NMR (75 MHz, $CDCl_3$)

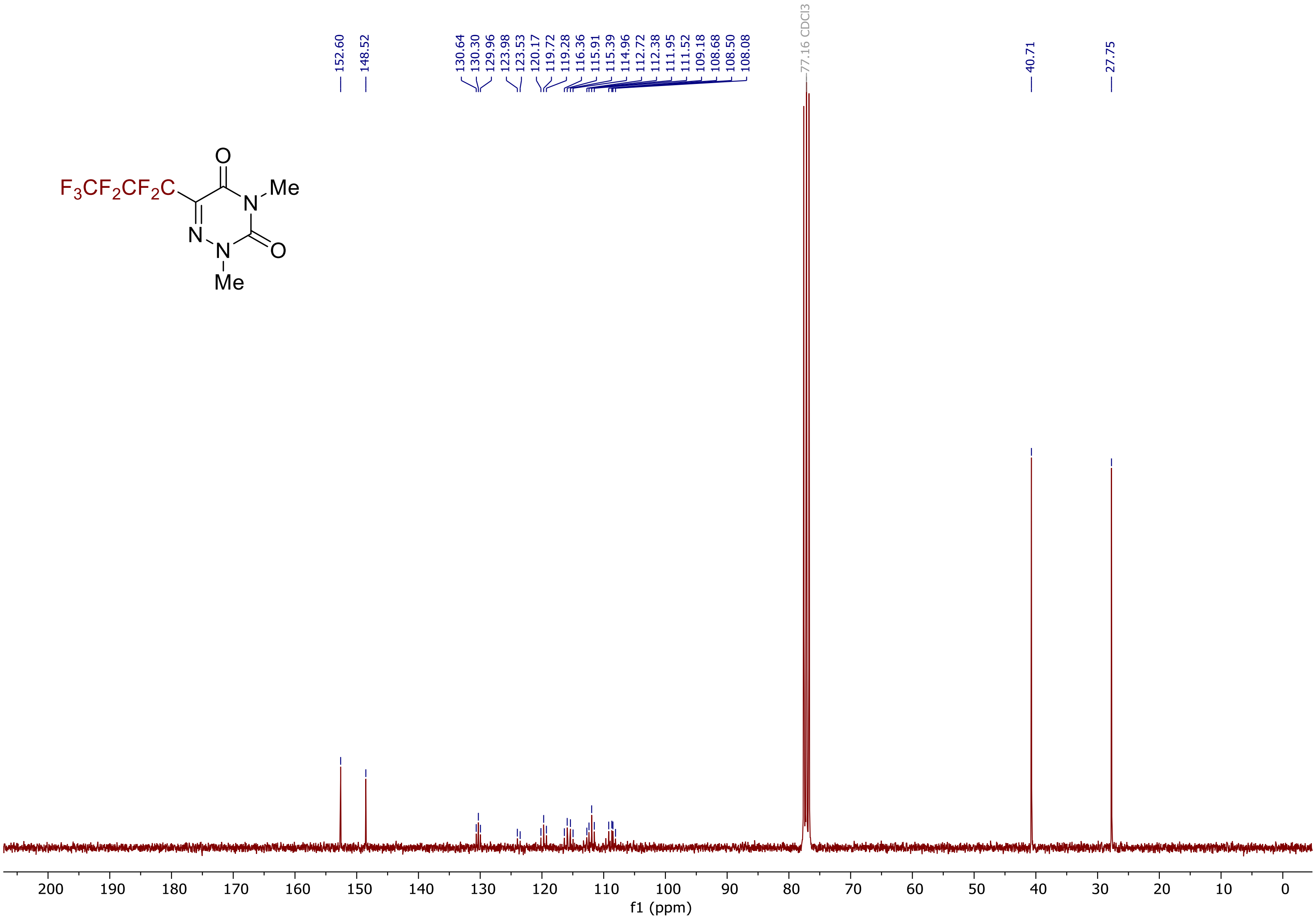

^{19}F NMR (282 MHz, $CDCl_3$)

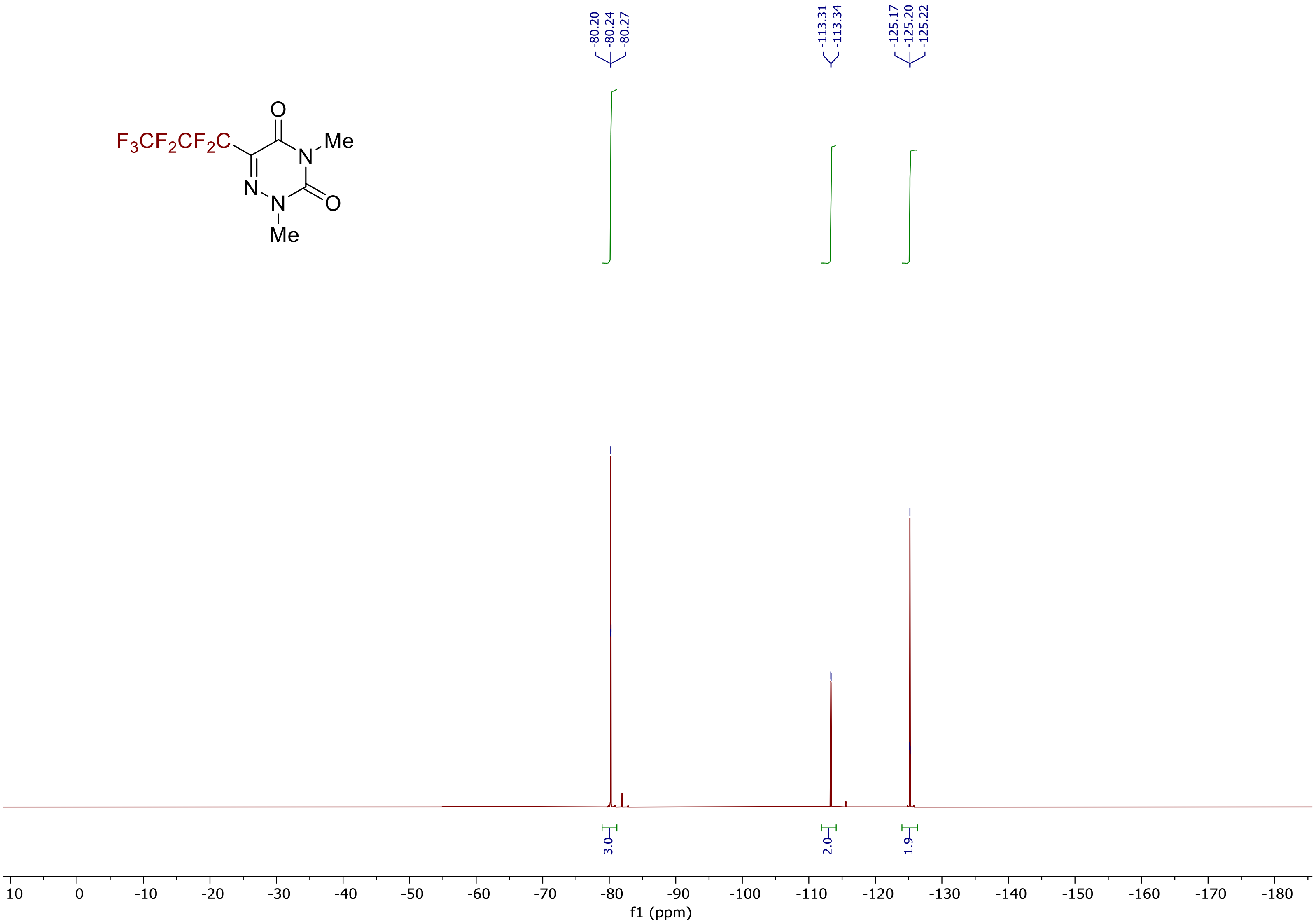

Methyl 1,3-dimethyl-2,6-dioxo-5-(trifluoromethyl)-1,2,3,6-tetrahydropyrimidine-4-carboxylate (14)

^1H NMR (300 MHz, $CDCl_3$)

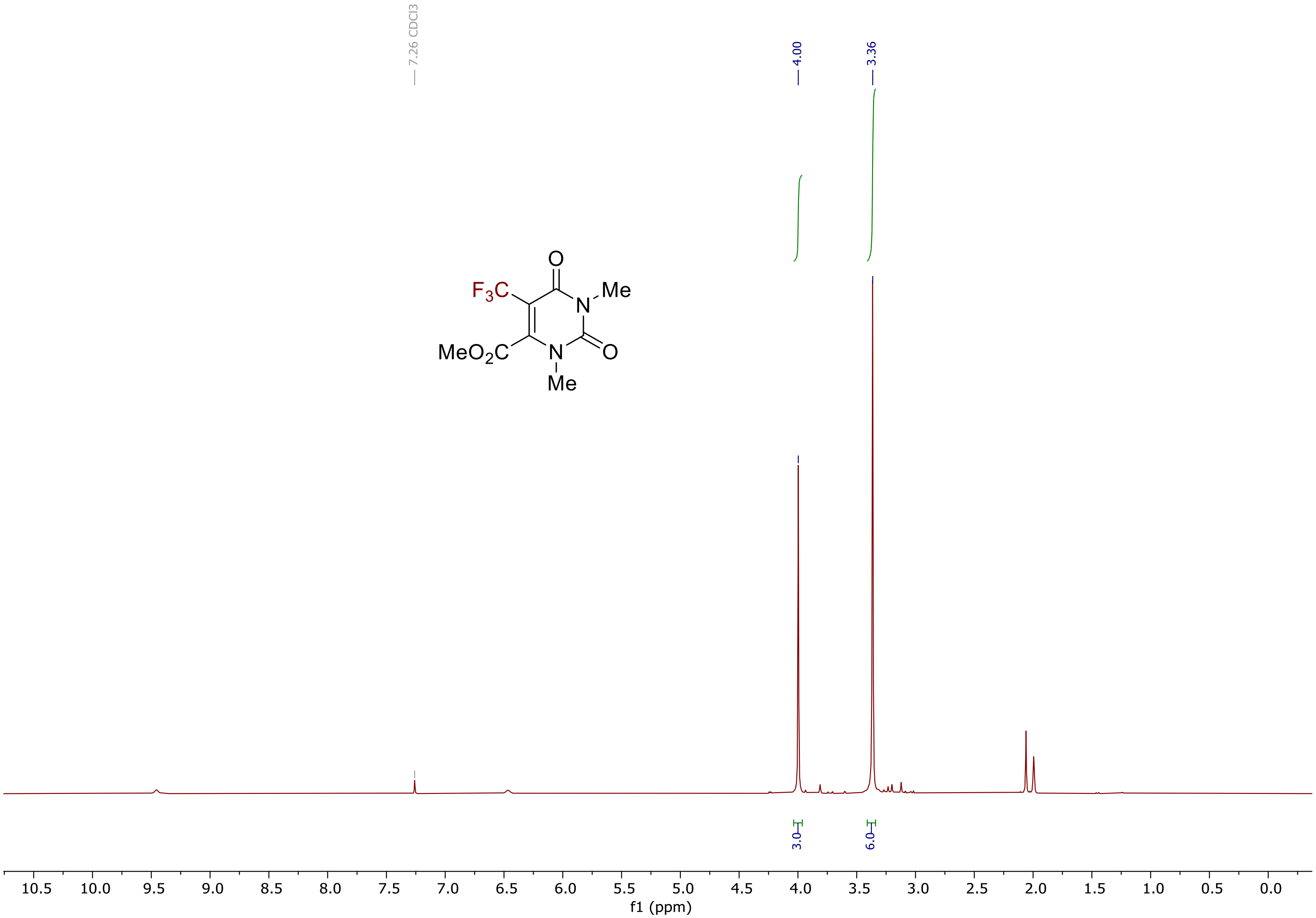

^{13}C NMR (75 MHz, $CDCl_3$)

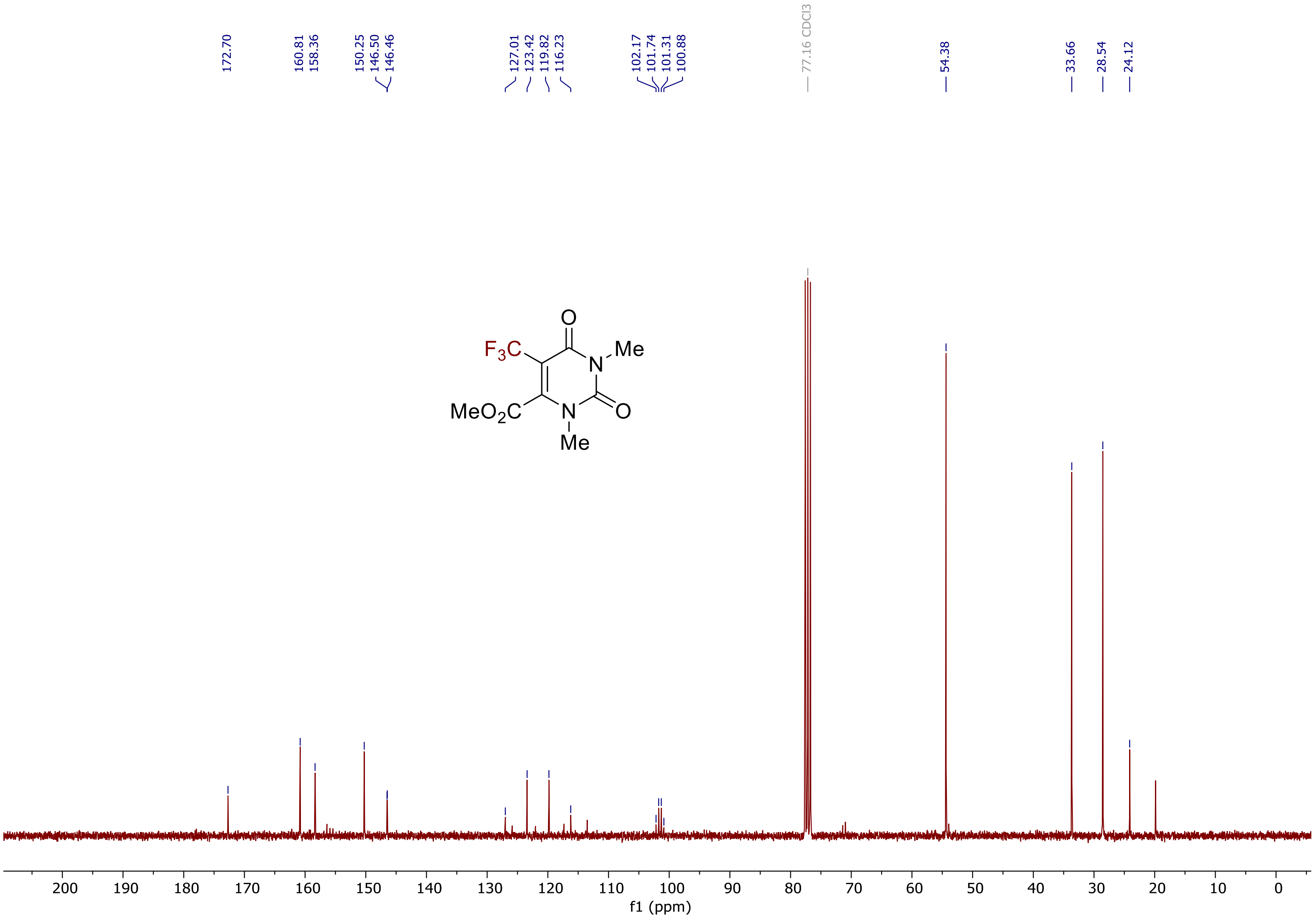

^{19}F NMR (282 MHz, $CDCl_3$)

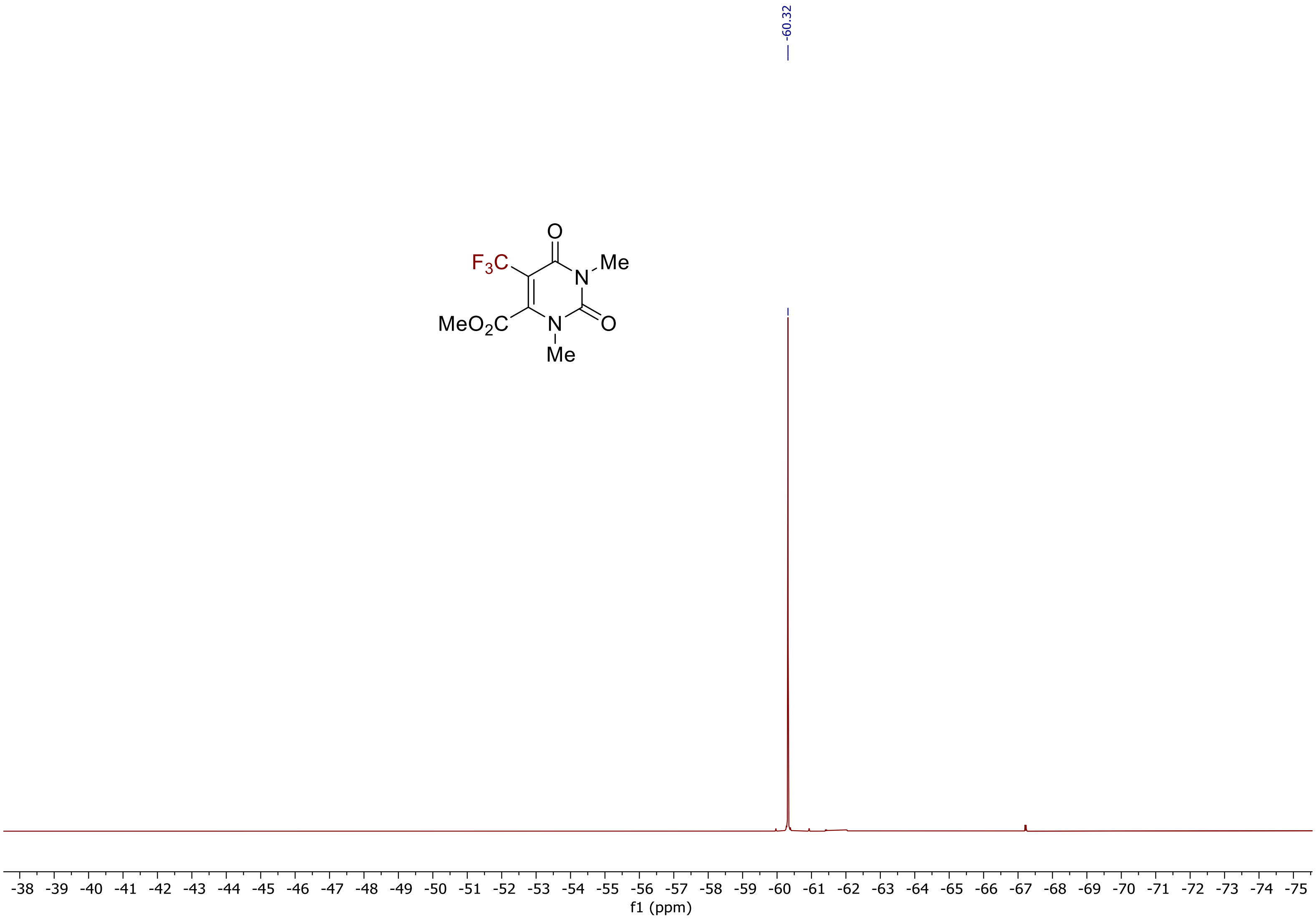

7-(1,3-Dioxolan-2-yl)-1,3-dimethyl-8-(trifluoromethyl)-3,7-dihydro-1*H*-purine-2,6-dione (15)

^{1}H NMR (300 MHz, $CDCl_3$)

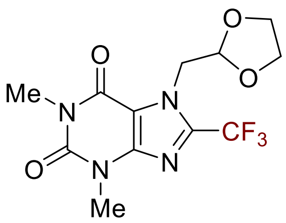

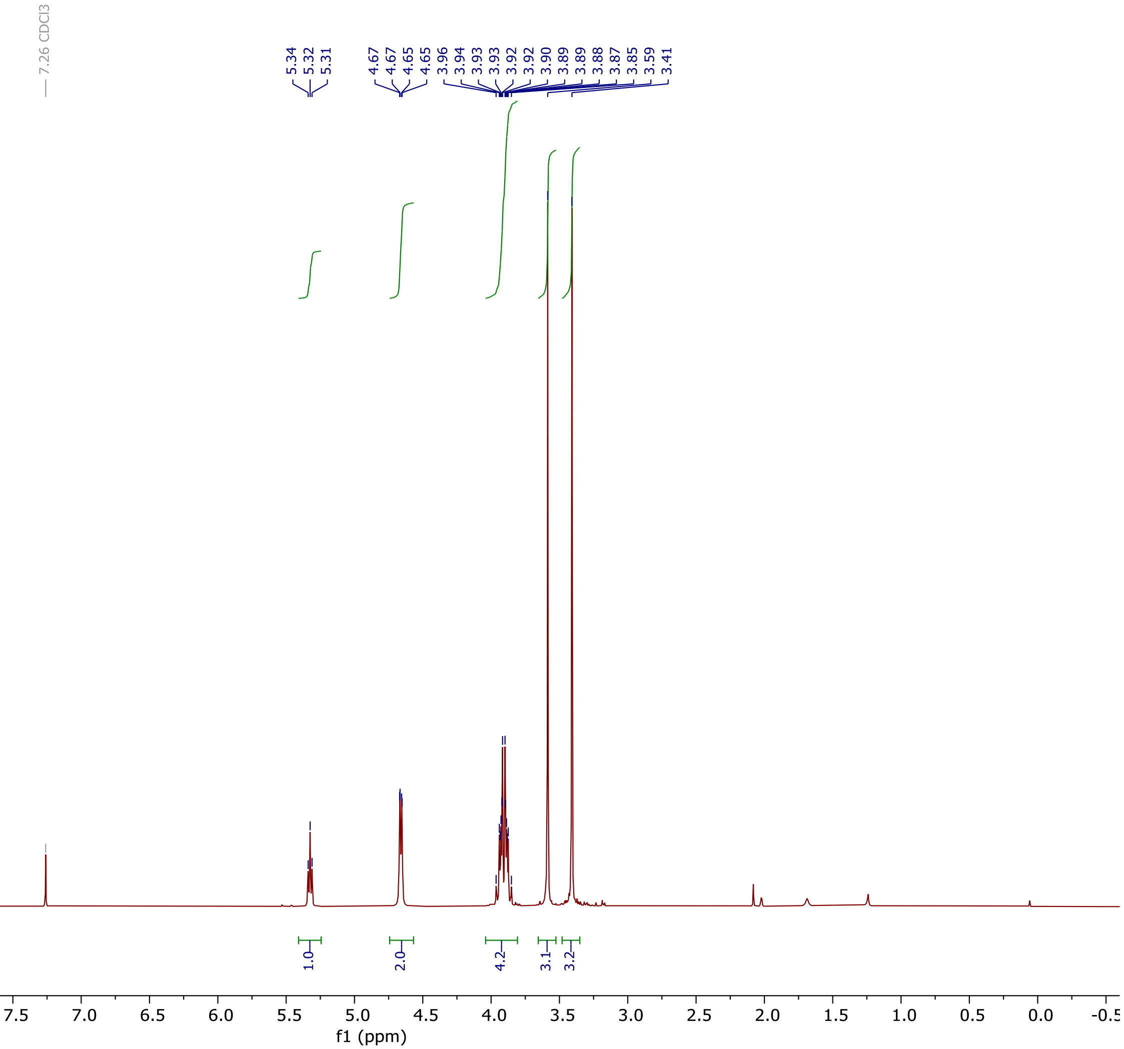

^{13}C NMR (75 MHz, $CDCl_3$)

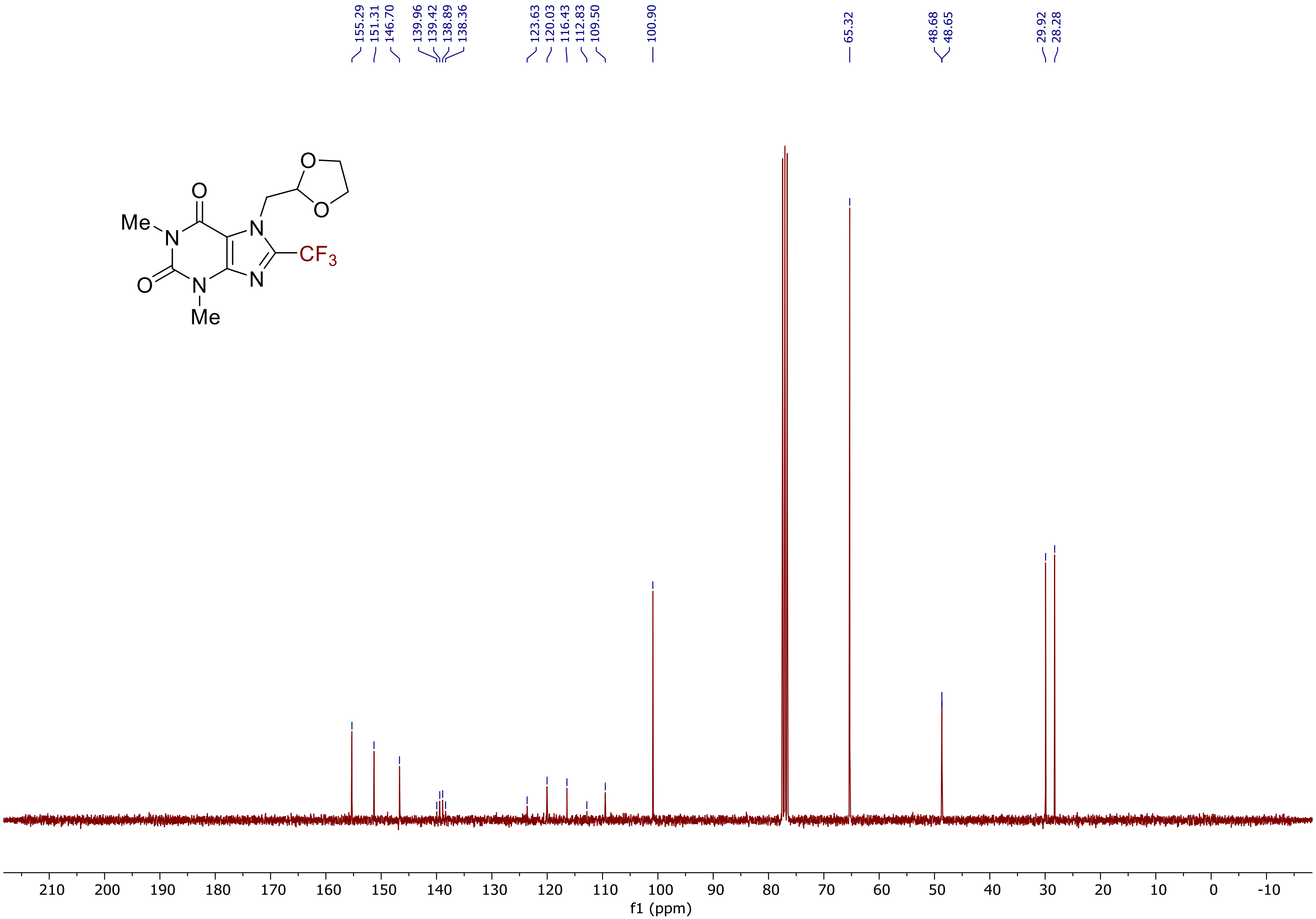

^{19}F NMR (282 MHz, $CDCl_3$)

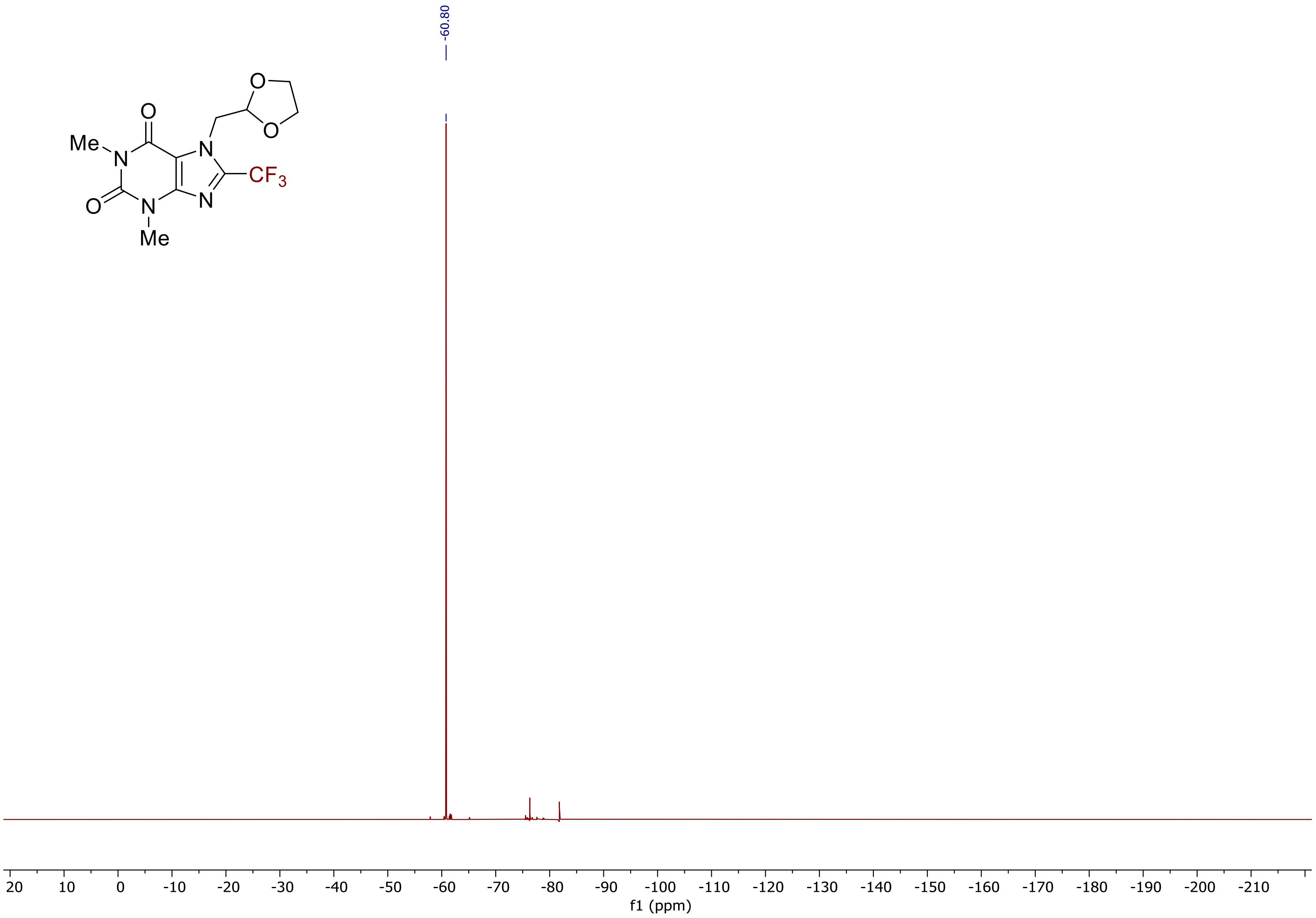

3,7-Dimethyl-1-(5-oxohexyl)-8-(trifluoromethyl)-3,7-dihydro-1*H*-purine-2,6-dione (16)

^{1}H NMR (300 MHz, $CDCl_3$)

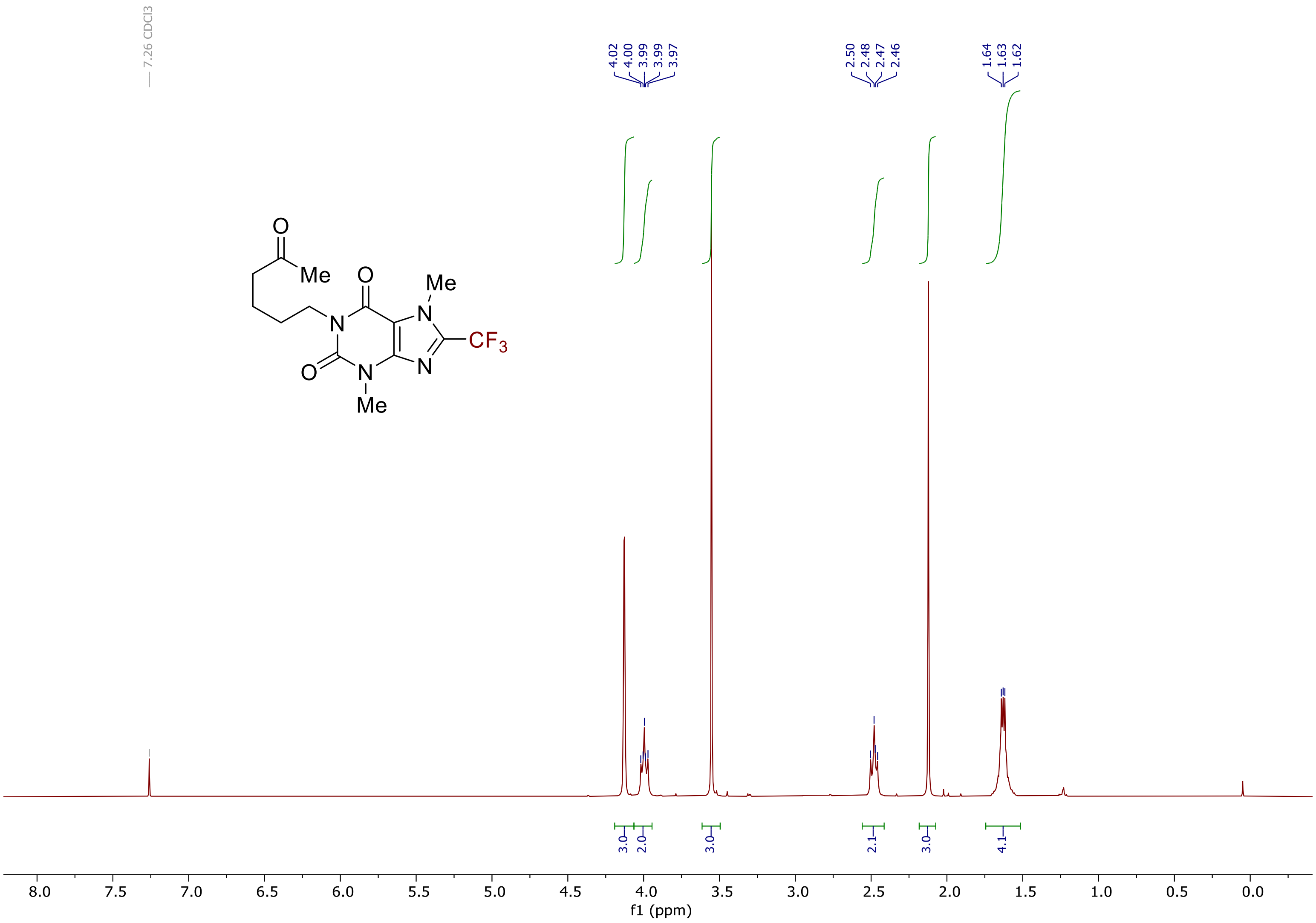

^{19}F NMR (282 MHz, $CDCl_3$)

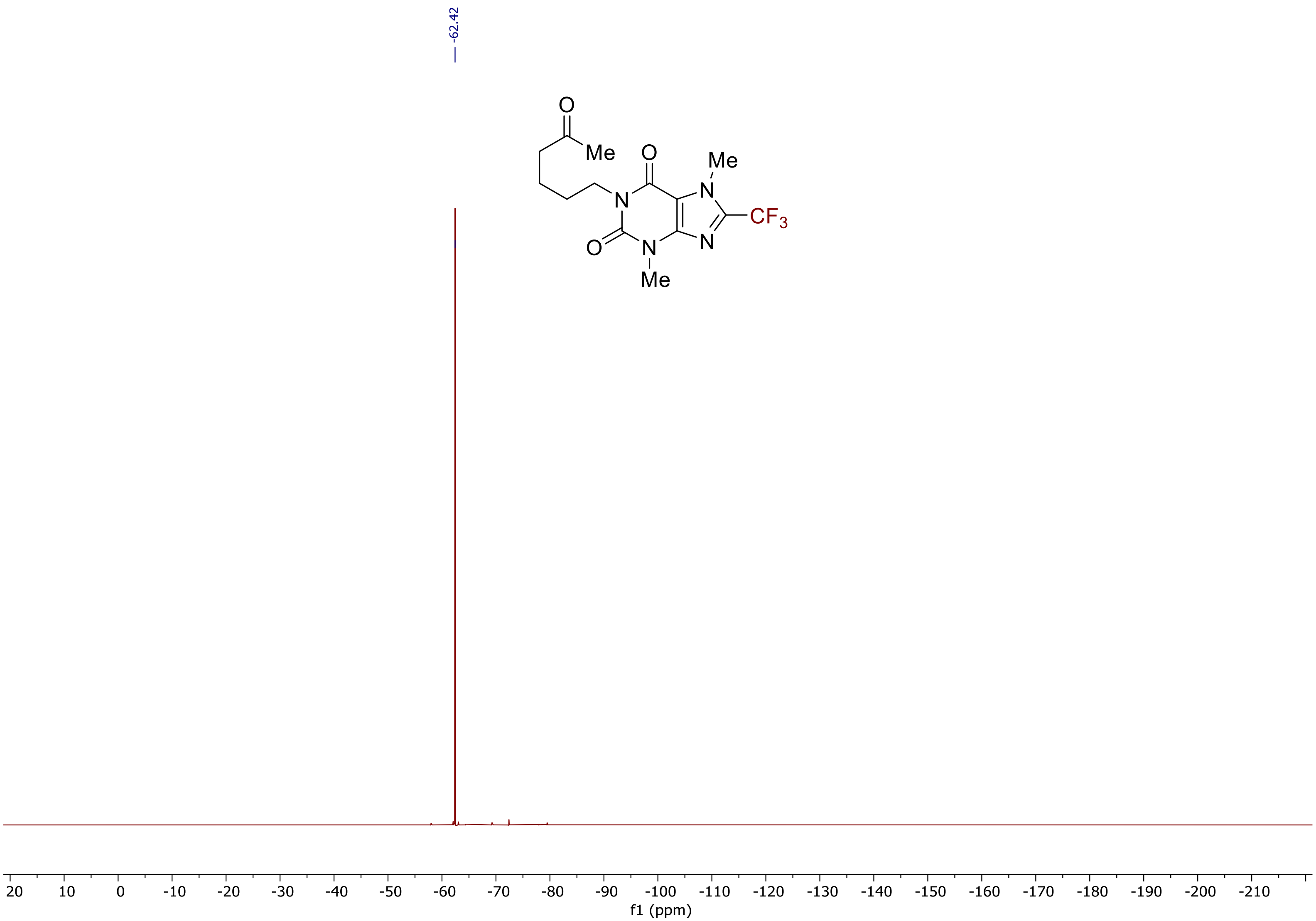

3-Methyl-2-(methylthio)-5-(trifluoromethyl)pyrimidin-4(*3H*)-one (17)

^{1}H NMR (300 MHz, $CDCl_3$)

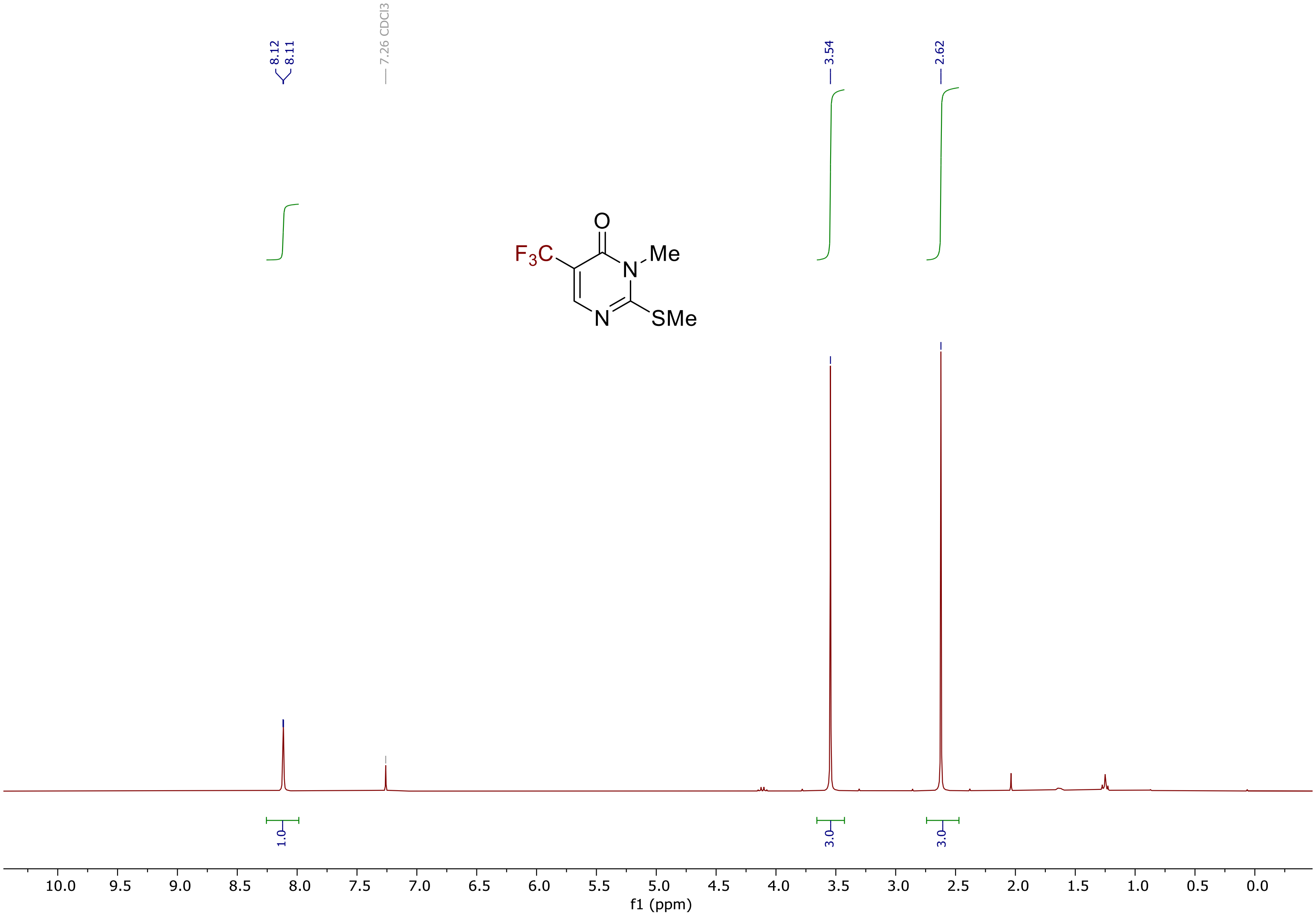

^{13}C NMR (75 MHz, $CDCl_3$)

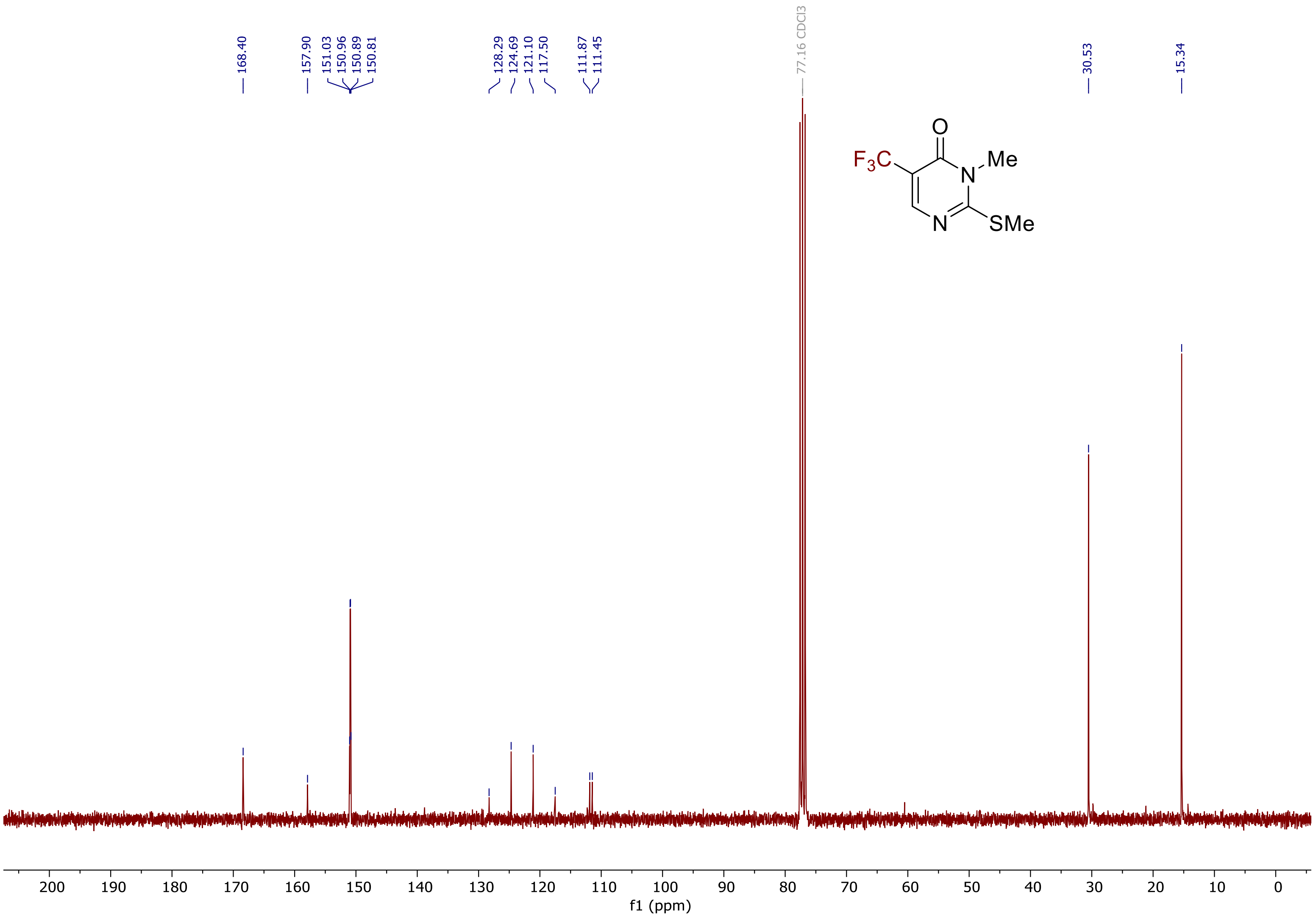

^{19}F NMR (282 MHz, $CDCl_3$)

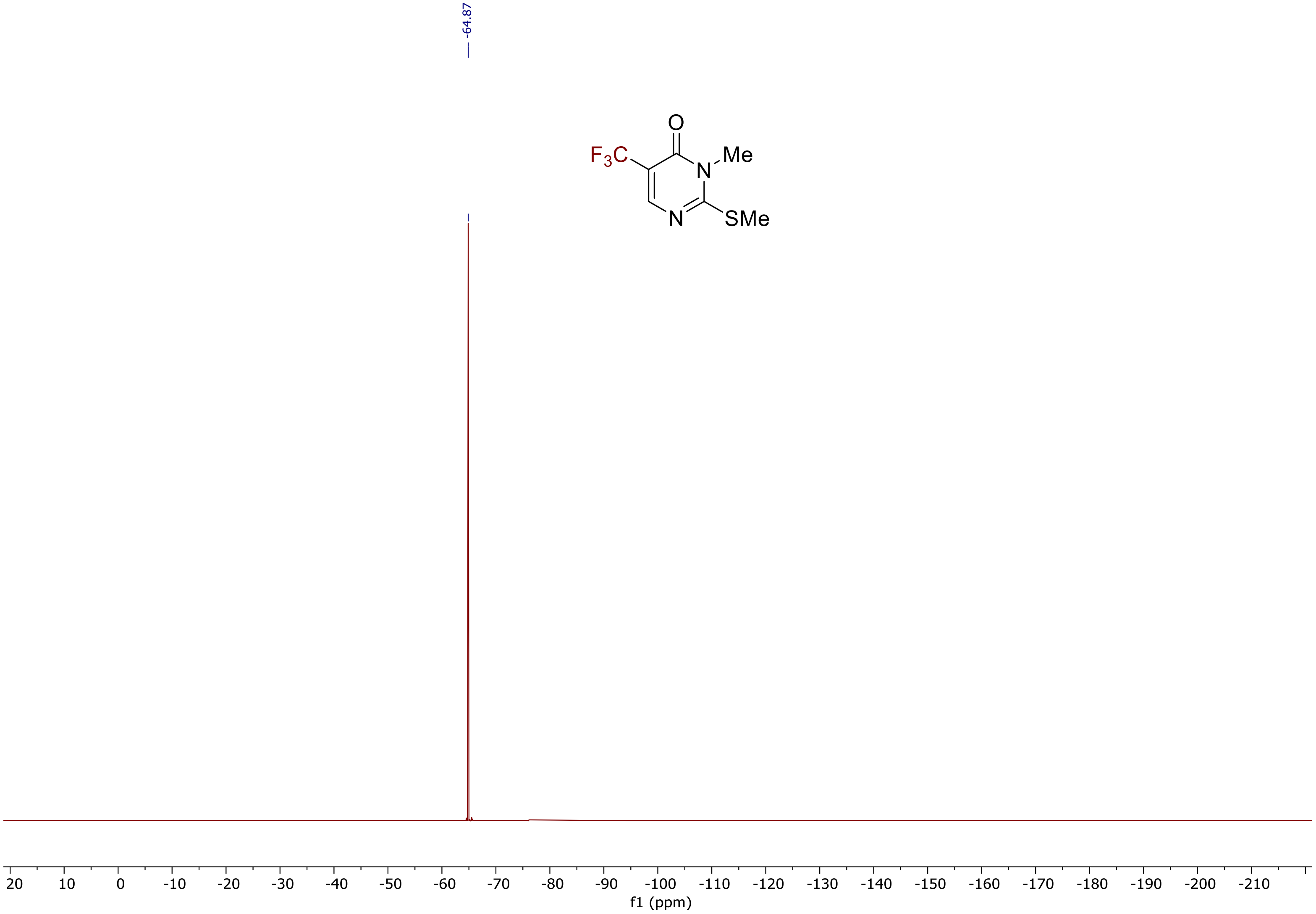

3,3,3-Trifluoro-1-(4-ethoxyphenyl)propan-1-one (19)

^{1}H NMR (300 MHz, $CDCl_3$)

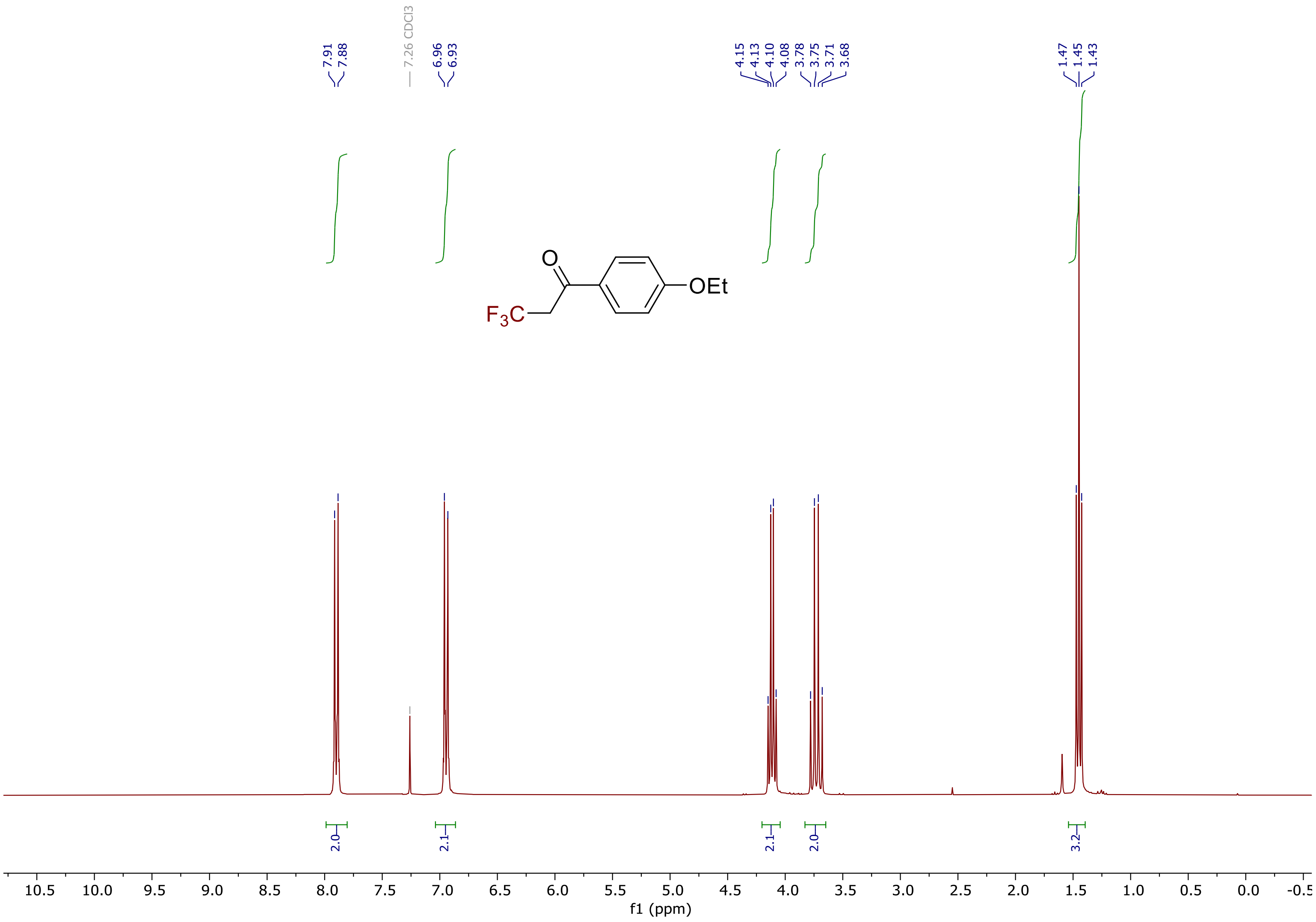

^{13}C NMR (75 MHz, $CDCl_3$)

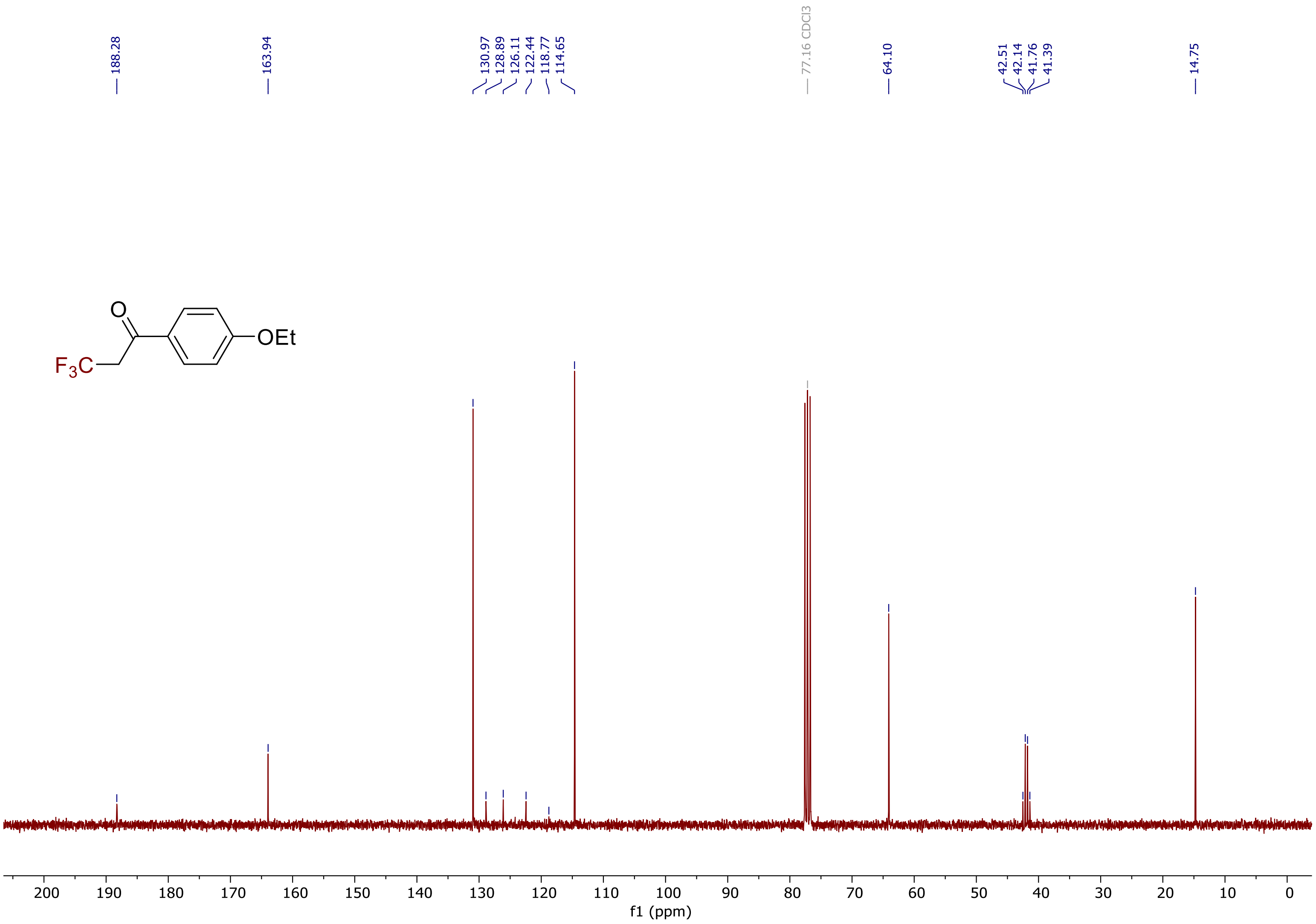

^{19}F NMR (282 MHz, $CDCl_3$)

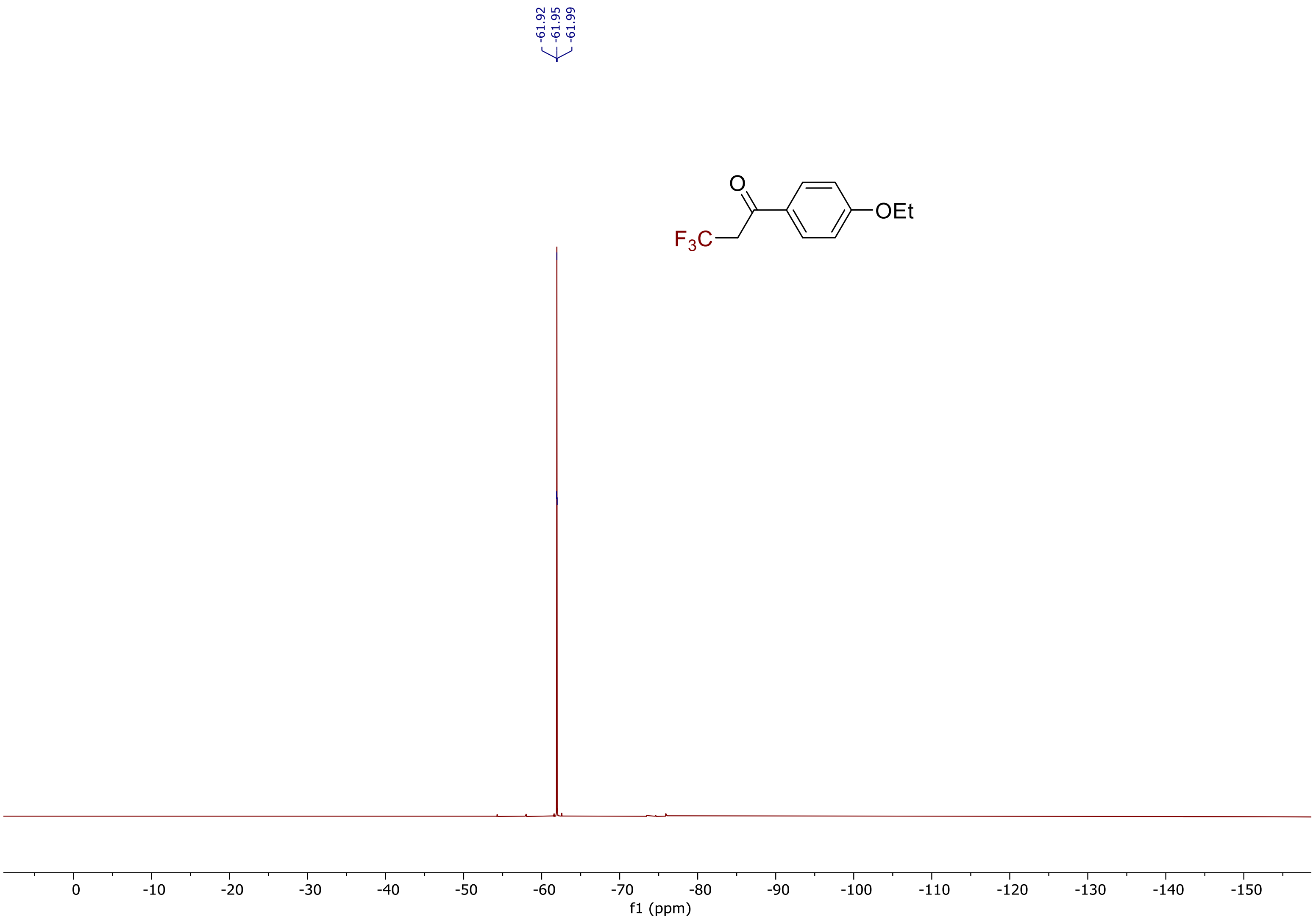

Supplement: Supplementary file 1 [file cs5c07973_si_001.pdf]
